# Synthesis of triarylpyridines with sulfonate and sulfonamide moieties via a cooperative vinylogous anomeric-based oxidation

**DOI:** 10.1038/s41598-021-95830-w

**Published:** 2021-08-19

**Authors:** Morteza Torabi, Mohammad Ali Zolfigol, Meysam Yarie, Behrouz Notash, Saeid Azizian, Mina Mirzaei Azandaryani

**Affiliations:** 1 Department of Organic Chemistry, Faculty of Chemistry, Bu-Ali Sina University, 6517838683 Hamedan, Iran; 2 Department of Inorganic Chemistry and Catalysis, Shahid Beheshti University, Evin, Tehran, Iran; 3 Department of Physical Chemistry, Faculty of Chemistry, Bu-Ali Sina University, 6517838683 Hamedan, Iran

**Keywords:** Chemistry, Catalysis, Organic chemistry, Chemical synthesis

## Abstract

Herein, novel magnetic nanoparticles with pyridinium bridges namely Fe_3_O_4_@SiO_2_@PCLH-TFA through a multi-step pathway were designed and synthesized. The desired catalyst and its corresponding precursors were characterized with different techniques such as Fourier transform infrared (FT-IR) spectroscopy, ^1^H NMR, ^13^C NMR, Mass spectroscopy, energy dispersive X-ray (EDX) analysis, thermogravimetric/derivative thermogravimetry (TG/DTG) analysis, scanning electron microscopy (SEM), transmission electron microscopy (TEM), and vibrating sample magnetometer (VSM). In addition, the catalytic application of the prepared catalyst in the synthesis of new series of triarylpyridines bearing sulfonate and sulfonamide moieties via a cooperative vinylogous anomeric-based oxidation was highlighted. The current trend revealed that the mentioned catalyst shows high recoverability in the reported synthesis.

## Introduction

With one glimpse at the literature, it can be found that urea and its derivatives have an extended domain of applications in agriculture, pharmaceuticals, petrochemicals, resin precursors, dyes and drugs^1–4^. Protein tyrosine kinases (PTKs), vascular endothelial growth factor receptor (VEGF), platelet-derived growth factor receptor (PDGFRB) and NADH oxidase are just a few of the biological applications of urea derivatives^5^. Substituted urea also act as versatile organo-catalyst in organic synthesis as they applied as acidic and basic catalysts, coupled with metals, polymer-based catalysts and supported catalysts^6^. The prevalent methods reported for the synthesis of urea derivatives are originally based on phosgene and isocyanates^3,7–20^. Recently, we have comprehensively reviewed the applications of biological urea-based catalysis in chemical processes^6^.

Pyridine as the heart of heterocycle chemistry represents many pharmaceuticals and agrochemicals applications. Indeed, pyridine systems with their unique functionalities such as inhibiting HIV protease, anti-depressant, anti-inflammatory, anti-viral, anti-hypertensive, anti-oxidant, anti-fungal, anesthetics, cholagogue, pesticides, dyes-paints, treating hypotension or hypertensionare are an integral part of medicinal and biological chemistry^14–27^. In addition to the above, pyridine derivatives perform a key role in the area of catalysis. Pyridine-based catalysts have been explored as metal complex catalysis^28^, chiral ligands for asymmetric catalysis^29^, molecular machines such as rotaxanes and catenanes^30–32^, organo-catalysis^33^, polymer-based catalysts^34^ and ionic liquids^35^.

Particularly, triarylpyridine derivatives, a notable subset of pyridine family, are emerging as an important class of heterocyclic compounds that have many biological properties that contain anti-cancer^36^, anti-depressant^3738^ and anti-bacterial^38^ activities. Moreover, triarylpyridines have been applied as chemosensors^39^, ligands^40^, and important intermediates for directional synthesis of insecticides, and surfactants^41^. Some of the synthetic routes of triarylpyridines include cyclocondensation reaction of an aldehyde, ketone and an ammonium salt as the nitrogen source^42^, cyclocondensation of ketone oxime/oxime acetates with aldehydes^43^ or ketone with benzyl halides^44^. Nevertheless, we are trying to synthesis a new library of triarylpyridines bearing sulfonate and sulfonamide moieties.

In spite of extensive research work on magnetic nanoparticles (MNPs), they are still of interest to many scientists in various fields. The innovative rational design of organic ligands supported on heterogeneous substrates is highly promising. Immobilization of the catalytically active homogeneous species on proper heterogeneous support materials leads to formation of new catalytic systems which have the characteristics of both categories^45–50^. To a large extent, MNPs are reported as efficient supports for immobilizing homogeneous catalysts. From an economic perspective, “catalyst activity” and “catalyst separation” are vital items in modern industrial catalytic processes^51–56^. MNPs, due to high surface area compare to bulk structures, low toxicity, the capability of surface modifications and easy dispersion are dramatically very suitable for catalytic processes. Furthermore, a suitable combination of the features of MNPs and unique properties of ionic moiety can bring more benefits. Ionically tagged magnetic nanoparticles have been applied in many organic reactions^57–65^.

**Scheme 1:**
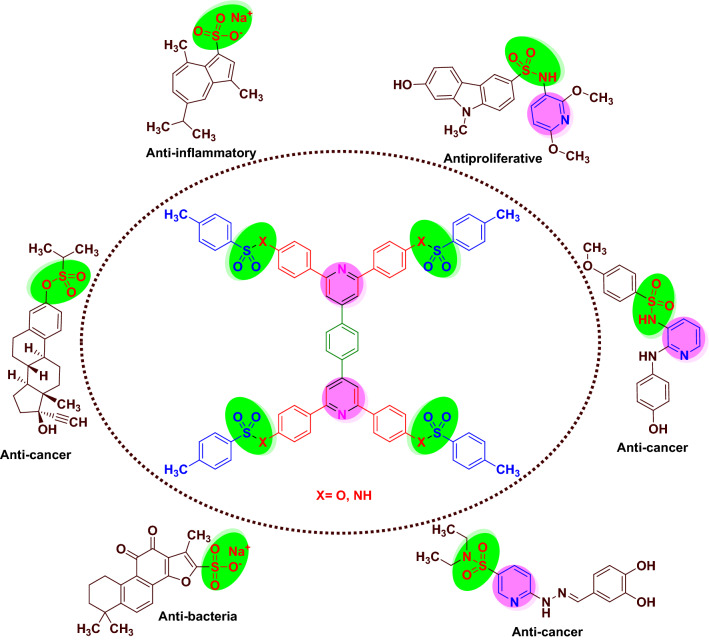
Selected examples of biological applications of molecules with sulfonates and sulfonamides moieties in comparison of our investigation.

Sulfonates are widespread in nature, and make up most of the sulfur dimension of aerobic soils. Many microorganisms can use sulfonates as a source of sulfur for growth, even when they are unable to metabolize the carbon skeleton of the compounds. Sulfonates have important pharmacological applications such as anti-oxidant, anti-inflammation and anti-apoptosis. Sometimes, it is necessary to transform drugs into their salts in order to improve physical properties such as solubility and stability. Sulfonate salts are used for this purpose. These materials are also found in many azo dyes. These compounds with different applications are reported in the literature (Scheme 1)^66–70^.

Sulfonamides as the largest family of pharmaceutical compounds are frequently seen in medicinal chemistry. Sulfonamides is the first antibiotics that be used systemically and is a nice source of revolution of antibiotics in biomedicine. Many derivatives of these compounds have also been applied in agriculture. Anti-bacterial, anti-inflammatory, anti-fungal, anti-protozoal, HIV protease inhibitory, anti-Alzheimer and anti-cancer are just a small part of the medicinal applications of molecules with sulfonamide moieties. There are many reports for synthesis of sulfonamides in the literature (Scheme 1)^71–80^.

**Scheme 2:**
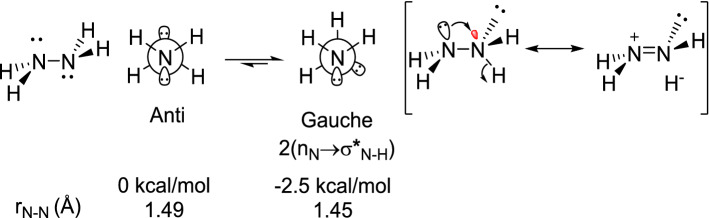
The preference for gauche conformation of hydrazine originates from two n_N_ → σ*_N–H_ stabilizing interactions.

**Scheme 3:**
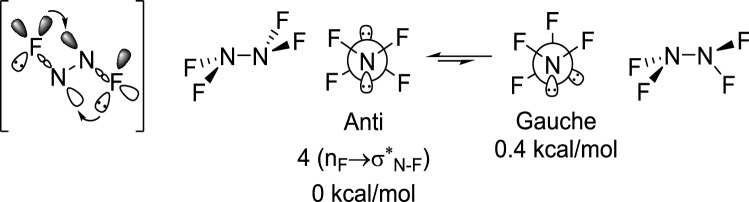
Fluorination reverses the conformational preference through four n_F_ → σ*_N-F_ stabilizing homoanomeric interactions.

In addition to the critical role of anomeric effect for the explanation of unusual observations in structure and reactivity of oxygen-containing molecules^81–85^, this important stereoelectronic effect demonstrates itself as a powerful phenomenon for justifying the weird results in various heteroatom‐rich compounds such as hydrazine and tetrafluorohydrazine^86–89^. In the case of hydrazine, the gauche-conformer is more stable than the anti-conformer. This observation originates from the stabilizing anomeric interactions of lone pair electrons of the nitrogen atom with a vacant antibonding orbital of adjacent N–H bond (n_N_ → σ*_N–H_). These interactions are not possible in anti-conformer (Scheme 2). On the other hand, in the case of tetrafluorohydrazine the anti-conformer is more stable than the gauche conformation due to homoanomeric interactions. In the anti-conformer, instead of n_N_ → σ*_N-F_ interactions, four n_F_ → σ*_N-F_ homoanomeric interactions are operative and control the stability of conformers (Scheme 3)^90^.

**Scheme 4:**
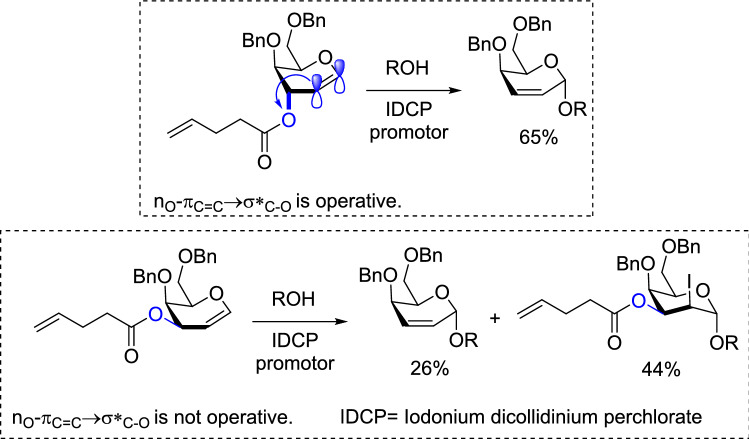
The role of vinylogous anomeric effect in promotion of iodonium-catalyzed Ferrier rearrangement.

In the vinylogous anomeric effect, as an important subclass of anomeric effect, electron transfer interactions occurred through C = C bonds. This phenomenon has attracted the attention of many researchers^91–99^. Vinylogous anomeric effect can control the chemical reactivity. For example, Ferrier rearrangement facilitated when the leaving group exists at pseudo-axial position. In this position vinylogous anomeric effect is on and the reaction promoted by n_O_-π_C=C_ → σ*_C–O_ interaction (Scheme 4)^100^.

**Scheme 5:**
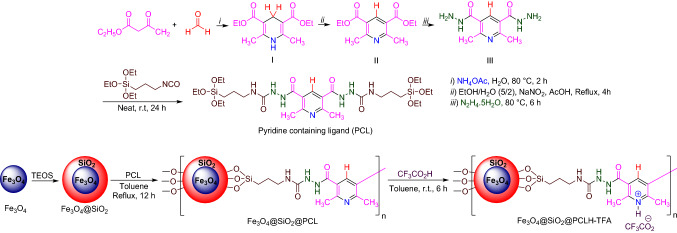
General experimental procedure for the targeted synthesis of Fe_3_O_4_@SiO_2_@PCLH-TFA.

**Scheme 6:**
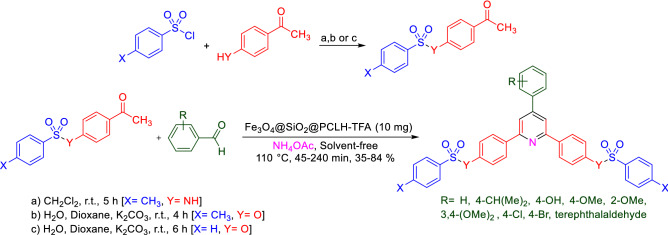
Synthesis of a new library of triarylpyridines bearing sulfonate and sulfonamide moieties in the presence of Fe_3_O_4_@SiO_2_@PCLH-TFA.

Herein, we pursue our previous works for the synthesis of pyridines^101–107^ and reported the synthesis and catalytic performance of a novel pyridinium tagged magnetic nanoparticles namely Fe_3_O_4_@SiO_2_@PCLH-TFA, for the synthesis of the new library of triarylpyridines bearing sulfonate and sulfonamide moieties via a cooperative vinylogous anomeric-based oxidation mechanism^108–112^ (Schemes 5, 6). We believe that the co-existence of pyridine moiety and sulfonate or sulfonamide segment within the structure of a single molecule has a significant impact on the biological importance of these versatile structures.

## Results and discussion

Since that the pyridines with sulfonate and sulfonamide moieties have been considered as drug candidates, the synthesis of a new library of symmetrical triarylpyridines bearing sulfonate and sulfonamide moieties are our main research interest. Therefore, herein we wish to present a new catalytic system with pyridinium linkers for the synthesis of the above-mentioned compounds. According to Alabugin’s theory, which has introduced stereoelectronic effects as a bridge between structure and reactivity^86–89^, we have also applied the above-mentioned theory in the course of the reaction mechanism.

Divers’ techniques including FT-IR, XRD, EDX, SEM, TEM, VSM and TG/DTG were employed to validate the formation of Fe_3_O_4_@SiO_2_@PCLH-TFA.

**Figure 1:**
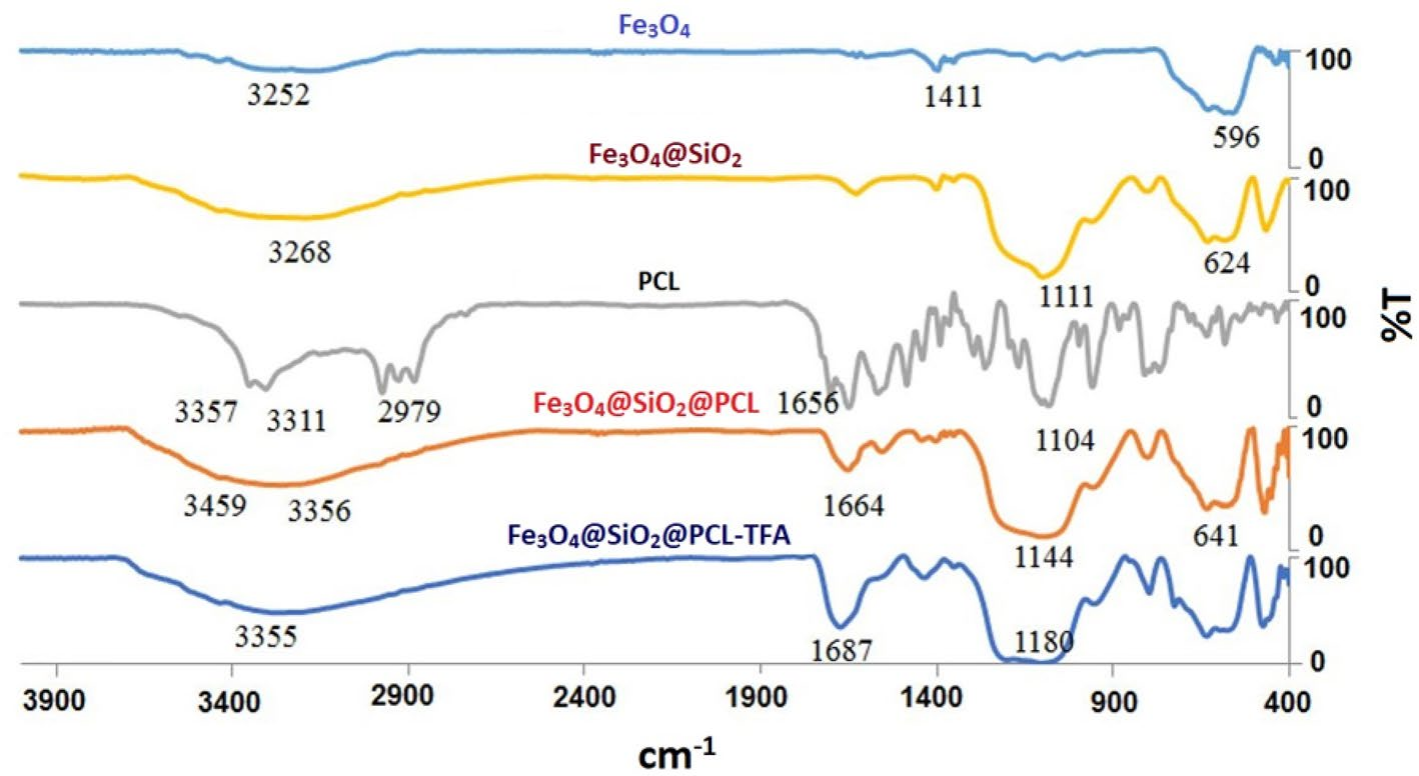
Comparative investigation of Fe_3_O_4_@SiO_2_@PCLH-TFA and its corresponding intermediates.

FT-IR spectrum of Fe_3_O_4_@SiO_2_@PCLH-TFA and related intermediates including Fe_3_O_4_, Fe_3_O_4_@SiO_2_, PCL and Fe_3_O_4_@SiO_2_@PCL were discussed in a comparative investigation. The changes made in each of the intermediates confirm their proper synthesis, which finally confirms the successful preparation of Fe_3_O_4_@SiO_2_@PCL. The diagnostic peak of Fe_3_O_4_ appeared at 633 cm^−1^ is related to the stretching vibration of Fe–O. In the FT-IR spectrum of Fe_3_O_4_@SiO_2_ the diagnostic peak at 1099 cm^−1^ is belonged to the Si–O–Si absorption band. The carbonyl groups related to the structure of the prepared catalyst appeared at around 1687 cm^−1^ as a broad peak. In the structure of desired catalyst, the broad peak at about 2500–3500 cm^−1^ is related to pyridinium moiety, amidic NH groups of the PCL and free hydroxyl groups of the Fe_3_O_4_@SiO_2_@PCLH-TFA (Fig. 1).

**Figure 2:**
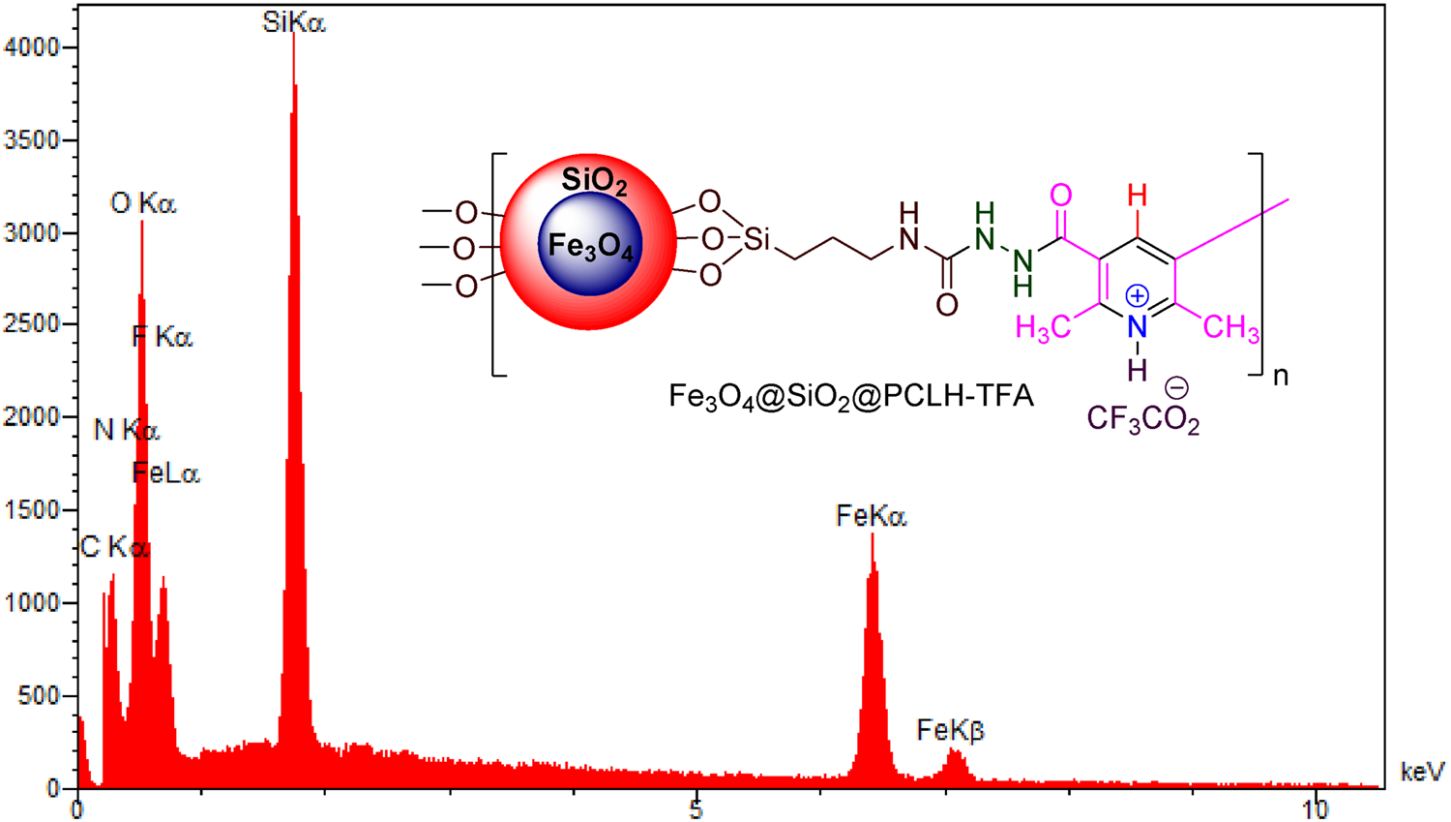
EDX analysis of Fe_3_O_4_@SiO_2_@PCLH-TFA.

**Figure 3:**
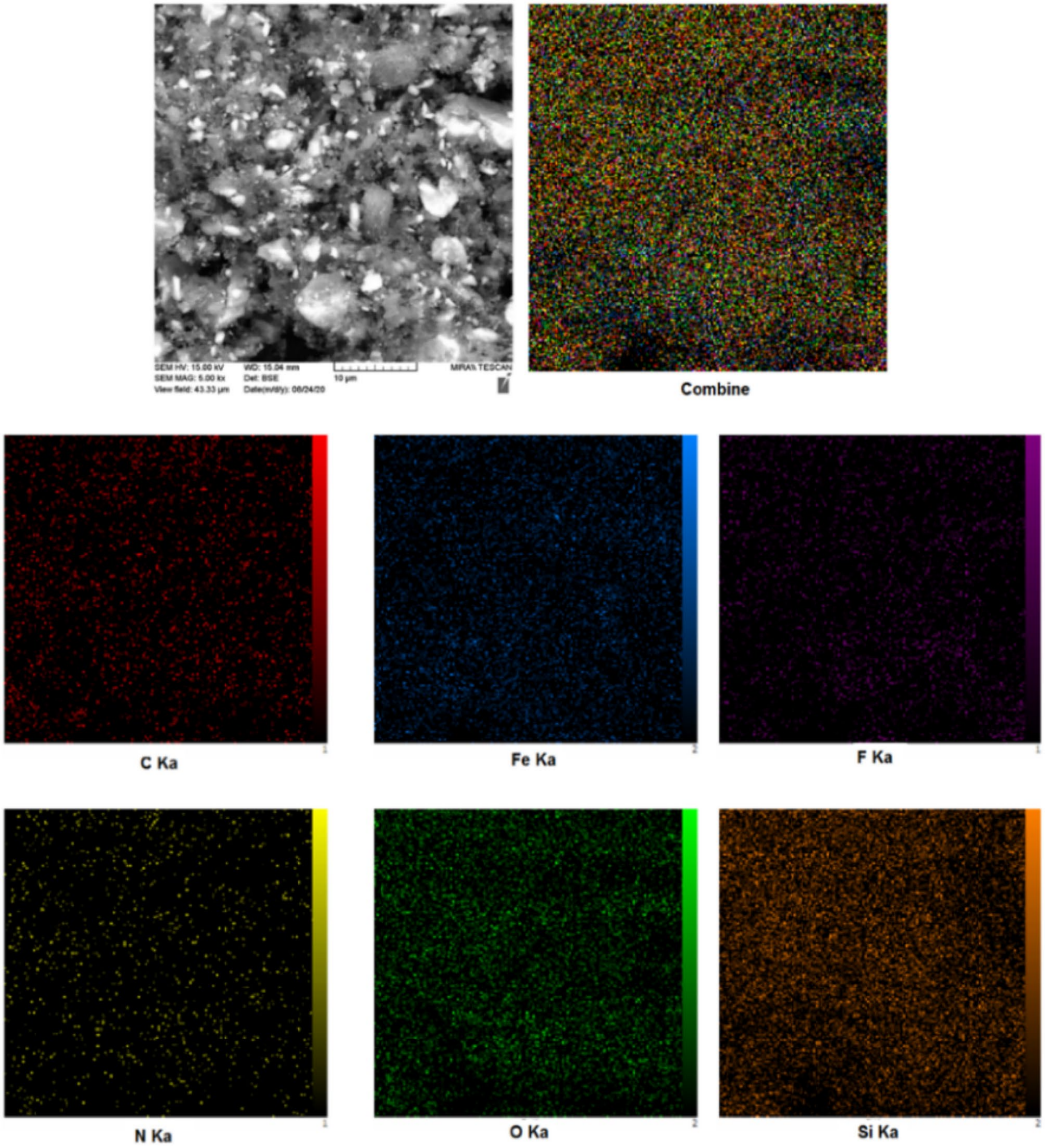
Elemental mapping analysis of Fe_3_O_4_@SiO_2_@PCLH-TFA.

The elemental composition of Fe_3_O_4_@SiO_2_@PCLH-TFA was characterized using energy-dispersive X-ray spectroscopy (EDX). According to the outcome data, all expected elements including iron, silicon, oxygen, carbon, nitrogen and fluorine were approved (Fig. 2). Furthermore, to confirm this observation, elemental mapping analysis was investigated (Fig. 3). The elemental mapping reveals uniform distribution of the mentioned elements.

**Figure 4:**
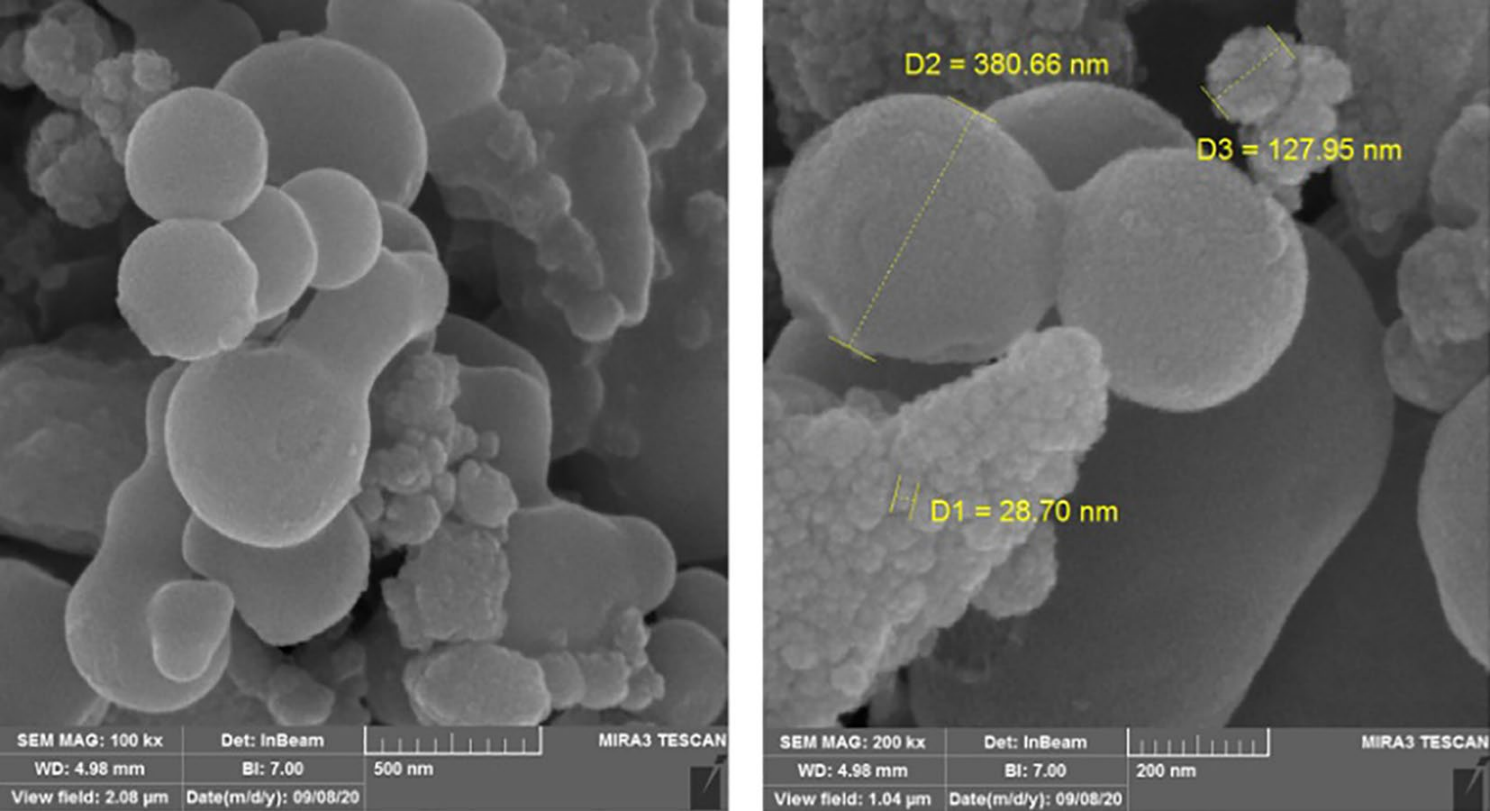
SEM images of Fe_3_O_4_@SiO_2_@PCLH-TFA.

**Figure 5:**
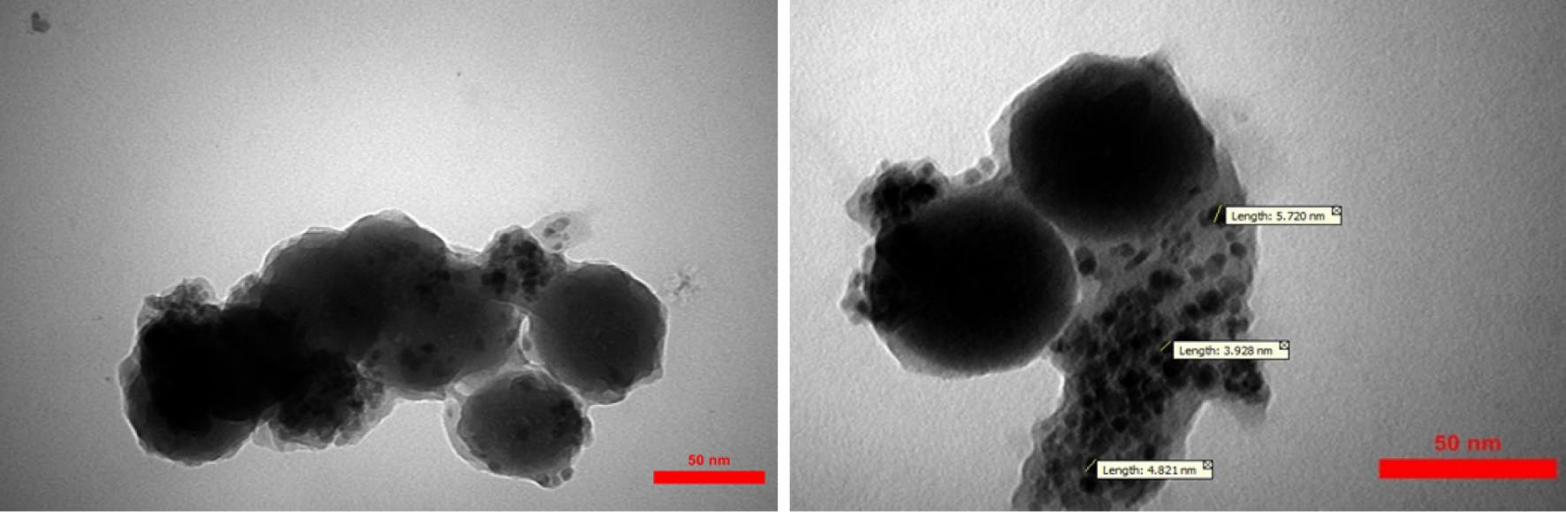
TEM images of Fe_3_O_4_@SiO_2_@PCLH-TFA.

For a better description of catalyst, the surface and morphology property analysis of Fe_3_O_4_@SiO_2_@PCLH-TFA was also studied by SEM images. According to the SEM images the morphology of the catalyst is spherical (Fig. 4). But by more focusing, it can be seen that each sphere is made from the aggregate of smaller size nanoparticles. For further clarification, the TEM images were recorded (Fig. 5) and it clearly shows that the bigger particles are made from nanopraticles (4–6 nm) and with core–shell structure. The TEM results confirmed the obtained data from SEM images (Fig. 5).

**Figure 6:**
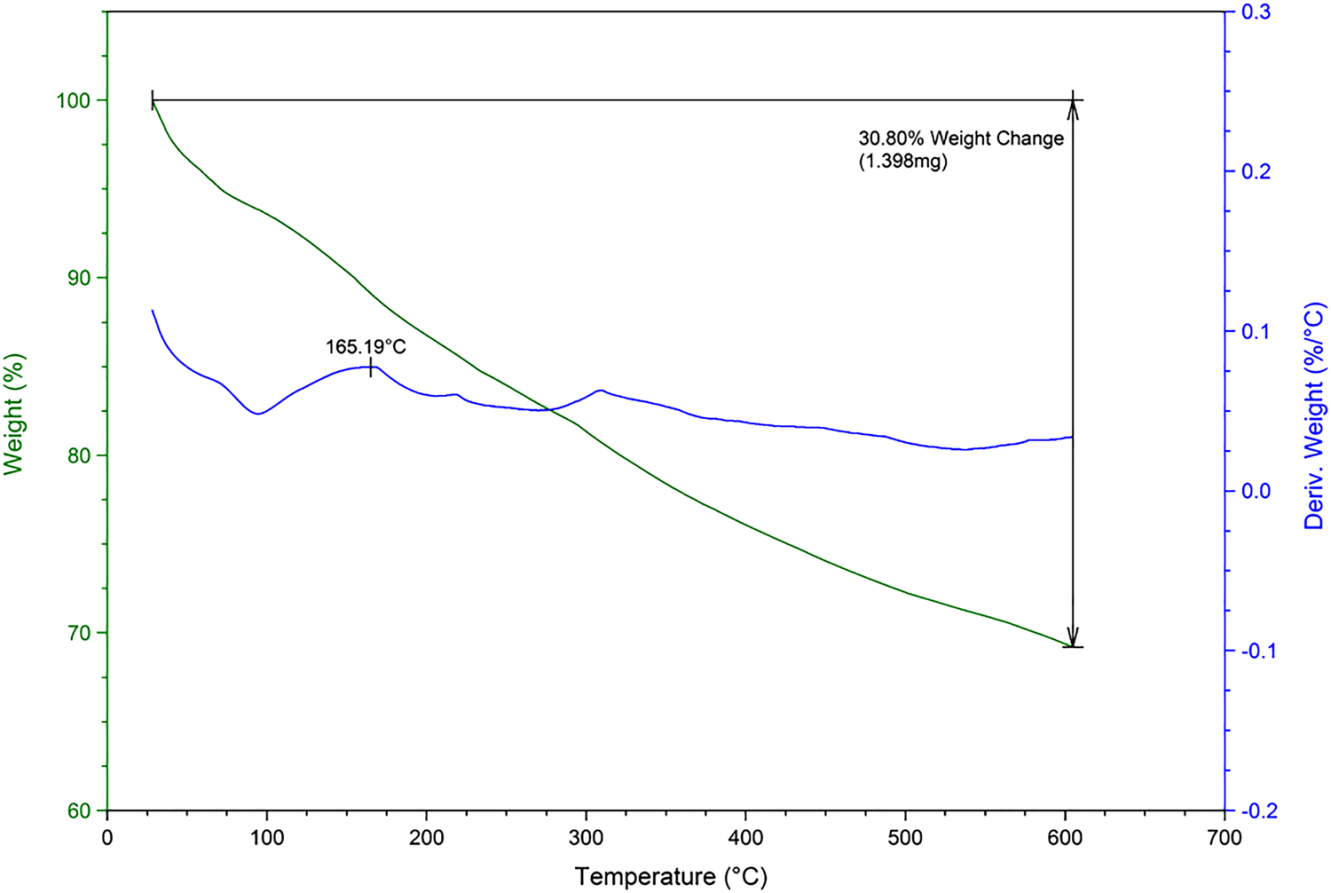
TG/DTG curve of Fe_3_O_4_@SiO_2_@PCLH-TFA.

TG has been generally applied to evaluate thermal degradation behaviour of magnetic nanoparticles. To determine the thermal behaviour of the Fe_3_O_4_@SiO_2_@PCLH-TFA, TG/DTG was carried out. The obtained results are illustrated in Fig. 6 and show good thermal stability for the prepared Fe_3_O_4_@SiO_2_@PCLH-TFA.

**Figure 7:**
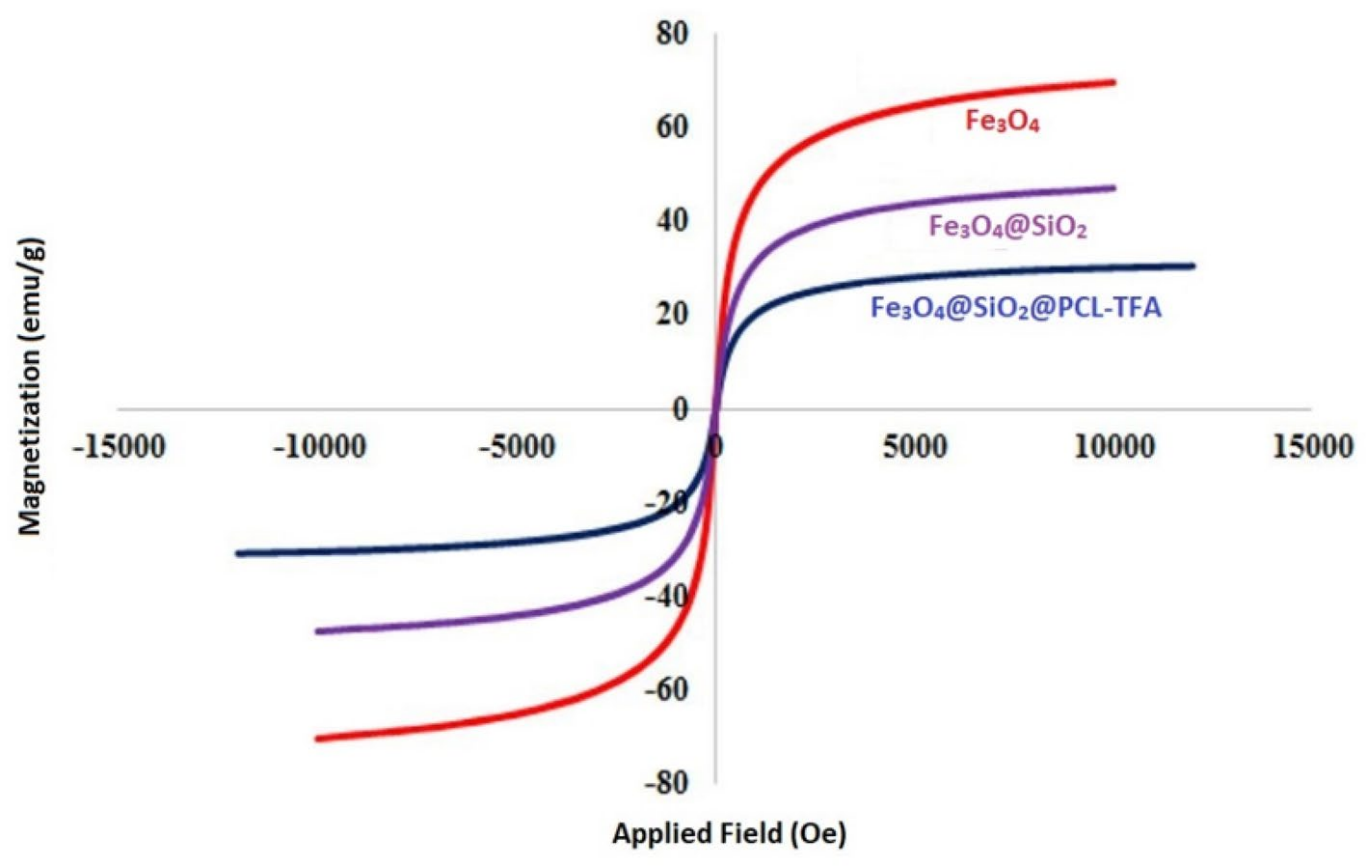
VSM curves of Fe_3_O_4_@SiO_2_@PCLH-TFA in comparison with its related magnetic precursors.

The VSM analysis was employed to determine magnetic properties of Fe_3_O_4_@SiO_2_@PCLH-TFA in comparison with its related precursors such as Fe_3_O_4_ and Fe_3_O_4_@SiO_2_. Decreasing the magnetic property of Fe_3_O_4_@SiO_2_@PCLH-TFA compared to its precursors, indicates the successful addition of organic moieties onto the surface of magnetic precursor (Fig. 7). The value of magnetic saturation of Fe_3_O_4_@SiO_2_@PCLH-TFA is about 25 emu/g which is enough for its easy separation from the reaction mixture upon completion of the catalytic reaction.

After targeted and multi-step synthesis and characterization of Fe_3_O_4_@SiO_2_@PCLH-TFA as a novel pyridinium tagged magnetic nanoparticles, we evaluated its catalytic performance for the synthesis of a new library of triarylpyridines bearing sulfonate and sulfonamide moieties via a cooperative vinylogous anomeric-based oxidation mechanism. For this purpose, we designed a model reaction for achieving the best operational reaction conditions. In this regard, the reaction of 4-choloro benzaldehyde, 4-acetylphenyl-4-methylbenzenesulfonate and ammonium acetate was selected and the role of solvents, temperature and amount of Fe_3_O_4_@SiO_2_@PCLH-TFA as catalyst was investigated. Based on resulted experimental data, the utilization of 10 mg of Fe_3_O_4_@SiO_2_@PCLH-TFA at 110 °C under solvent-free conditions is the best conditions for the synthesis of target molecules **1f.** from time and yield perspective (Table 1).

**Table 1:** Optimizing of the reaction condition for the synthesis of **1f.**


Entry	Solvent	Temperature (°C)	Catalyst loading (mg)	Time (min.)	Yield (%)^a^
1	–	120	10	45	80
2	–	110	20	45	84
**3**^**b**^	–	**110**	**10**	**45**	**84**
4		110	5	60	74
5	–	110	–	45	Trace
6	–	110	–	120	40
7	–	100	10	60	72
8	–	90	10	70	55
9	–	80	10	70	40
10	–	70	10	90	Trace
11	H_2_O	Reflux	10	180	–
12	EtOH	Reflux	10	180	Trace
13	*n*-Hexane	Reflux	10	180	–
14	EtOAc	Reflux	10	180	–
15	CH_2_Cl_2_	Reflux	10	180	–

Reaction conditions: 4-cholorobenzaldehyde (1 mmol, 0.140 g), 4-acetylphenyl-4-methylbenzenesulfonate (2 mmol, 0.580 g), and ammonium acetate (1.5 mmol, 0.115 g).

^a^Related to isolated yields.

^b^Data for the model reaction under air, and inert atmosphere (nitrogen and argon) are similar.

Also, we tested the model reaction (synthesis of molecule **1f.**) in the presence of related intermediates of Fe_3_O_4_@SiO_2_@PCL and some known catalysts to validate the importance of the existence of ionic tag (pyridinium site) within the structure of Fe_3_O_4_@SiO_2_@PCLH-TFA as the catalyst. As inserted in Table 2, the novel synthesized catalyst shows the best results in comparison with some other catalysts and verified the crucial role of the acidic and hydrogen-bond sites within the structure of the catalyst.

**Table 2:** Investigation of catalytic behavior of Fe_3_O_4_@SiO_2_@PCLH-TFA and its relative intermediates and some known catalysts upon the synthesis of 1f.

Entry	Catalyst	Load of catalyst	Yield (%)
1	Fe_3_O_4_	10 mg	30
2	Fe_3_O_4_@SiO_2_	10 mg	25
3	PCL	10 mol%	45
4	Fe_3_O_4_@SiO_2_@PCL	10 mg	58
5	Fe_3_O_4_@SiO_2_@PCLH-TFA	10 mg	84
6	Trifluoroacetic acid	10 mol%	55
7	*p*-Toluenesulfonic acid	10 mol%	80
8	FeCl_3_	10 mol%	33
9	Trityl chloride	10 mol%	Trace
10	H_2_SO_4_	10 mol%	Trace
11	NH_2_SO_3_H	10 mol%	65
12	Fe (HSO_4_)_3_	10 mol%	52
13	Al (HSO_4_)_3_	10 mol%	36
14	Ca (HSO_4_)_2_	10 mol%	44
15	Silica sulfuric acid^113^	10 mg	71

Reaction conditions: 4-Cholorobenzaldehyde (1 mmol, 0.140 g), 4-acetylphenyl-4-methylbenzenesulfonate (2 mmol, 0.580 g), and ammonium acetate (1.5 mmol, 0.115 g), solvent-free, 110 °C, 45 min.

In another study with considering the above-mentioned encouraging results under optimal reaction conditions, we generalized a catalytic procedure for the synthesis of a new library of triarylpyridines bearing sulfonate and sulfonamide moieties using Fe_3_O_4_@SiO_2_@PCLH-TFA as a novel recoverable catalyst. In this synthetic procedure, different ketones with sulfonate and sulfonamide moieties and various aromatic aldehydes bearing electron-withdrawing and electron-releasing substituents were utilized. Also, terephthaldehyde revealed promising results. The collected data are inserted in Table 3.

**Table 3:**
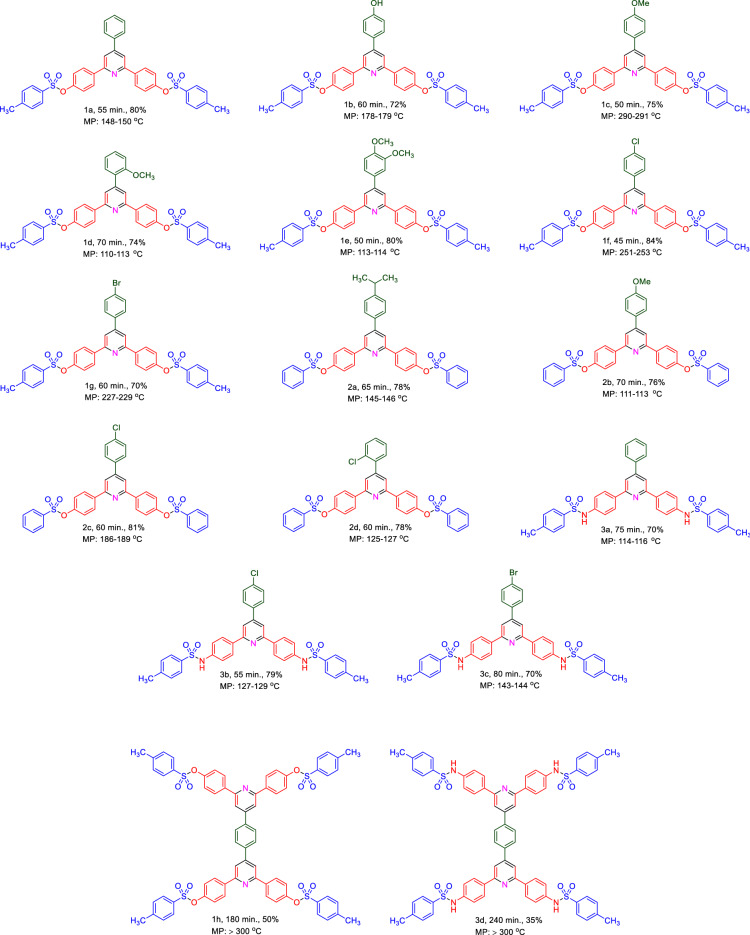
Synthesis a new library of triarylpyridine derivatives bearing sulfonate and sulfonamide moieties in the presence of Fe_3_O_4_@SiO_2_@PCLH-TFA.

Reaction conditions: aldehyde (1 mmol), methyl ketone (2 mmol) and ammonium acetate (1.5 mmol, 0.115 g), Solvent-free, 110 °C, catalyst = 10 mg, reported yields are referred to isolated yields.

**Scheme 7:**
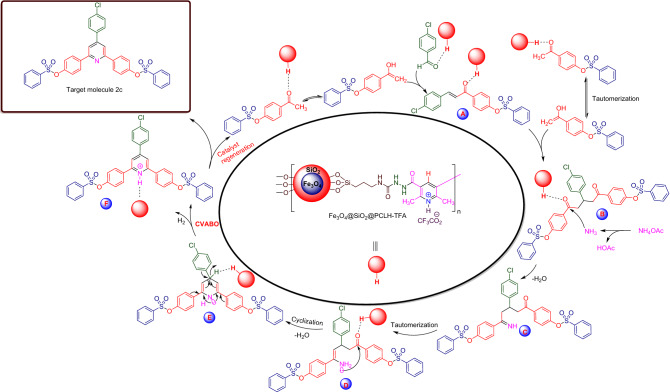
A suggested plausible mechanism for the synthesis of **2c** using Fe_3_O_4_@SiO_2_@PCLH-TFA as catalyst.

Also, a plausible mechanism for the synthesis of **2c** (Scheme 7) is suggested. At the first, 4-acetylphenyl-4-methylbenzenesulfonate was activated by Fe_3_O_4_@SiO_2_@PCLH-TFA, and converted to its corresponding enolic form. Then the mentioned enolic form was reacted with activated benzaldehyde with catalyst. This reaction leads to the formation of chalcone intermediate **A**. Then, intermediate **A** undergoes a nucleophilic attack from the enolic form of 4-acetylphenyl-4-methylbenzenesulfonate which resulted in the formation of intermediate **B**. In the next step, ammonia derived from the dissociation of ammonium acetate attacked to intermediate **B** and enamine **C** is produced. After this, through a sequential reaction including, a tautomerization process, intramolecular nucleophilic attack and dehydration, intermediate **C** converted to intermediate **E**. Finally, releasing of molecular H_2_ is facilitated based on a cooperative vinylogous anomeric-based oxidation (CVABO) in intermediate **E** which leads to the formation of the desired molecule after deprotonation of intermediate **F**.

**Figure 8:**
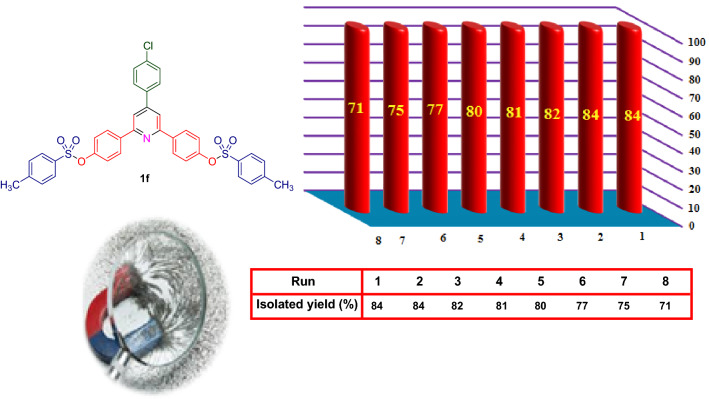
Recovering and reusing test of Fe_3_O_4_@SiO_2_@PCLH-TFA upon the synthesis of **1f**.

Considering the high potential of magnetic catalysts in recovering and reusing processes, we investigated the recovering and reusing of Fe_3_O_4_@SiO_2_@PCLH-TFA in a model reaction for the synthesis of target molecule **1f.** under optimal reaction conditions (Fig. 8). After completing each run of reaction, the catalyst was separated from the mixture of reaction using an external magnet. Then, the separated catalyst was washed several times with ethanol and dried. This process was performed seven times without significant reduction in the reaction efficiency. Finally, the stability of the catalyst was confirmed by comparison of FT‐IR spectra of recovered and fresh catalyst (See ESI).

**Figure 9:**
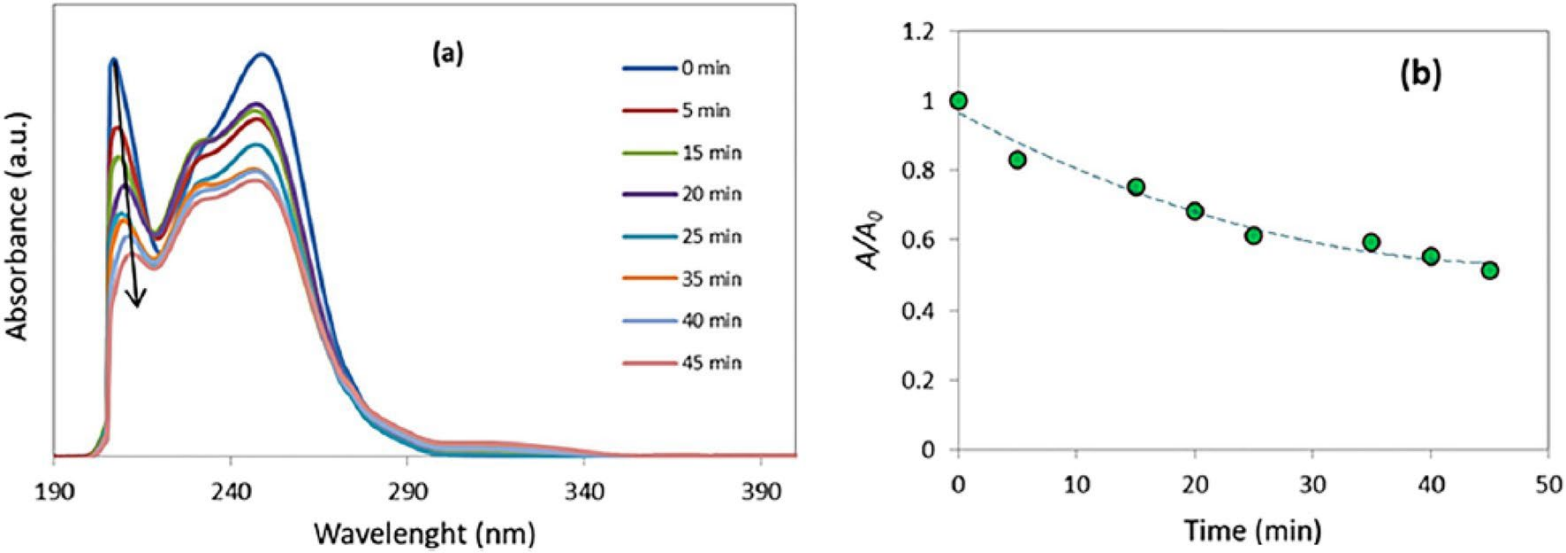
The reaction kinetics by using UV–Visible spectroscopy.

In a separate study, the reaction kinetics was investigated by UV–visible spectroscopy upon the model reaction for the synthesis of molecule 1f. For this purpose, the UV–visible spectrum of reaction components was recorded as a function of time. At different time intervals, a sample of the reaction mixture was dissolved in ethanol and its spectrum was recorded with a UV–visible spectrophotometer. The obtained spectrums at different time intervals were presented in Fig. 9a. The peak centered at 250 nm doesn’t show any regular variation because of the overlapping of reactants, intermediates, and products peaks. But the peak centered at about 210 nm, decreases with time, as shown by an arrow. This peak belongs to reactants since decreases with reaction time. The plot of normalized absorbance (*A*/*A*_0_) at λ_max_ = 210 nm versus time is shown in Fig. 9b. This plot clearly shows non-linear decrease of absorbance versus time. The slope of this plot (rate of reaction) at *t* < 20 min is higher than its slope at *t* > 20 min, indicating higher rate of reaction at the first 20 min of reaction. At a longer time, the rate of reaction decreases.

**Scheme 8:**
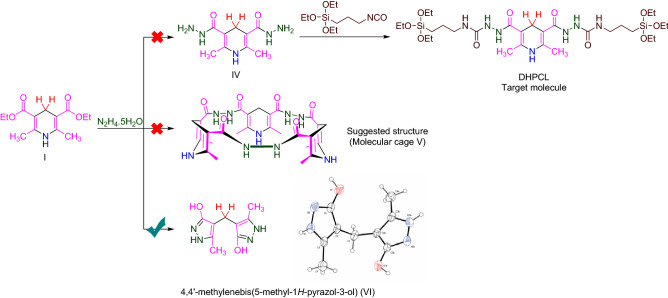
Reaction of the **I** and hydrazine hydrate leads to formation of **VI**.

**Figure 10:**
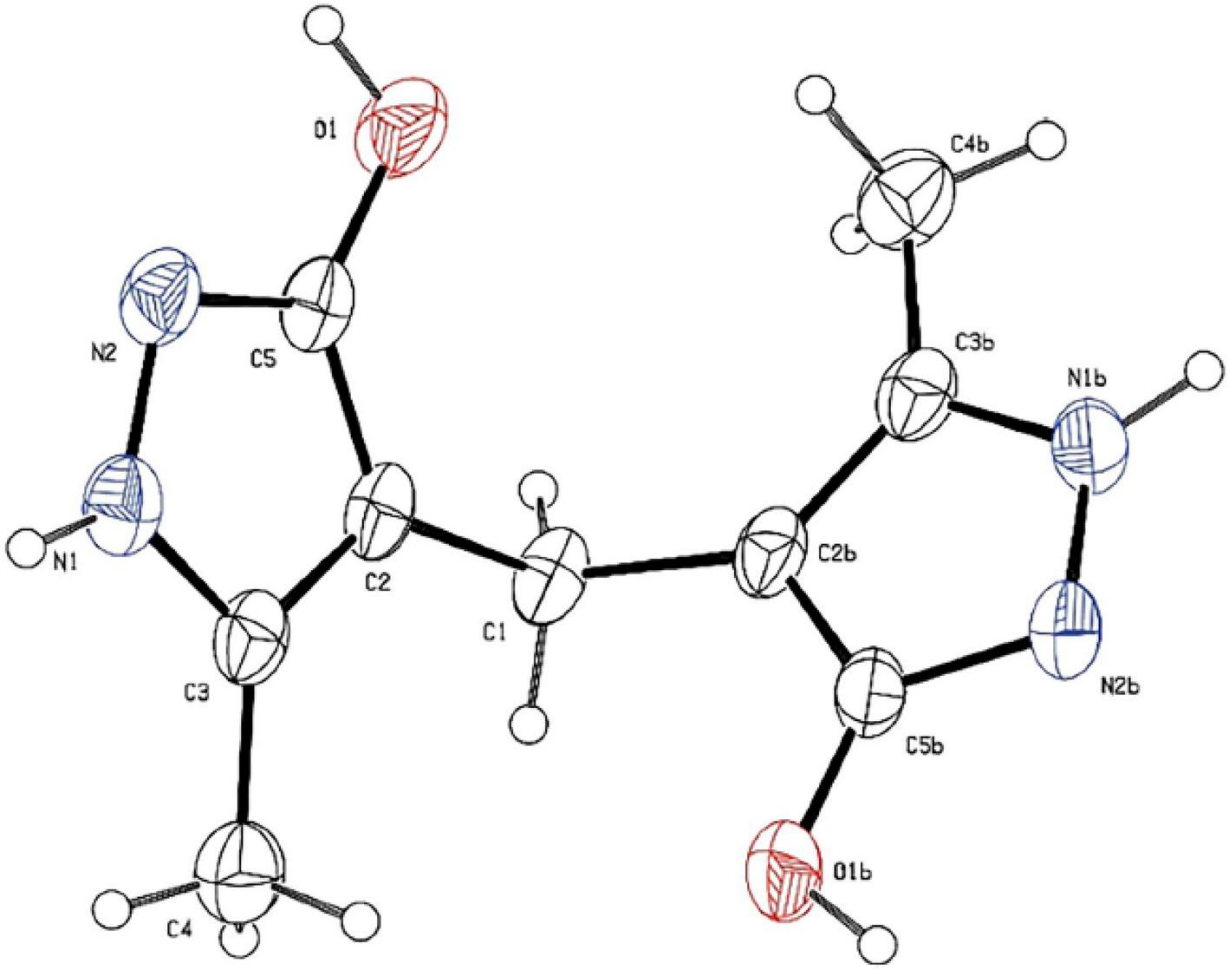
The ORTEP diagram of **VI**. Thermal ellipsoids are at 30% probability level. Disordered DMSO molecule has been omitted for clarity.

**Scheme 9:**
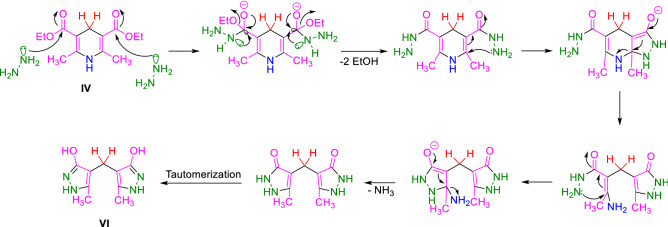
Plausible mechanistic route to VI.

Also, in another investigation and similar to the synthetic protocol of PCL, we tried to use (**I**) directly for the preparation of a new dihydropyridine containing ligand (DHPCL) as a reductant reagent such as biological NADH2/NAD^+^ systems which capable to be heterogeneous. For this goal, (**I**) and hydrazine hydrate subjected to the reaction under refluxing EtOH. Our prediction was the production of molecule (**IV**). The achieved NMR data (see ESI) did not match the expected structure (**IV**). Initial interpretations of the obtained NMR data were apparently in accordance with a molecular cage (**V**). So, with this hypothesis, we applied X-ray crystallography to determine the exact structure. Surprisingly, based on the results of X-ray crystallography, it is revealed that 4,4′-methylenebis(5-methyl-*1H*-pyrazol-3-ol) (**VI**) has been formed (Scheme 8 and Fig. 10)^114,115^. Our literature surveys showed that the molecule (**VI**) had been previously reported^116^. A suggested plausible mechanism for the synthesis of the molecule (**VI**) is depicted in Scheme 9.

## Experimental section

### General

All materials and reagents were purchased from Merck and Sigma-Aldrich companies and were used without further purification. Double distilled water was used in all reactions. ^1^H NMR spectra were recorded on a Bruker spectrometer operating at 300 MHz, and ^13^C NMR spectra were recorded on a Bruker spectrometer operating at 75 MHz. TEM analysis was performed with an instrument model EM10C-100 kV from ZEISS. SEM analysis was performed with SIGMA VP instrument from ZEISS. The instrument of EDX-Mapping analysis is related to Oxford Instruments company. TGA/DTG analysis was performed under nitrogen conditions applying STA-1500 instrument from Rheometric Scientific. The magnetic properties of the catalyst were investigated with VSM analysis that was performed using LBKFB instrument from Meghnatis Daghigh Kavir Company. XRD analysis was performed with X’ Pert Pro instrument from Panalytical. A UV–visible spectrophotometer (PG-Instrument- T80) was applied for the study of reaction kinetics.

#### General procedure for the preparation of 2,6-dimethylpyridine-3,5-dicarbohydrazide

Initially, diethyl 2,6-dimethyl-1,4-dihydropyridine-3,5-dicarboxylate (**I**) was prepared according to the previously reported procedure^117^. Then, (**I**) (20 mmol, 5.06 g) and sodium nitrite (40 mmol, 2.76 g) were dissolved in 70 mL of ethanol/water (5/2, V/V) and heated in 50 °C for 5 min. Then, acetic acid (42 mmol, 2.4 mL) was added dropwise to the reaction mixture and was refluxed for 2 h. After completing the reaction and removal of solvent, the precipitate was washed with water to give diethyl 2,6-dimethylpyridine-3,5-dicarboxylate (**II**) as a white solid in 90% yield. MP (°C) = 72–74^118^. In the next step, hydrazine hydrate (40 mmol, 4.88 g) was added to (**II**) (10 mmol, 2.51 g) and was heated for 6 h at 80 °C. After removal of water, the remained precipitate was washed with ethanol several times to give pure 2,6-dimethylpyridine-3,5-dicarbohydrazide (**III**) as a white solid.

#### General procedure for the synthesis of PCL

PCL was synthesized by the reaction of triethoxy(3‐isocyanatopropyl) silane (5 mmol, 1.237 g) and **III** (12 mmol, 2.68 g) under neat conditions at room temperature for 24 h. Afterward, the obtained white precipitate was washed with the mixture of ethyl acetate/chloroform (8 × 2 ml). The remained white precipitate was dried and characterized by FT-IR, ^1^H NMR and ^13^C NMR.

#### General procedure for the synthesis of Fe_3_O_4_@SiO_2_@PCLH-TFA

At first, Fe_3_O_4_ nanoparticles were prepared by the same method as reported in previously reported procedure^119^. Then, 2 g of Fe_3_O_4_ and 3 ml ammonia and 4 ml TEOS was added to 200 ml of EtOH/H_2_O (4/1, V/V) and stirred at room temperature for 24 h. Then, nanoparticles of Fe_3_O_4_@SiO_2_ were separated by an external magnet and were washed several times with water and ethanol and finally air dried. In the next step, the prepared Fe_3_O_4_@SiO_2_ (1 g) was functionalized by the reaction with ligand PCL (2 mmol, 1.38 g) under refluxing toluene at 110 °C for 12 h to give Fe_3_O_4_@SiO_2_@PCL. At the final step, Fe_3_O_4_@SiO_2_@PCL was treated with trifluoroacetic acid (2 mmol, 0.228 g) in toluene at room temperature for 6 h and after that, the resulted Fe_3_O_4_@SiO_2_@PCLH-TFA was washed with *n-*hexane (3 × 30 ml) and air dried.

#### General procedure for the synthesis of new library of triarylpyridines bearing sulfonate and sulfonamide moieties in the presence of Fe_3_O_4_@SiO_2_@PCLH-TFA

To a mixture of aldehyde derivatives (1 mmol), methyl ketones with sulfonate and sulfonamide moieties^120,121^ (2 mmol) and ammonium acetate (1.5 mmol, 0.115 g), 10 mg of Fe_3_O_4_@SiO_2_@PCLH-TFA as catalyst were added. Then, the reaction mixture was stirred under solvent-free conditions at 110 °C for requisite times (Table 3). The progress of the reaction was inspected by TLC techniques (*n*-hexane/ethylacetate as eluent). After completion of each reaction, the reaction mixture was dissolved in hot ethanol and the insoluble nanomagnetic catalyst was isolated from the reaction mixture by using an external magnet. Then, target molecules were purified by TLC plate with *n*-hexane/ethyl acetate as eluent.

### Spectral data

#### 2,6-Dimethylpyridine-3,5-dicarbohydrazide (III)

M.p. = 230–232 °C, FT‐IR (KBr, ν, cm^−1^): 3302, 3202, 3067, 1640, 1594, 1185. ^1^H NMR (301 MHz, DMSO) δppm 9.54 (s, 2H, NH), 7.65 (s, 1H, Aromatic), 4.57 (s, 4H, NH_2_), 2.54 (s, 6H, CH_3_). ^13^C NMR (76 MHz, DMSO) δ 167.2, 156.6, 135.2, 127.6, 23.0.

#### 2,2′-(2,6-Dimethylpyridine-3,5-dicarbonyl)bis(*N*-(3-(triethoxysilyl)propyl)hydrazine-1-carboxamide) (PCL)

M.p. = 170–171 °C, FT‐IR (KBr, ν, cm^−1^): 3304, 2974, 1701, 1650, 1567, 1083. ^1^H NMR (301 MHz, DMSO) δppm 9.89 (s, 2H, NH), 7.94–7.91 (m, 3H, Aromatic and NH), 6.49 (s, 2H, NH), 3.77 (q, 12H, *J* = 6 Hz, CH_2_), 3.03 (q, 4H, *J* = 6 Hz, CH_2_), 2.58 (s, 6H, CH_3_), 1.48 (q, 4H, *J* = 6 Hz, CH_2_), 1.17 (t, 18H, *J* = 6 Hz, CH_3_), 0.56 (t, 4H, *J* = 9 Hz, CH_2_). ^13^C NMR (76 MHz, DMSO) δ 167.7, 158.5, 157.2, 135.6, 127.1, 58.2, 42.5, 23.9, 23.1, 18.7, 7.7.

#### 4-Acetylphenyl benzenesulfonate

M.p. = 60–62 °C, FT‐IR (KBr, ν, cm^−1^): 3063, 1692, 1680, 1593, 1352, 1201. ^1^H NMR (301 MHz, DMSO) δ 8.00–7.97 (m, 2H), 7.93 -7.90 (m, 2H), 7.85–7.82 (m, 1H), 7.72–7.69 (m, 2H), 7.22–7.19 (m, 2H), 2.56 (s, 3H).

#### 4-Acetylphenyl-4-methylbenzenesulfonate

M.p. = 70–72 °C, FT‐IR (KBr, ν, cm^−1^): 6072, 2918, 1682, 1596, 1379, 1176. ^1^H NMR (301 MHz, DMSO) δ 8.00–7.95 (m, 2H), 7.79 (d, *J* = 9 Hz, 2H), 7.47 (d, *J* = 9 Hz, 2H), 7.22–7.17 (m, 2H), 2.55 (s, 3H), 2.40 (s, 3H). ^13^C NMR (76 MHz, DMSO) δ 197.1, 152.7, 146.5, 136.0, 131.7, 130.8, 130.7, 128.9, 122.7, 27.1, 21.6.

#### (4-Phenylpyridine-2,6-diyl)bis(4,1-phenylene)bis(4-methylbenzenesulfonate) (1a)

M.p. = 148–150 °C, FT‐IR (KBr, ν, cm^−1^): 3068, 1600, 1499, 1453, 1179. ^1^H NMR (301 MHz, DMSO) δppm 8.34 (d, 4H, *J* = 9 Hz), 8.20 (s, 2H), 8.03 (dd, *J* = 8.0, 3 Hz, 2H), 7.82 (d, *J* = 9 Hz, 4H), 7.57–7.49 (m, 6H), 7.19 (d, *J* = 9 Hz, 4H), 2.44 (s, 6H, CH_3_). ^13^C NMR (76 MHz, DMSO) δ 155.6, 150.3, 150.2, 146.4, 138.1, 137.79, 131.9, 130.8, 129.9, 129.5, 129.1, 128.8, 127.9, 122.8, 117.5, 21.7.

#### (4-(4-Hydroxyphenyl)pyridine-2,6-diyl)bis(4,1-phenylene)bis(4-methylbenzenesulfonate) (1b)

M.p. = 178–179 °C, FT‐IR (KBr, ν, cm^−1^): 3471, 3058, 1603, 1502, 1372, 1176. ^1^H NMR (301 MHz, DMSO) δppm 8.35 (d, 4H, *J* = 9 Hz), 8.24 (s, 2H), 8.05 (q, *J* = 9 Hz, 2H), 7.83–7.76 (m, 6H), 7.53–7.50 (m, 6H), 7.20–7.17 (m, 4H), 2.45 (s, 6H, CH_3_). ^13^C NMR (76 MHz, DMSO) δ 155.7, 150.4, 146.4, 138.0, 137.0, 132.4, 131.9, 130.8, 130.0, 129.2, 128.8, 128.7, 123.1, 122.8, 117.3, 21.7.

#### (4-(4-Methoxyphenyl)pyridine-2,6-diyl)bis(4,1-phenylene)bis(4-methylbenzenesulfonate) (1c)

M.p. = 290–291 °C, FT‐IR (KBr, ν, cm^−1^): 2923, 1603, 1501, 1368, 1153, ^1^H NMR (301 MHz, DMSO) δ 8.32 (d, *J* = 9 Hz, 4H), 8.15 (s, 2H), 8.02 (d, *J* = 9 Hz, 2H), 7.82 (d, *J* = 9 Hz, 4H), 7.51 (d, *J* = 9 Hz, 4H), 7.18 (d, *J* = 9 Hz, 4H), 7.11 (d, *J* = 9 Hz, 2H), 3.86 (s, 3H, OCH_3_), 2.44 (s, 6H). ^13^C NMR (76 MHz, DMSO) δ 161.0, 155.5, 150.3, 149.7, 146.4, 138.2, 131.9, 130.8, 129.9, 129.2, 129.1, 128.8, 122.7, 116.7, 114.9, 55.8, 21.7.

#### (4-(2-Methoxyphenyl)pyridine-2,6-diyl)bis(4,1-phenylene)bis(4-methylbenzenesulfonate) (1d)

M.p. = 178–179 °C, FT‐IR (KBr, ν, cm^−1^): 3061, 2926, 1601, 1500, 1372, 1152. ^1^H NMR (301 MHz, DMSO) δ 8.24 (d, *J* = 9 Hz, 4H), 7.99 (s, 2H), 7.81 (d, *J* = 9 Hz, 4H), 7.55 (dd, *J* = 7.5, 1.7 Hz, 1H), 7.49 (m, 5H), 7.21–7.14 (m, 6H), 3.82 (s, 3H, OCH_3_), 2.42 (s, 6H). ^13^C NMR (76 MHz, DMSO) δ 156.9, 154.8, 150.2, 148.8, 146.4, 138.2, 131.9, 131.0, 130.8, 129.0, 128.7, 127.5, 122.8, 121.4, 120.3, 112.4, 56.2, 21.6. ESI–MS (m/z) = calcd. for C_38_H_31_NO_7_S_2_ (M^+^) 677.15, found: 678.

#### (4-(3,4-Dimethoxyphenyl)pyridine-2,6-diyl)bis(4,1-phenylene)bis(4-methylbenzenesulfonate) (1e)

M.p. = 113–114 °C, FT‐IR (KBr, ν, cm^−1^): 3064, 2923, 1600, 1501, 1370, 1263, 1152. ^1^H NMR (301 MHz, DMSO) δ 8.33 (d, *J* = 9 Hz, 4H), 8.16 (s, 2H), 7.82 (d, *J* = 9 Hz, 4H), 7.62–7.55 (m, 2H), 7.50 (d, *J* = 9 Hz, 4H), 7.19 (d, *J* = 9 Hz, 4H), 7.11 (d, *J* = 9 Hz, 1H), 3.93 (s, 3H, OCH_3_), 3.85 (s, 3H, OCH_3_), 2.43 (s, 6H). ^13^C NMR (76 MHz, DMSO) δ 155.4, 150.6, 150.3, 150.2, 149.7, 146.4, 138.2, 131.9, 130.8, 130.3, 129.1, 128.8, 122.7, 120.5, 117.0, 112.4, 111.3, 56.4, 56.1, 21.7. ESI–MS (m/z) = calcd. for C_39_H_33_NO_8_S_2_ (M^+^) 707.16, found: 708.

#### (4-(4-Chlorophenyl)pyridine-2,6-diyl)bis(4,1-phenylene)bis(4-methylbenzenesulfonate) (1f.)

M.p. = 251–253 °C, FT‐IR (KBr, ν, cm^−1^): 3233, 1637, 1615, 1372, 1152. ^1^H NMR (301 MHz, DMSO) δ 8.35 (d, *J* = 9 Hz, 4H), 8.24 (s, 2H), 8.15–8.08 (m, 2H), 7.82 (d, *J* = 9 Hz, 4H), 7.64 (d, *J* = 9 Hz, 2H), 7.52 (d, *J* = 9 Hz, 4H), 7.20 (d, *J* = 9 Hz, 4H), 2.45 (s, 6H, CH_3_). ^13^C NMR (76 MHz, DMSO) δ 162.8, 155.7, 150.3, 146.4, 138.0, 136.8, 136.6, 131.9, 130.8, 129.7, 129.5, 129.2, 128.8, 122.8, 117.4, 21.7.

#### (4-(4-Bromophenyl)pyridine-2,6-diyl)bis(4,1-phenylene)bis(4-methylbenzenesulfonate) (1g)

M.p. = 227–229 °C, FT‐IR (KBr, ν, cm^−1^): 3114, 1602, 1497, 1368, 1152. ^1^H NMR (301 MHz, DMSO) δ 8.32 (d, *J* = 9 Hz, 4H), 8.13 (s, 2H), 7.92 (d, *J* = 9 Hz, 2H), 7.82 (d, *J* = 9 Hz, 4H), 7.51 (d, *J* = 6 Hz, 4H), 7.18 (d, *J* = 9 Hz, 4H), 6.95 (d, *J* = 9 Hz, 2H), 2.45 (s, 6H, CH_3_). ^13^C NMR (76 MHz, DMSO) δ 159.6, 155.4, 150.2, 150.1, 146.4, 138.3, 131.9, 130.8, 129.2, 129.1, 128.8, 128.1, 122.7, 116.5, 116.3, 21.7.

#### (4-(4-Isopropylphenyl)pyridine-2,6-diyl)bis(4,1-phenylene)dibenzenesulfonate (2a)

M.p. = 145–148 °C, FT‐IR (KBr, ν, cm^−1^): 3069, 2960, 1603, 1503, 1367, 1199. ^1^H NMR (301 MHz, DMSO) δ 8.33 (d, *J* = 9 Hz, 4H), 8.17 (s, 2H), 7.95 (d, *J* = 9 Hz, 5H), 7.86–7.83 (m, 1H), 7.74–7.69 (m, 4H), 7.43 (d, *J* = 9 Hz, 2H), 7.19 (d, *J* = 8.9 Hz, 4H), 2.98 (hept, *J* = 6 Hz, 1H, CH), 1.26 (d, *J* = 6.9 Hz, 6H, CH_3_). ^13^C NMR (76 MHz, DMSO) δ 155.5, 150.4, 150.2, 150.2, 138.2, 135.6, 135.4, 134.8, 130.4, 129.1, 128.7, 127.7, 127.5, 122.8, 117.3, 33.7, 24.2. ESI–MS (m/z) = calcd. for C_38_H_31_NO_6_S_2_ (M^+^) 661.15, found: 662.

#### (4-(4-Methoxyphenyl)pyridine-2,6-diyl)bis(4,1-phenylene)dibenzenesulfonate (2b)

M.p. = 111–113 °C, FT‐IR (KBr, ν, cm^−1^): 3067, 2923, 1599, 1501, 1373, 1178. ^1^H NMR (301 MHz, DMSO) δ 8.33 (d, *J* = 9 Hz, 4H), 8.17 (s, 2H), 8.03 (d, *J* = 9 Hz, 2H), 7.96–7.93 (m, 4H), 7.89–7.83 (m, 2H), 7.74–7.69 (m, 4H), 7.19 (d, *J* = 9 Hz, 4H), 7.11 (d, *J* = 9 Hz, 2H), 3.86 (s, 3H, OCH_3_). ^13^C NMR (76 MHz, DMSO) δ 161.0, 155.5, 150.2, 149.8, 138.3, 135.6, 134.8, 130.4, 129.9, 129.2, 129.1, 128.8, 122.7, 116.8, 114.9, 55.8.

#### (4-(4-Chlorophenyl)pyridine-2,6-diyl)bis(4,1-phenylene)dibenzenesulfonate (2c)

M.p. = 186–189 °C, FT‐IR (KBr, ν, cm^−1^): 3064, 2926, 1606, 1546, 1494, 1371, 1152. ^1^H NMR (301 MHz, DMSO) δ 8.35 (d, *J* = 9 Hz, 4H), 8.23 (s, 2H), 8.12–8.08 (m, 2H), 7.96–7.93 (m, 4H), 7.91–7.84 (m, 4H), 7.74–7.69 (m, 4H), 7.63 (s, 1H), 7.22–7.18 (m, 4H). ^13^C NMR (76 MHz, DMSO) δ 155.7, 150.3, 148.9, 138.1, 136.6, 135.6, 134.8, 131.0, 130.8, 130.4, 129.7, 129.5, 129.2, 128.8, 117.4.

#### (4-(2-Chlorophenyl)pyridine-2,6-diyl)bis(4,1-phenylene)dibenzenesulfonate (2d)

M.p. = 125–127 °C, FT‐IR (KBr, ν, cm^−1^): 3061, 2923, 1603, 1504, 1448, 1371, 1152. ^1^H NMR (301 MHz, DMSO) δ 8.29 (d, *J* = 9 Hz, 4H), 8.01 (s, 2H), 7.95–7.92 (m, 4H), 7.85 (dt, *J* = 9, 1.7 Hz, 2H), 7.73 (d, *J* = 9 Hz, 4H), 7.68–7.65 (m, 2H), 7.55–7.51 (m, 2H), 7.19 (d, *J* = 9 Hz, 4H). ^13^C NMR (76 MHz, DMSO) δ 155.0, 150.3, 149.3, 137.9, 137.8, 135.6, 134.8, 131.9, 131.7, 130.9, 130.5, 130.4, 129.1, 128.7, 128.2, 122.9, 120.4.

#### *N,N*′-((4-Phenylpyridine-2,6-diyl)bis(4,1-phenylene))bis(4-methylbenzenesulfonamide) (3a)

M.p. = 114–116 °C, FT‐IR (KBr, ν, cm^−1^): 3259, 2923, 1694, 1603, 1598, 1514, 1331, 1160. ^1^H NMR (301 MHz, DMSO) δ 10.47 (s, 2H, NH), 8.19 (d, *J* = 9 Hz, 4H), 8.03 (s, 2H), 7.96 (d, *J* = 6 Hz, 2H), 7.76 (d, *J* = 9 Hz, 4H), 7.58–7.50 (m, 2H), 7.37–7.28 (m, 8H), 2.31 (s, 6H, CH_3_). ^13^C NMR (76 MHz, DMSO) δ 162.8, 156.2, 149.9, 143.9, 139.4, 138.2, 137.2, 134.6, 130.2, 129.5, 128.3, 127.7, 127.3, 127.2, 120.0, 116.1, 21.4.

#### *N,N*′-((4-(4-Chlorophenyl)pyridine-2,6-diyl)bis(4,1-phenylene))bis(4-methylbenzenesulfonamide) (3b)

M.p. = 127–129 °C, FT‐IR (KBr, ν, cm^−1^): 3256, 2929, 1600, 1514, 1332, 1160. ^1^H NMR (301 MHz, DMSO) δ 10.53 (s, 2H, NH), 8.18 (d, *J* = 9 Hz, 4H), 8.05–8.02 (m, 4H), 7.74 (d, *J* = 9 Hz, 4H), 7.61 (d, *J* = 6 Hz, 2H), 7.37 (d, *J* = 6 Hz, 4H), 7.27 (d, *J* = 9 Hz, 4H), 2.33 (s, 6H, CH_3_). ^13^C NMR (76 MHz, DMSO) δ 156.2, 148.5, 143.9, 139.4, 137.2, 137.0, 134.7, 134.5, 130.2, 129.6, 129.5, 128.3, 127.3, 119.9, 116.0, 21.4. ESI–MS (m/z) = calcd. for C_37_H_30_ClN_3_O_4_S_2_ (M^+^) 680.23, found: 680.

#### *N,N*′-((4-(4-Bromophenyl)pyridine-2,6-diyl)bis(4,1-phenylene))bis(4-methylbenzenesulfonamide) (3c)

M.p. = 143–144 °C, FT‐IR (KBr, ν, cm^−1^): 3250, 2920, 1600, 1514, 1322, 1160. ^1^H NMR (301 MHz, DMSO) δ 10.54 (s, 2H, NH), 8.18 (d, *J* = 9 Hz, 4H), 8.03 (s, 2H), 7.97–7.92 (m, 2H), 7.75–7.72 (m, 6H), 7.36 (d, *J* = 6 Hz, 4H), 7.28 (d, *J* = 9 Hz, 4H), 2.32 (s, 6H, CH_3_). ^13^C NMR (76 MHz, DMSO) δ 155.6, 150.3, 148.7, 146.4, 137.9, 136.8, 131.9, 130.8, 129.6, 129.1, 128.8, 123.1, 122.8, 117.5, 22.2.

#### (1,4-Phenylenebis(pyridine-4,2,6-triyl))tetrakis(benzene-4,1-diyl)tetrakis(4-methylbenzenesulfonate) (1 h).

M.p. ≥ 300 °C, FT‐IR (KBr, ν, cm^−1^): 3058, 2926, 1651, 1600, 1499, 1371, 1152. ^1^H NMR (301 MHz, DMSO) δ 8.4–8.25 (m, 11H), 7.84–7.76 (m, 10H), 7.54–7.48 (m, 10H), 7.31 (d, *J* = 9 Hz, 2H), 7.23–7.17 (m, 5H), 6.99 (d, *J* = 9 Hz, 2H), 2.45 (m, 12H, CH_3_). ^13^C NMR (76 MHz, DMSO) δ 168.8, 155.7, 150.4, 148.1, 146.4, 146.2, 144.5, 138.1, 132.2, 131.9, 130.8, 130.7, 129.9, 129.2, 128.8, 128.6, 128.0, 122.8, 122.2, 117.5, 21.7. ESI–MS (m/z) = calcd. for C_68_H_52_N_2_O_12_S_4_ (M^+^) 1217.41, found: 1218.

#### *N,N*′*,N'',N'''*-((1,4-Phenylenebis(pyridine-4,2,6-triyl))tetrakis(benzene-4,1-diyl))tetrakis(4-methylbenzenesulfonamide) (3d)

M.p. ≥ 300 °C, FT‐IR (KBr, ν, cm^−1^): 3458, 2925, 1605, 1545, 1368, 1179. ^1^H NMR (301 MHz, DMSO) δ 10.56 (s, 4H, NH), 8.14 (d, *J* = 9 Hz, 4H), 7.95 (s, 2H), 7.88–7.83 (m, 4H), 7.76–7.71 (m, 10H), 7.40–7.35 (m, 10H), 7.27–7.21 (m, 10H), 2.35–2.33 (m, 12H, CH_3_). ^13^C NMR (76 MHz, DMSO) δ 156.1, 148.9, 144.1, 143.9, 143.0, 139.4, 139.3, 137.2, 137.1, 137.0, 134.6, 133.2, 132.3, 130.3, 130.3, 130.2, 128.6, 128.3, 127.2, 127.2, 120.0, 119.9, 118.3, 115.6, 21.4.

## Conclusion

In this work, Fe_3_O_4_@SiO_2_@PCLH-TFA as a novel magnetic nanoparticle with pyridinium bridges was designed, synthesized and characterized with FT-IR, EDX, XRD, SEM, TEM, TG/DTG and VSM. Then, Fe_3_O_4_@SiO_2_@PCLH-TFA was applied in the multicomponent synthesis of a new library of triarylpyridines bearing sulfonate and sulfonamide moieties via a cooperative vinylogous anomeric-based oxidation. According to the obtained results, the reactions were performed under mild conditions and short reaction time, and the synthesis of molecules show good yields. Furthermore, Fe_3_O_4_@SiO_2_@PCLH-TFA show elegant performance in recovering and reusing test.

## Supplementary Information

Supplementary Information.
