# Supplementary material for: Synthesis of triarylpyridines with sulfonate and sulfonamide moieties via a cooperative vinylogous anomeric-based oxidation
Source: Sci Rep. 2021 Aug 19;11:16846. doi: 10.1038/s41598-021-95830-w (PMC8377147; doi:10.1038/s41598-021-95830-w)

Synthesis of triarylpyridines with sulfonate and sulfonamide moieties via a cooperative vinylogous anomeric-based oxidation

**Morteza Torabi,^a^ Mohammad Ali Zolfigol,*^a^ Meysam Yarie,^a^ Behrouz Notash,^b^ Saeid Azizian,^c^ Mina Mirzaei Azandaryani^c^**

^a^Department of Organic Chemistry, Faculty of Chemistry, Bu‐Ali Sina University, Hamedan 6517838683, Iran, Email: zolfi@basu.ac.ir and mzolfigol@yahoo.com

^b^Department of Inorganic Chemistry and Catalysis, Shahid Beheshti University, Evin, Tehran, Iran.

^c^Department of Physical Chemistry, Faculty of Chemistry, Bu-Ali Sina University, Hamedan 6517838683, Iran.

**Content**

1: FT-IR spectrum of diethyl 2, 6-dimethyl-1,4-dihydropyridine-3,5-dicarboxylate

2: FT-IR spectrum of diethyl 2,6-dimethylpyridine-3,5-dicarboxylate

3: FT-IR spectrum of 2,6-dimethylpyridine-3,5-dicarbohydrazide

4: ^1^H NMR spectrum of 2,6-dimethylpyridine-3,5-dicarbohydrazide

5: ^13^C NMR spectrum of 2,6-dimethylpyridine-3,5-dicarbohydrazide

6: FT-IR spectrum of 2,2'-(2,6-dimethylpyridine-3,5-dicarbonyl)bis(*N*-(3-(triethoxysilyl)propyl)hydrazine-1-carboxamide) (PCL)

7: ^1^H NMR spectrum of 2,2'-(2,6-dimethylpyridine-3,5-dicarbonyl)bis(*N*-(3-(triethoxysilyl)propyl)hydrazine-1-carboxamide) (PCL)

8: ^13^C NMR spectrum of 2,2'-(2,6-dimethylpyridine-3,5-dicarbonyl)bis(*N*-(3-(triethoxysilyl)propyl)hydrazine-1-carboxamide) (PCL)

9: FT-IR spectrum of 4-acetylphenyl benzenesulfonate

10: ^1^H NMR spectrum of 4-acetylphenyl benzenesulfonate

11: FT-IR spectrum of 4-acetylphenyl 4-methylbenzenesulfonate

12: ^1^H NMR spectrum of 4-acetylphenyl 4-methylbenzenesulfonate

13: ^13^C NMR spectrum of 4-acetylphenyl 4-methylbenzenesulfonate

14: FT-IR spectrum of (4-phenylpyridine-2,6-diyl)bis(4,1-phenylene) bis(4-methylbenzenesulfonate) (1a)

15: ^1^H NMR spectrum of (4-phenylpyridine-2,6-diyl)bis(4,1-phenylene) bis(4-methylbenzenesulfonate) (1a)

16: ^13^C NMR spectrum of (4-phenylpyridine-2,6-diyl)bis(4,1-phenylene) bis(4-methylbenzenesulfonate) (1a)

17: FT-IR spectrum of (4-(4-hydroxyphenyl)pyridine-2,6-diyl)bis(4,1-phenylene) bis(4-methylbenzenesulfonate) (1b)

18: ^1^H NMR spectrum of (4-(4-hydroxyphenyl)pyridine-2,6-diyl)bis(4,1-phenylene) bis(4-methylbenzenesulfonate) (1b)

19: ^13^C NMR spectrum of (4-(4-hydroxyphenyl)pyridine-2,6-diyl)bis(4,1-phenylene) bis(4-methylbenzenesulfonate) (1b)

20: FT-IR spectrum of (4-(4-methoxyphenyl)pyridine-2,6-diyl)bis(4,1-phenylene) bis(4-methylbenzenesulfonate) (1c)

21: ^1^H NMR spectrum of (4-(4-methoxyphenyl)pyridine-2,6-diyl)bis(4,1-phenylene) bis(4-methylbenzenesulfonate) (1c)

22: ^13^C NMR spectrum of (4-(4-methoxyphenyl)pyridine-2,6-diyl)bis(4,1-phenylene) bis(4-methylbenzenesulfonate) (1c)

23: FT-IR spectrum of (4-(2-methoxyphenyl)pyridine-2,6-diyl)bis(4,1-phenylene) bis(4-methylbenzenesulfonate) (1d)

24: ^1^H NMR spectrum of (4-(2-methoxyphenyl)pyridine-2,6-diyl)bis(4,1-phenylene) bis(4-methylbenzenesulfonate) (1d)

25: ^13^C NMR spectrum of (4-(2-methoxyphenyl)pyridine-2,6-diyl)bis(4,1-phenylene) bis(4-methylbenzenesulfonate) (1d)

26: Mass spectrum of (4-(2-methoxyphenyl)pyridine-2,6-diyl)bis(4,1-phenylene) bis(4-methylbenzenesulfonate) (1d)

27: FT-IR spectrum of (4-(3,4-dimethoxyphenyl)pyridine-2,6-diyl)bis(4,1-phenylene) bis(4-methylbenzenesulfonate) (1e)

28: ^1^H NMR spectrum of (4-(3,4-dimethoxyphenyl)pyridine-2,6-diyl)bis(4,1-phenylene) bis(4-methylbenzenesulfonate) (1e)

29: ^13^C NMR spectrum of (4-(3,4-dimethoxyphenyl)pyridine-2,6-diyl)bis(4,1-phenylene) bis(4-methylbenzenesulfonate) (1e)

30: Mass spectrum of (4-(3,4-dimethoxyphenyl)pyridine-2,6-diyl)bis(4,1-phenylene) bis(4-methylbenzenesulfonate) (1e)

31: FT-IR spectrum of (4-(4-chlorophenyl)pyridine-2,6-diyl)bis(4,1-phenylene) bis(4-methylbenzenesulfonate) (1f)

32: ^1^H NMR spectrum of (4-(4-chlorophenyl)pyridine-2,6-diyl)bis(4,1-phenylene) bis(4-methylbenzenesulfonate) (1f)

33: ^13^C NMR spectrum of (4-(4-chlorophenyl)pyridine-2,6-diyl)bis(4,1-phenylene) bis(4-methylbenzenesulfonate) (1f)

34: Mass spectrum of (4-(4-chlorophenyl)pyridine-2,6-diyl)bis(4,1-phenylene) bis(4-methylbenzenesulfonate) (1f)

35: FT-IR spectrum of (4-(4-bromophenyl)pyridine-2,6-diyl)bis(4,1-phenylene) bis(4-methylbenzenesulfonate) (1g)

36: ^1^H NMR spectrum of (4-(4-bromophenyl)pyridine-2,6-diyl)bis(4,1-phenylene) bis(4-methylbenzenesulfonate) (1g)

37: ^13^C NMR spectrum of (4-(4-bromophenyl)pyridine-2,6-diyl)bis(4,1-phenylene) bis(4-methylbenzenesulfonate) (1g)

38: FT-IR spectrum of (4-(4-isopropylphenyl)pyridine-2,6-diyl)bis(4,1-phenylene) dibenzenesulfonate (2a)

39: ^1^H NMR spectrum of (4-(4-isopropylphenyl)pyridine-2,6-diyl)bis(4,1-phenylene) dibenzenesulfonate (2a)

40: ^13^C NMR spectrum of (4-(4-isopropylphenyl)pyridine-2,6-diyl)bis(4,1-phenylene) dibenzenesulfonate (2a)

41: Mass spectrum of (4-(4-isopropylphenyl)pyridine-2,6-diyl)bis(4,1-phenylene) dibenzenesulfonate (2b)

42: FT-IR spectrum of (4-(4-methoxyphenyl)pyridine-2,6-diyl)bis(4,1-phenylene) dibenzenesulfonate (2b)

43: ^1^H NMR spectrum of (4-(4-methoxyphenyl)pyridine-2,6-diyl)bis(4,1-phenylene) dibenzenesulfonate (2b)

44: ^13^C NMR spectrum of (4-(4-methoxyphenyl)pyridine-2,6-diyl)bis(4,1-phenylene) dibenzenesulfonate (2b)

45: FT-IR spectrum of (4-(4-chlorophenyl)pyridine-2,6-diyl)bis(4,1-phenylene) dibenzenesulfonate (2c)

46: ^1^H NMR spectrum of (4-(4-chlorophenyl)pyridine-2,6-diyl)bis(4,1-phenylene) dibenzenesulfonate (2c)

47: ^13^C NMR spectrum of (4-(4-chlorophenyl)pyridine-2,6-diyl)bis(4,1-phenylene) dibenzenesulfonate (2c)

48: FT-IR spectrum of (4-(2-chlorophenyl)pyridine-2,6-diyl)bis(4,1-phenylene) dibenzenesulfonate (2d)

49: ^1^H NMR spectrum of (4-(2-chlorophenyl)pyridine-2,6-diyl)bis(4,1-phenylene) dibenzenesulfonate (2d)

50: ^13^C NMR spectrum of (4-(2-chlorophenyl)pyridine-2,6-diyl)bis(4,1-phenylene) dibenzenesulfonate (2d)

51: FT-IR spectrum of *N*,*N'*-((4-phenylpyridine-2,6-diyl)bis(4,1-phenylene))bis(4-methylbenzenesulfonamide) (3a)

52: ^1^H NMR spectrum of *N*,*N'*-((4-phenylpyridine-2,6-diyl)bis(4,1-phenylene))bis(4-methylbenzenesulfonamide) (3a)

53: ^13^C NMR spectrum of *N*,*N'*-((4-phenylpyridine-2,6-diyl)bis(4,1-phenylene))bis(4-methylbenzenesulfonamide) (3a)

54: FT-IR spectrum of *N*,*N'*-((4-(4-chlorophenyl)pyridine-2,6-diyl)bis(4,1-phenylene))bis(4-methylbenzenesulfonamide) (3b)

55: ^1^H NMR spectrum of *N*,*N'*-((4-(4-chlorophenyl)pyridine-2,6-diyl)bis(4,1-phenylene))bis(4-methylbenzenesulfonamide) (3b)

56: ^13^C NMR spectrum of *N*,*N'*-((4-(4-chlorophenyl)pyridine-2,6-diyl)bis(4,1-phenylene))bis(4-methylbenzenesulfonamide) (3b)

57: FT-IR spectrum of *N*,*N'*-((4-(4-bromophenyl)pyridine-2,6-diyl)bis(4,1-phenylene))bis(4-methylbenzenesulfonamide) (3c)

58: ^1^H NMR spectrum of *N*,*N'*-((4-(4-bromophenyl)pyridine-2,6-diyl)bis(4,1-phenylene))bis(4-methylbenzenesulfonamide) (3c)

59: ^13^C NMR spectrum of *N*,*N'*-((4-(4-bromophenyl)pyridine-2,6-diyl)bis(4,1-phenylene))bis(4-methylbenzenesulfonamide) (3c)

60: FT-IR spectrum of (1,4-phenylenebis(pyridine-4,2,6-triyl))tetrakis(benzene-4,1-diyl) tetrakis(4-methylbenzenesulfonate) (1i)

61: ^1^H NMR spectrum of (1,4-phenylenebis(pyridine-4,2,6-triyl))tetrakis(benzene-4,1-diyl) tetrakis(4-methylbenzenesulfonate) (1i)

62: ^13^C NMR spectrum of (1,4-phenylenebis(pyridine-4,2,6-triyl))tetrakis(benzene-4,1-diyl) tetrakis(4-methylbenzenesulfonate) (1i)

63: Mass spectrum of (1,4-phenylenebis(pyridine-4,2,6-triyl))tetrakis(benzene-4,1-diyl) tetrakis(4-methylbenzenesulfonate) (1i)

64: FT-IR spectrum of *N,N',N'',N'''*-((1,4-phenylenebis(pyridine-4,2,6-triyl))tetrakis(benzene-4,1-diyl))tetrakis(4-methylbenzenesulfonamide) (3d)

65: ^1^H NMR spectrum of *N,N',N'',N'''*-((1,4-phenylenebis(pyridine-4,2,6-triyl))tetrakis(benzene-4,1-diyl))tetrakis(4-methylbenzenesulfonamide) (3d)

66: ^13^C NMR spectrum of *N,N',N'',N'''*-((1,4-phenylenebis(pyridine-4,2,6-triyl))tetrakis(benzene-4,1-diyl))tetrakis(4-methylbenzenesulfonamide) (3d)

67: ^1^H NMR spectrum of 4,4'-methylenebis(5-methyl-1H-pyrazol-3-ol

68: ^13^C NMR spectrum of 4,4'-methylenebis(5-methyl-1H-pyrazol-3-ol

69: XRD pattern of Fe_3_O_4_@SiO_2_@PCLH-TFA

1: FT-IR spectrum of diethyl 2, 6-dimethyl-1,4-dihydropyridine-3,5-dicarboxylate


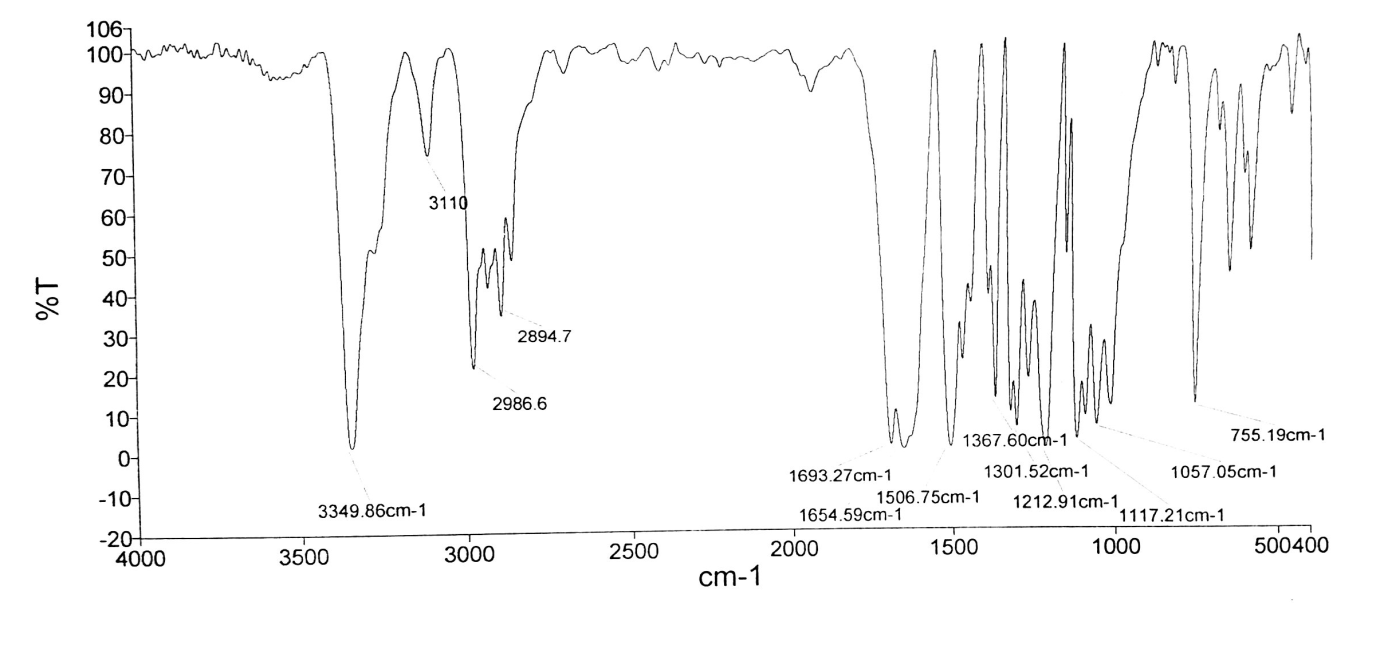


2: FT-IR spectrum of diethyl 2,6-dimethylpyridine-3,5-dicarboxylate


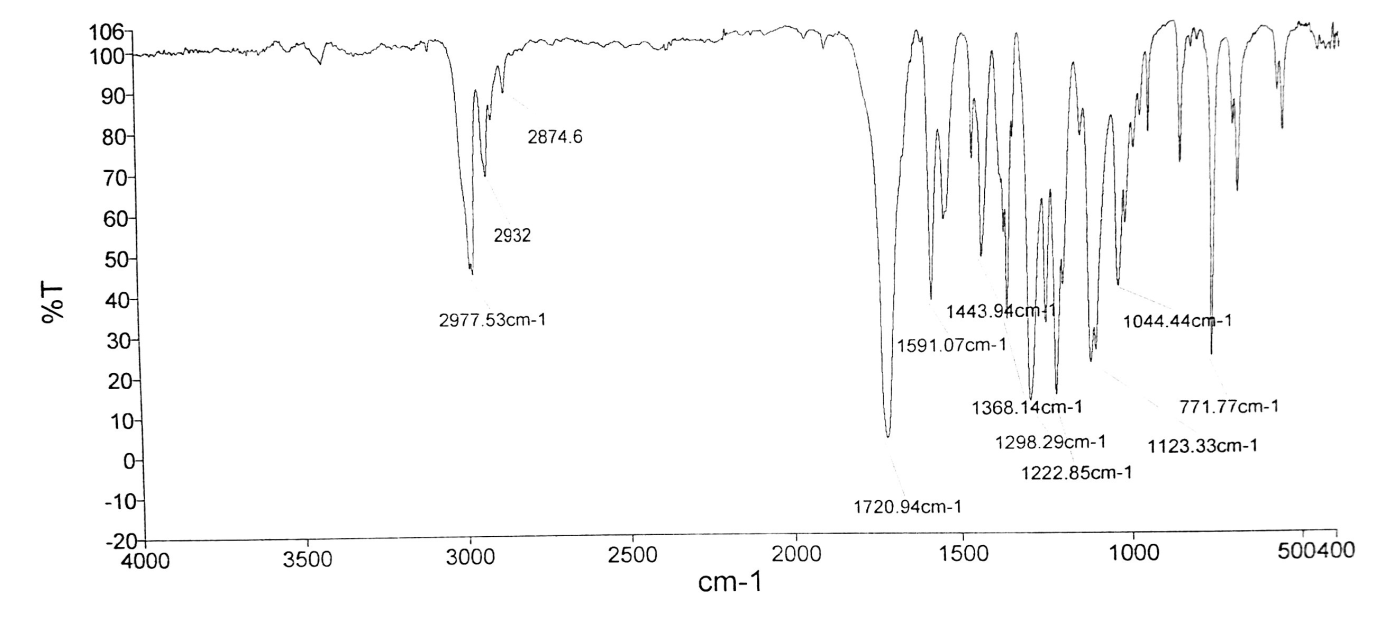


3: FT-IR spectrum of 2,6-dimethylpyridine-3,5-dicarbohydrazide


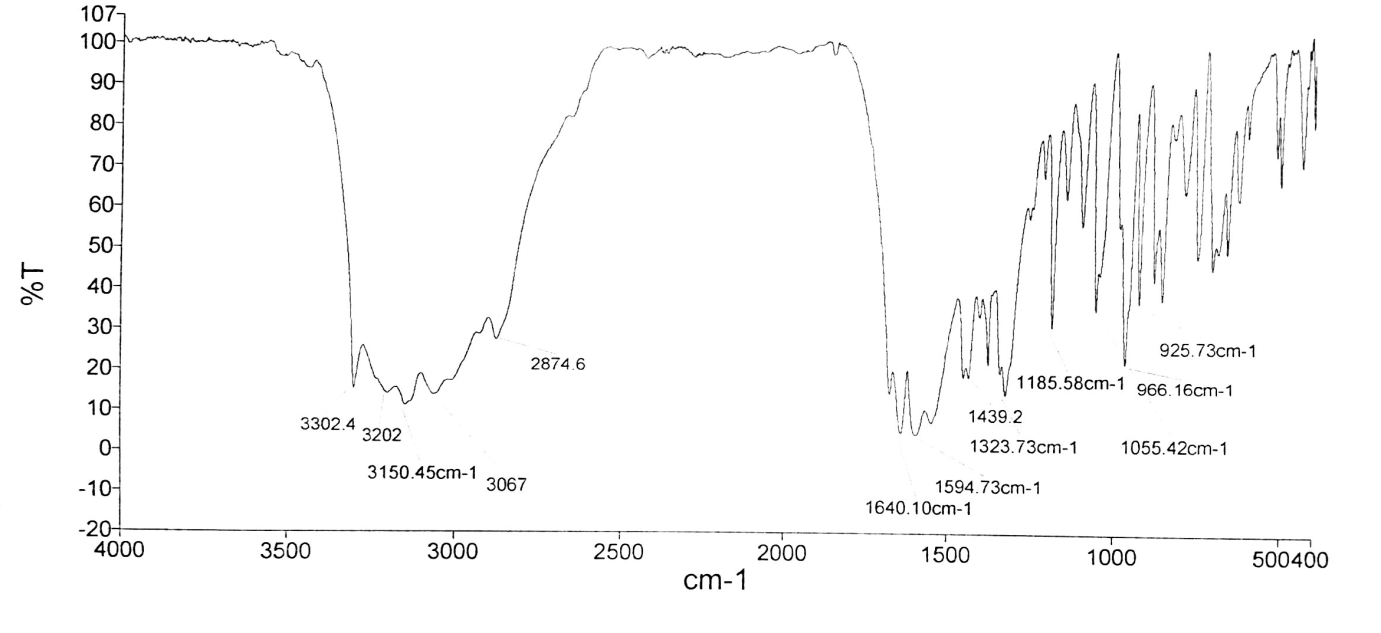


4: ^1^H NMR spectrum of 2,6-dimethylpyridine-3,5-dicarbohydrazide


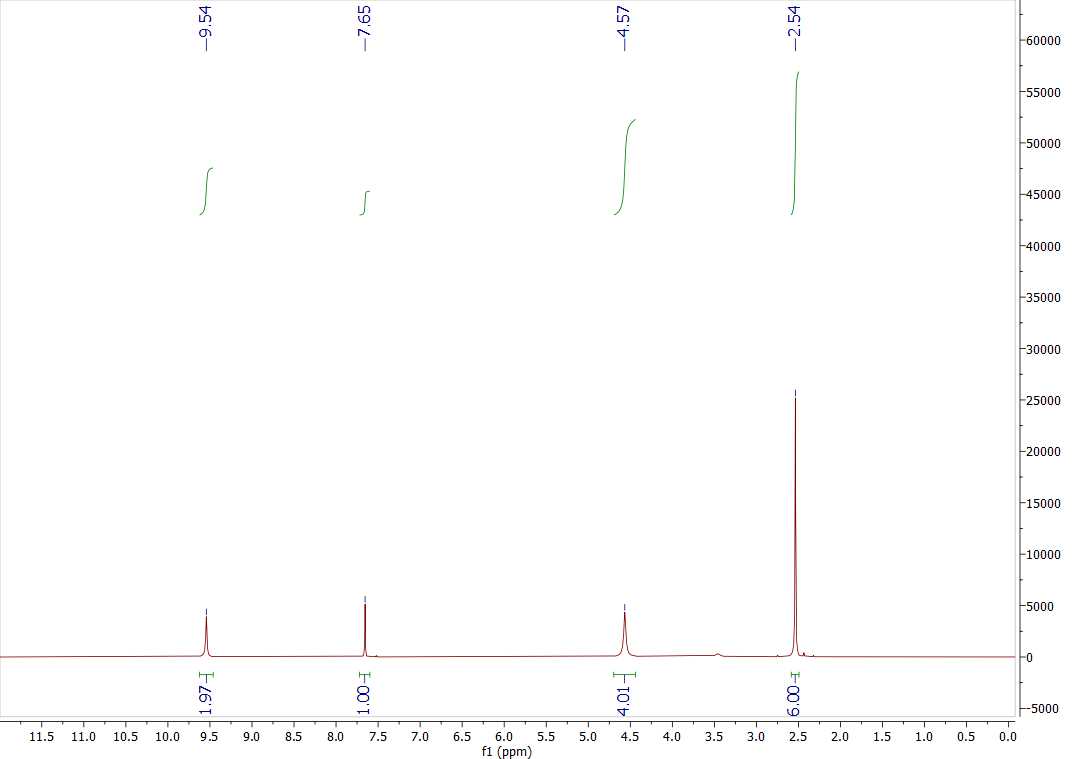


5: ^13^C NMR spectrum of 2,6-dimethylpyridine-3,5-dicarbohydrazide


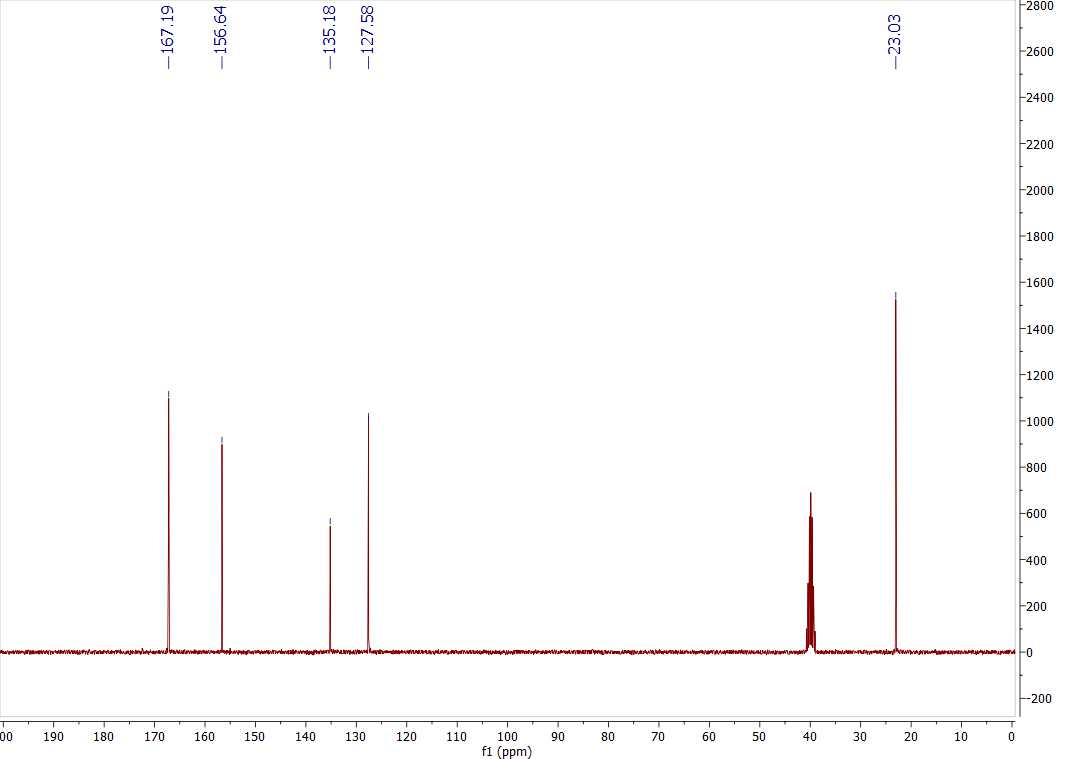


6: FT-IR spectrum of 2,2'-(2,6-dimethylpyridine-3,5-dicarbonyl)bis(*N*-(3-(triethoxysilyl)propyl)hydrazine-1-carboxamide) (PCL)


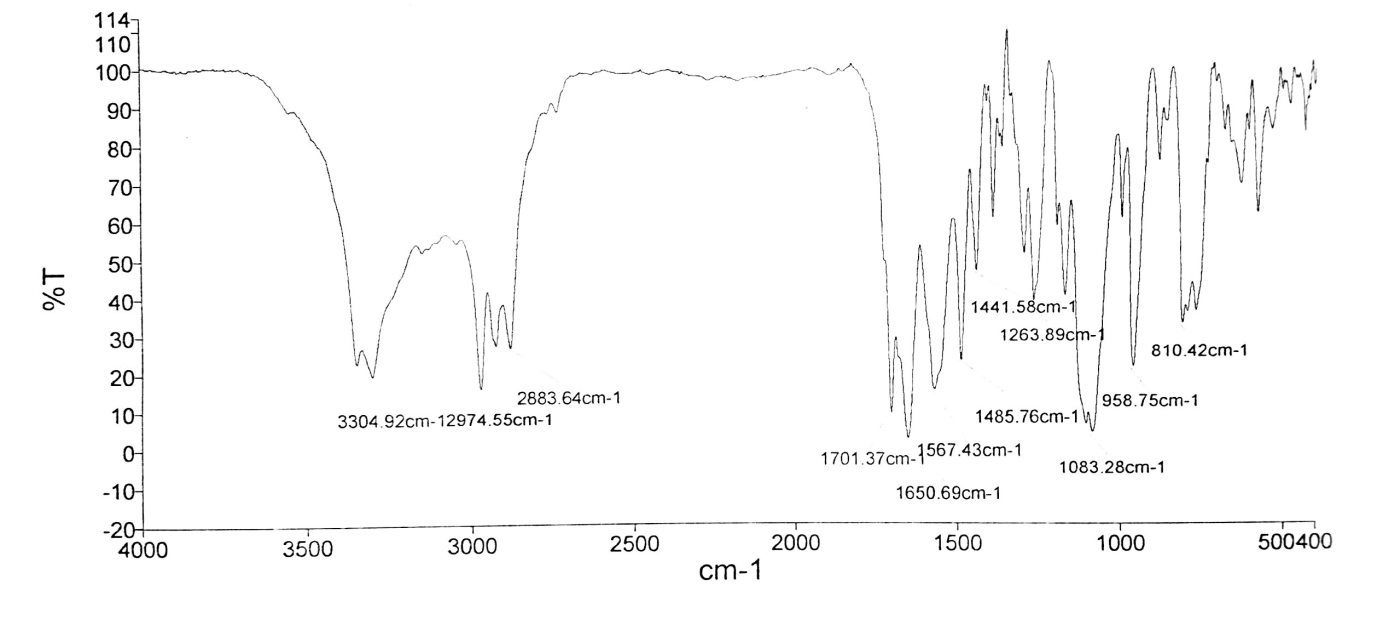


7: ^1^H NMR spectrum of 2,2'-(2,6-dimethylpyridine-3,5-dicarbonyl)bis(*N*-(3-(triethoxysilyl)propyl)hydrazine-1-carboxamide) (PCL)


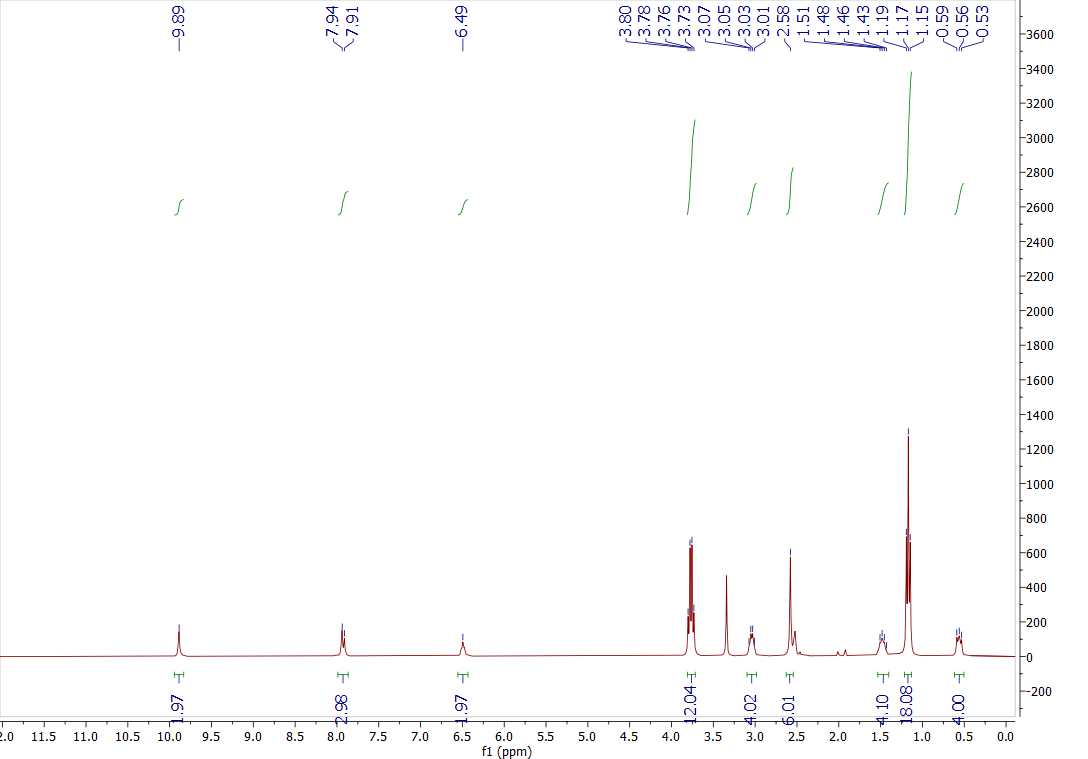


8: ^13^C NMR spectrum of 2,2'-(2,6-dimethylpyridine-3,5-dicarbonyl)bis(*N*-(3-(triethoxysilyl)propyl)hydrazine-1-carboxamide) (PCL)


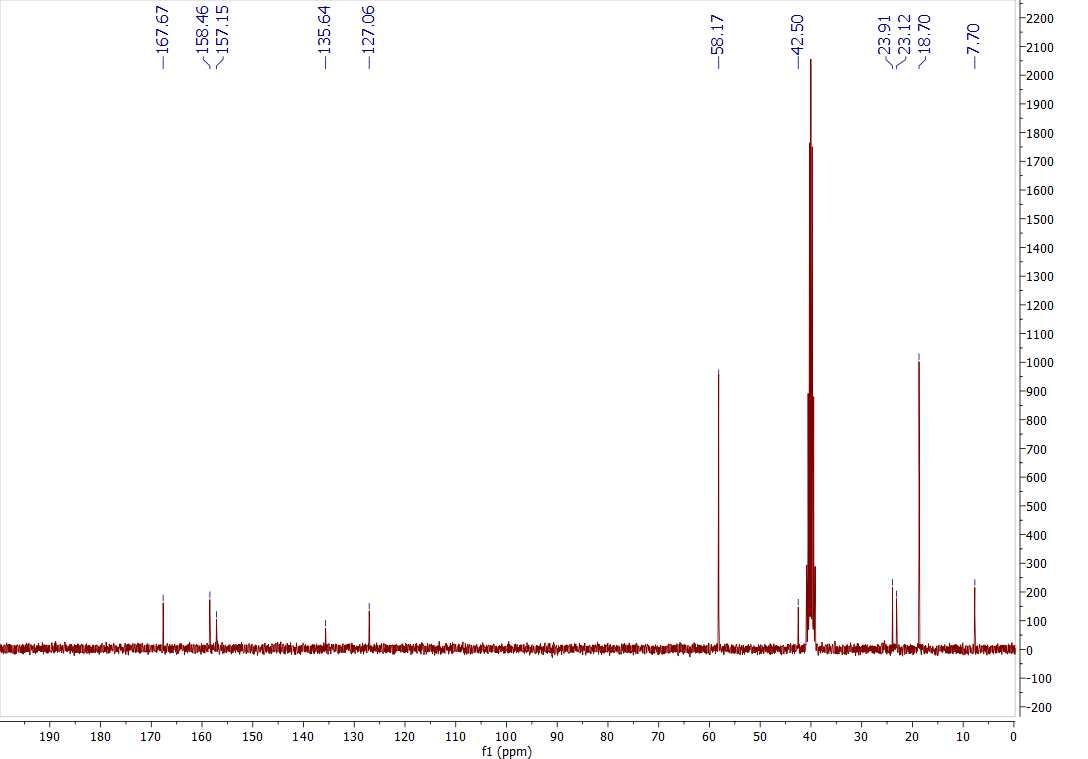


9: FT-IR spectrum of 4-acetylphenyl benzenesulfonate


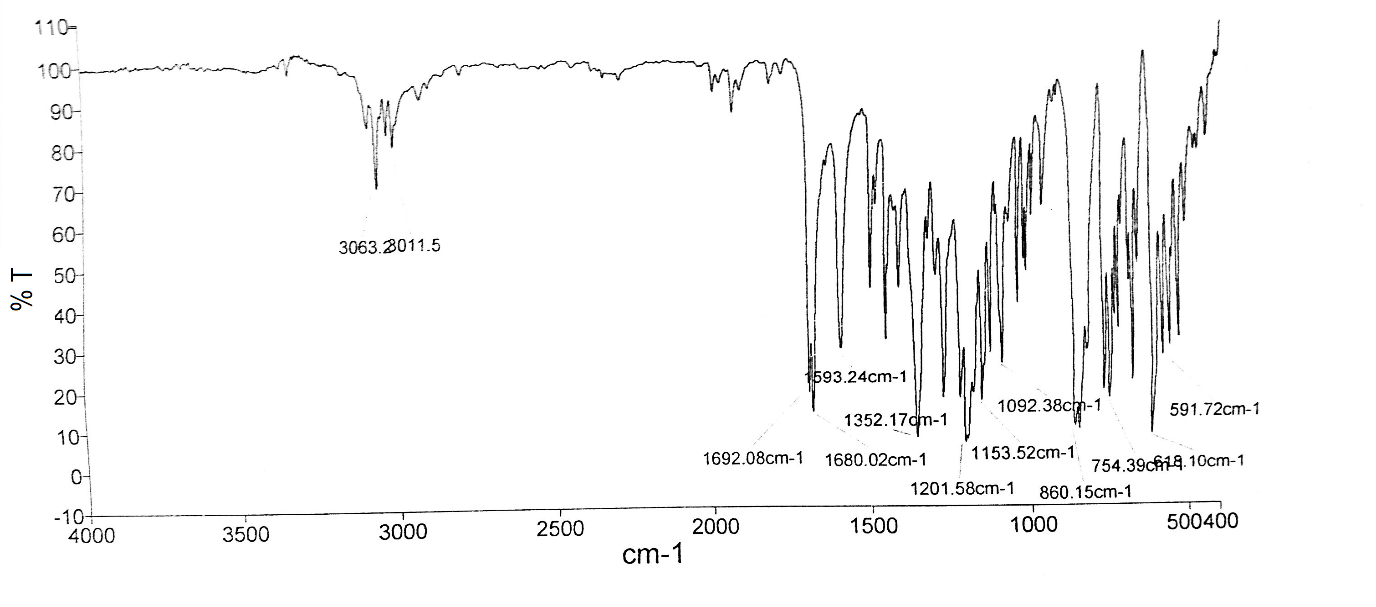

10: ^1^H NMR spectrum of 4-acetylphenyl benzenesulfonate
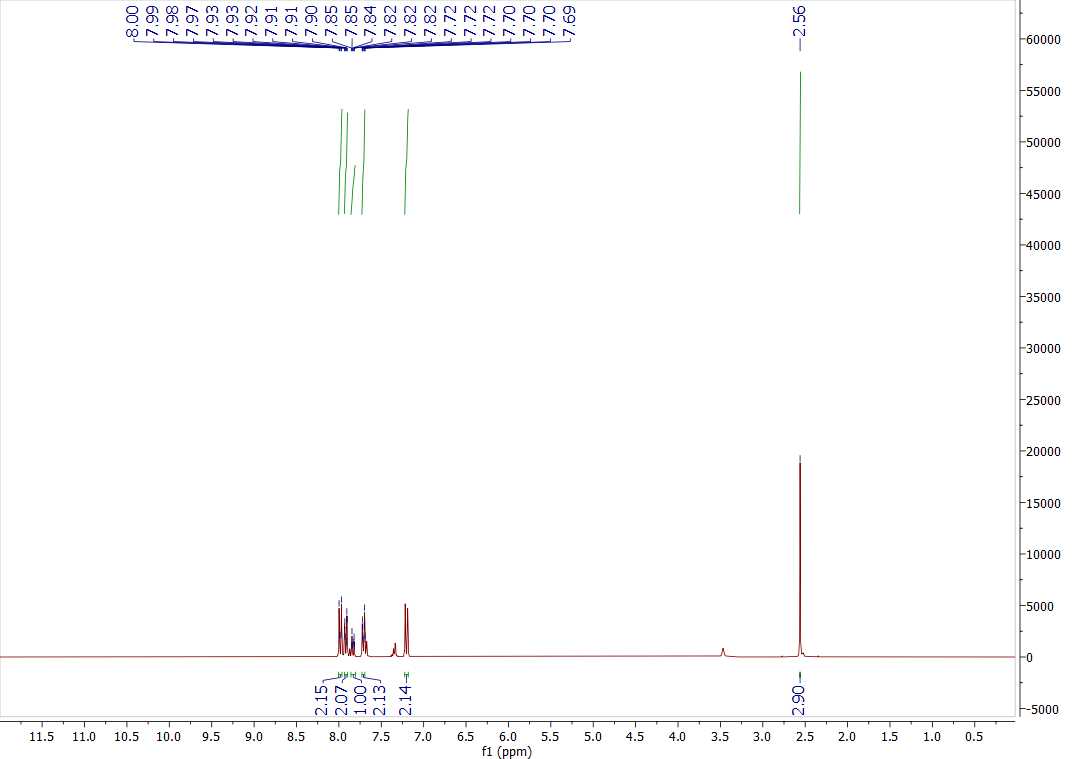


11: FT-IR spectrum of 4-acetylphenyl 4-methylbenzenesulfonate


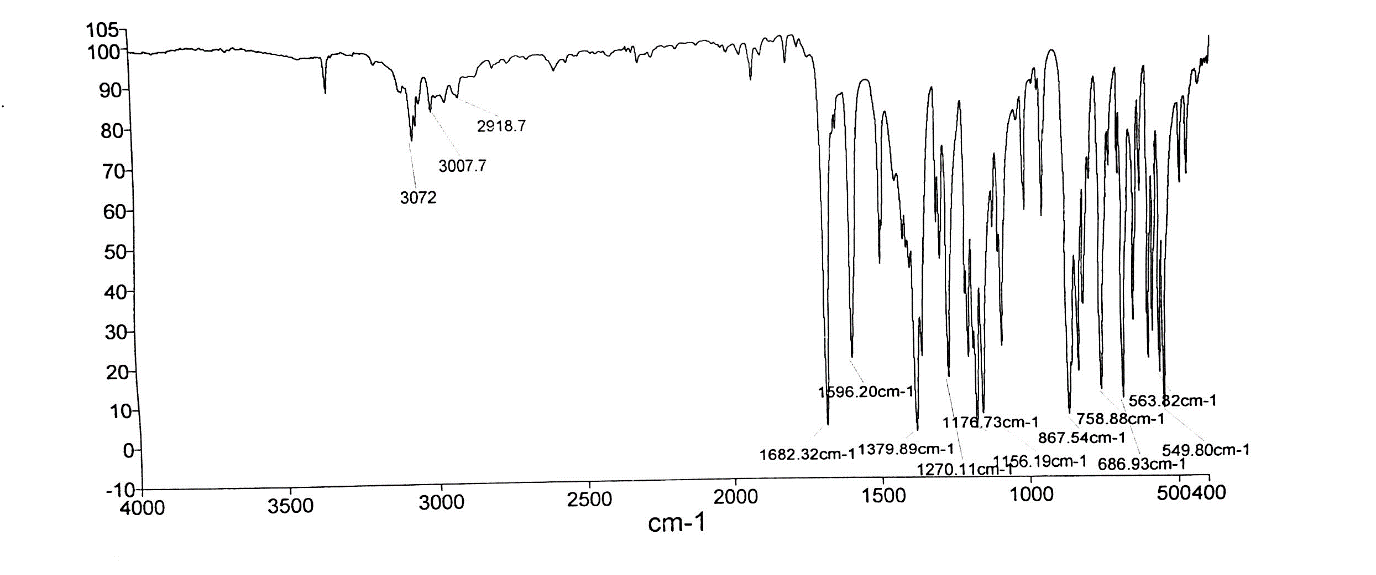


12: ^1^H NMR spectrum of 4-acetylphenyl 4-methylbenzenesulfonate


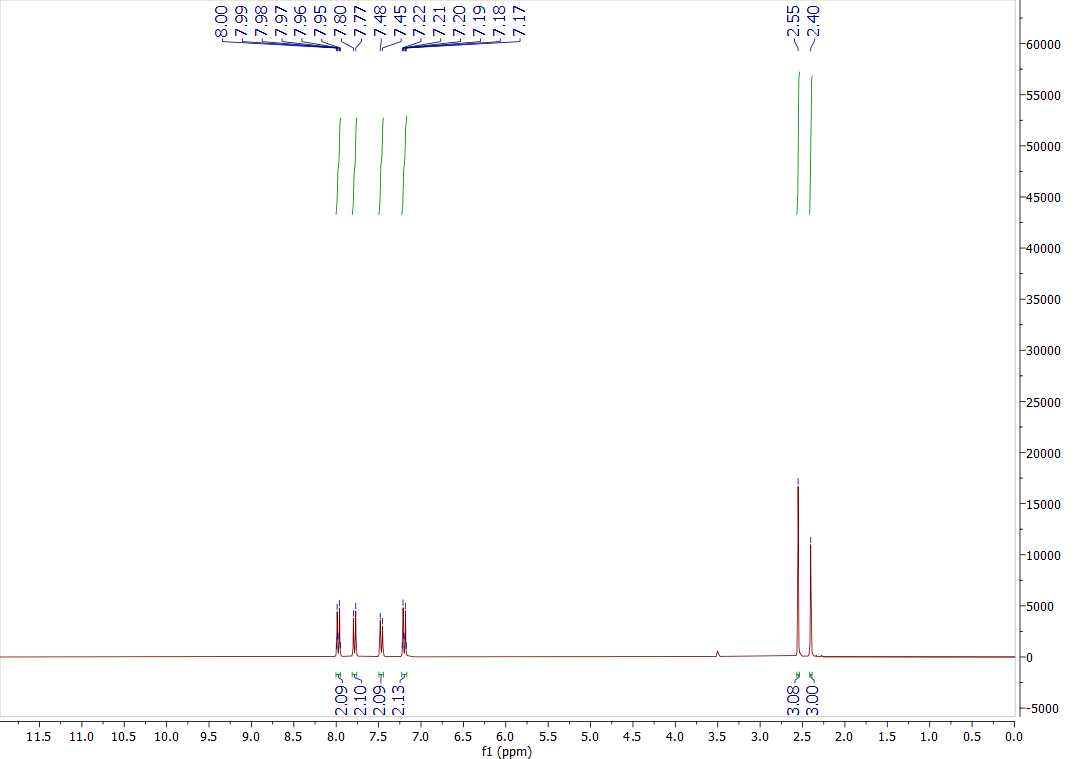


13: ^13^C NMR spectrum of 4-acetylphenyl 4-methylbenzenesulfonate


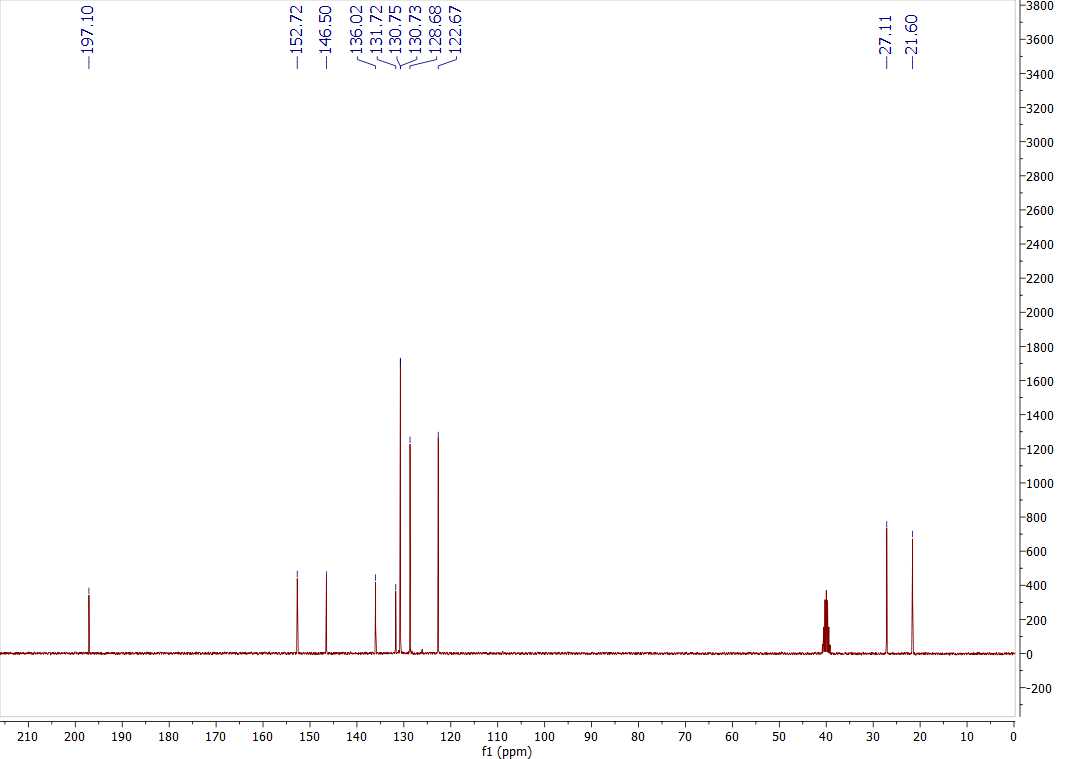


14: FT-IR spectrum of (4-phenylpyridine-2,6-diyl)bis(4,1-phenylene) bis(4-methylbenzenesulfonate) (1a)


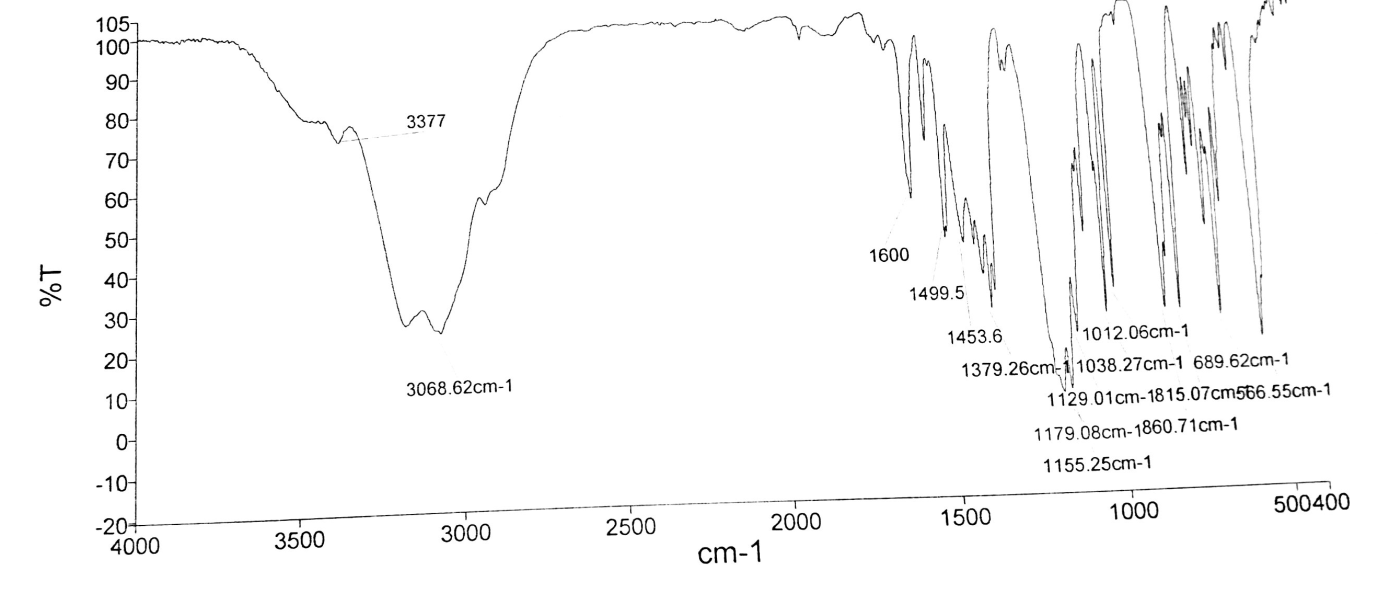


15: ^1^H NMR spectrum of (4-phenylpyridine-2,6-diyl)bis(4,1-phenylene) bis(4-methylbenzenesulfonate) (1a)


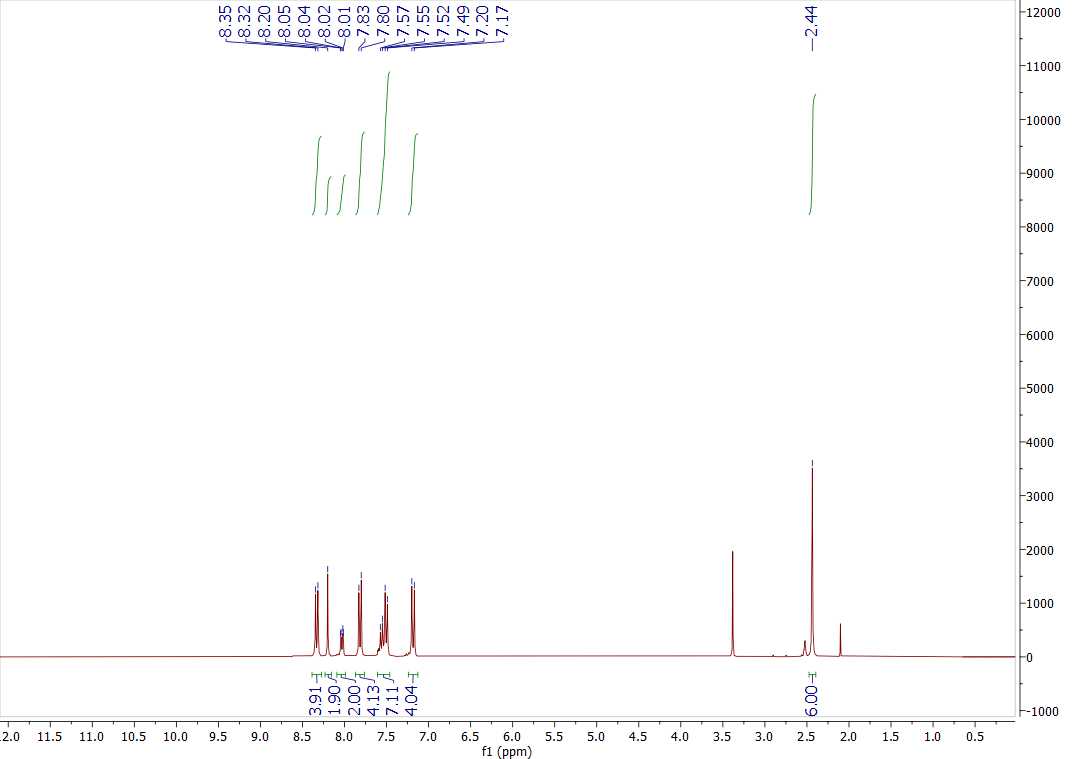


16: ^13^C NMR spectrum of (4-phenylpyridine-2,6-diyl)bis(4,1-phenylene) bis(4-methylbenzenesulfonate) (1a)


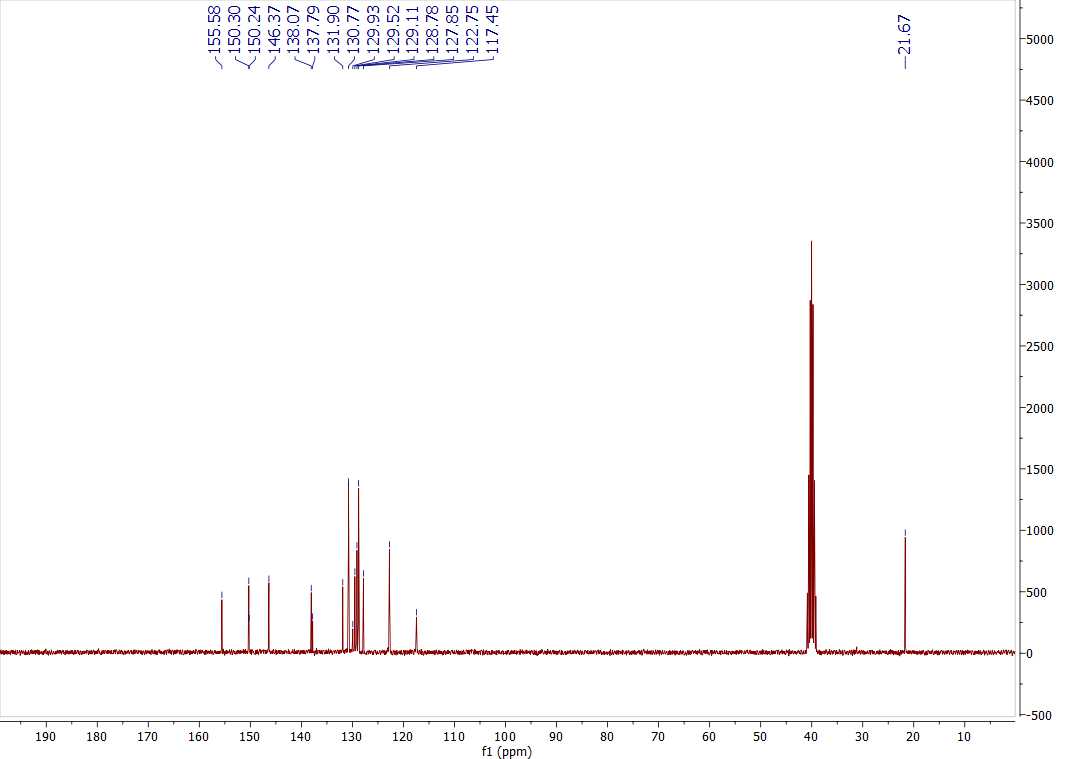


17: FT-IR spectrum of (4-(4-hydroxyphenyl)pyridine-2,6-diyl)bis(4,1-phenylene) bis(4-methylbenzenesulfonate) (1b)


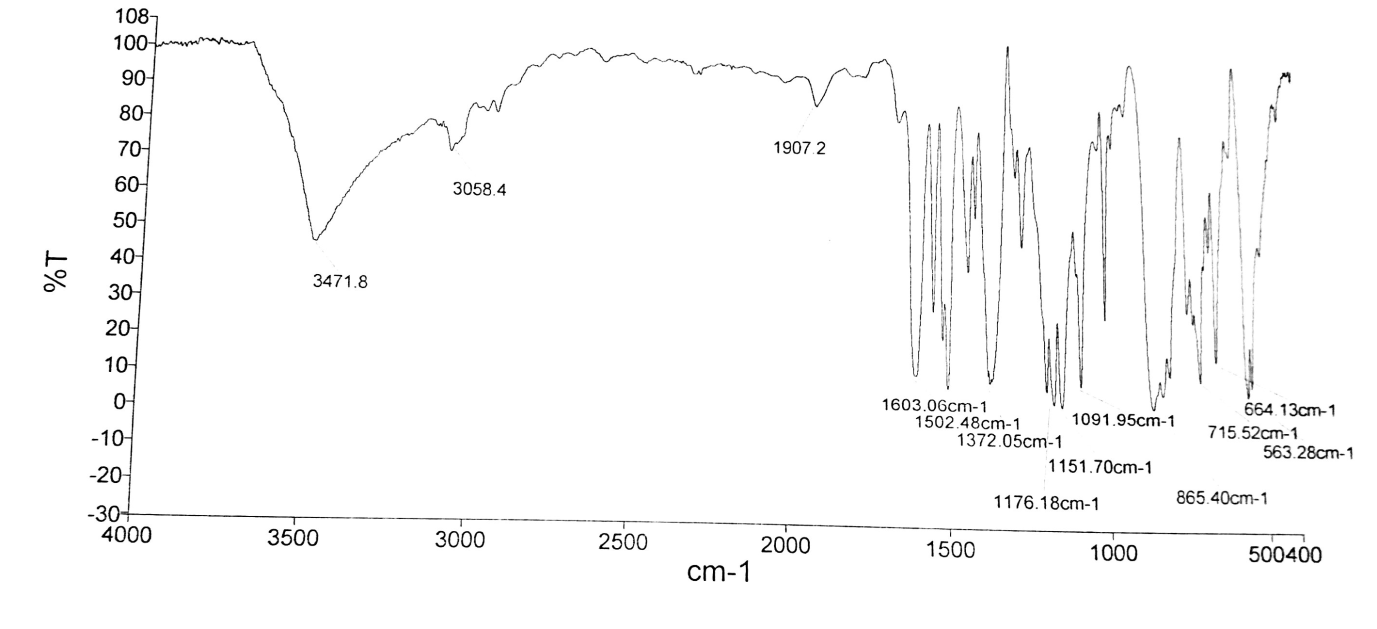


18: ^1^H NMR spectrum of (4-(4-hydroxyphenyl)pyridine-2,6-diyl)bis(4,1-phenylene) bis(4-methylbenzenesulfonate) (1b)


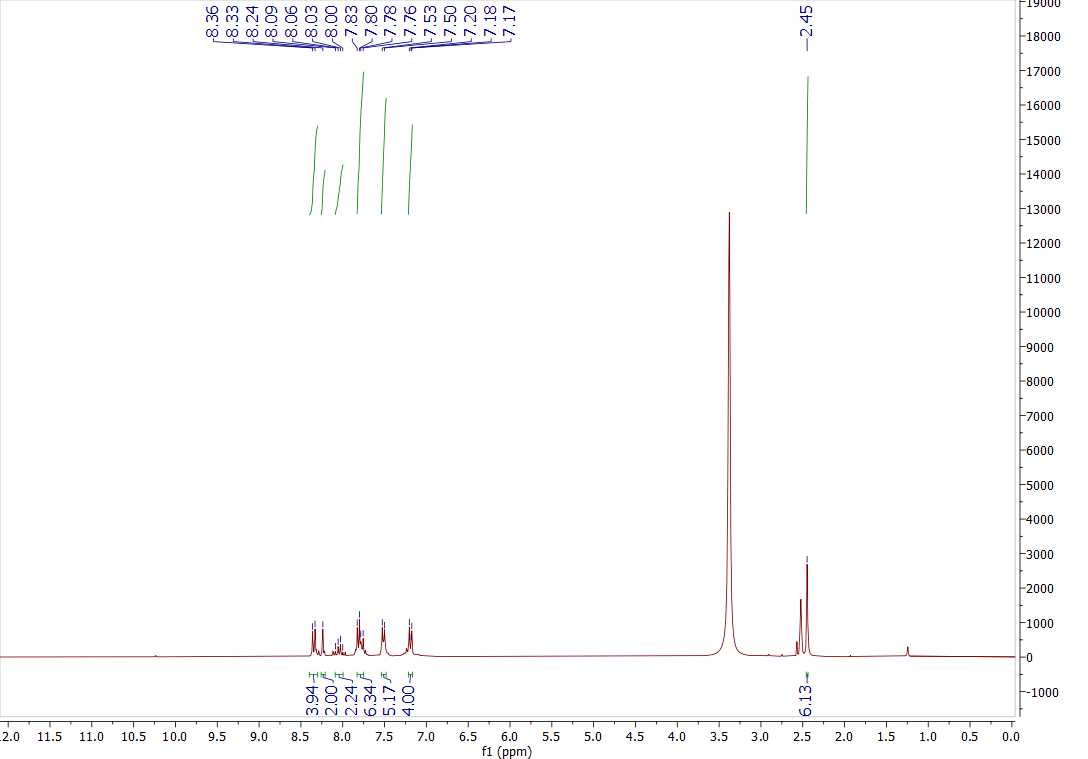


19: ^13^C NMR spectrum of (4-(4-hydroxyphenyl)pyridine-2,6-diyl)bis(4,1-phenylene) bis(4-methylbenzenesulfonate) (1b)


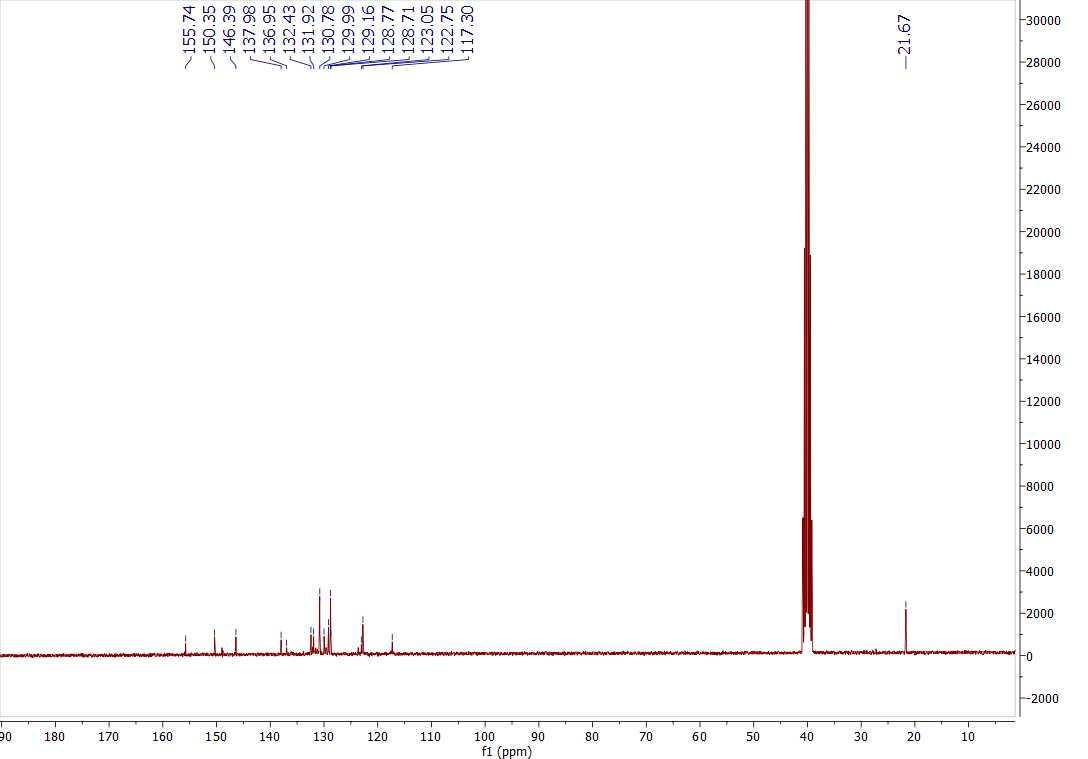


20: FT-IR spectrum of (4-(4-methoxyphenyl)pyridine-2,6-diyl)bis(4,1-phenylene) bis(4-methylbenzenesulfonate) (1c)


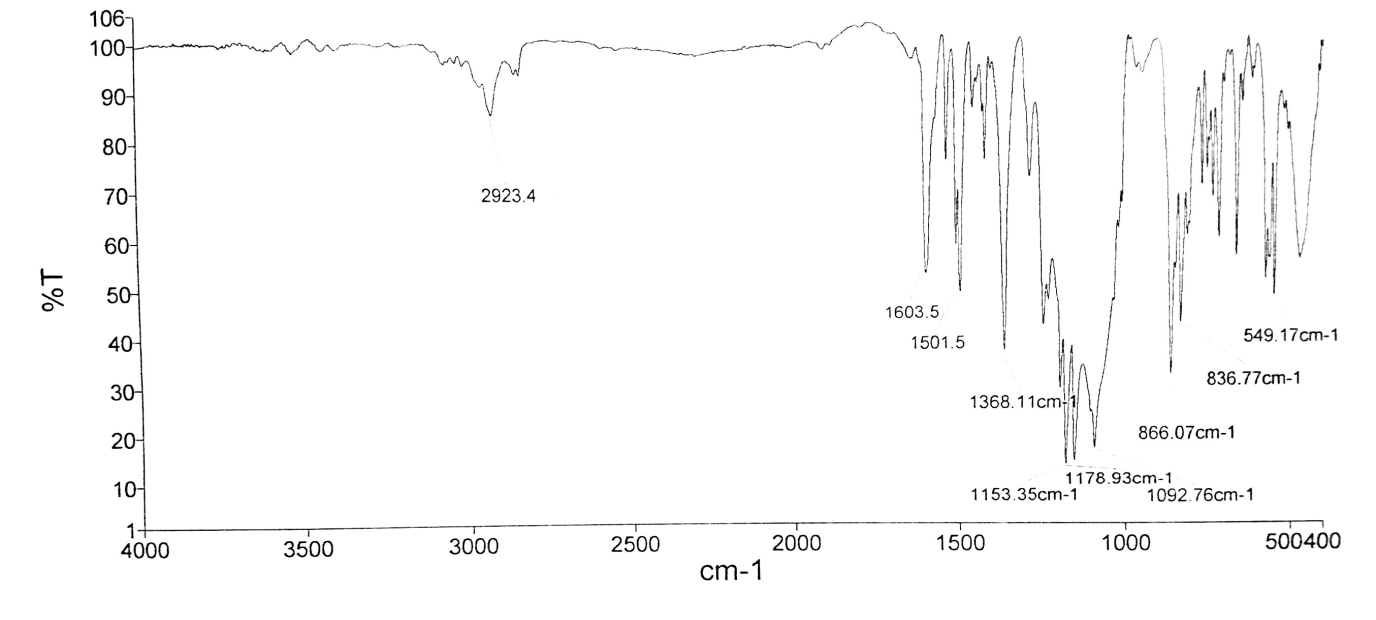


21: ^1^H NMR spectrum of (4-(4-methoxyphenyl)pyridine-2,6-diyl)bis(4,1-phenylene) bis(4-methylbenzenesulfonate) (1c)


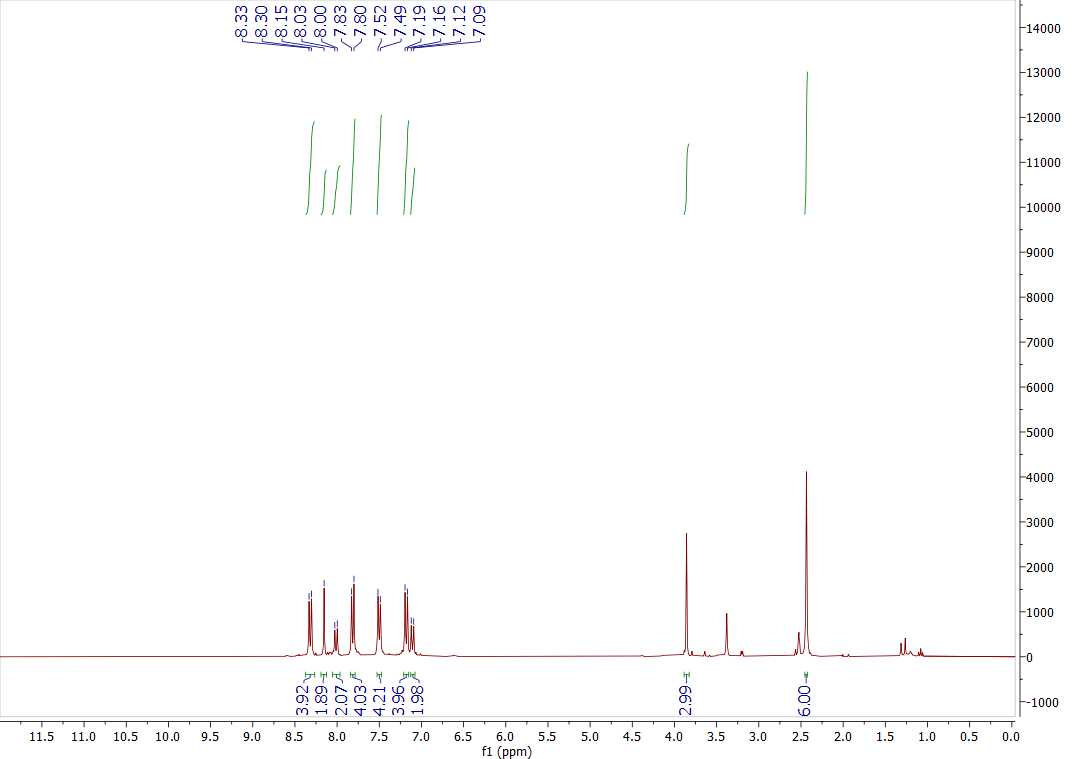


22: ^13^C NMR spectrum of (4-(4-methoxyphenyl)pyridine-2,6-diyl)bis(4,1-phenylene) bis(4-methylbenzenesulfonate) (1c)


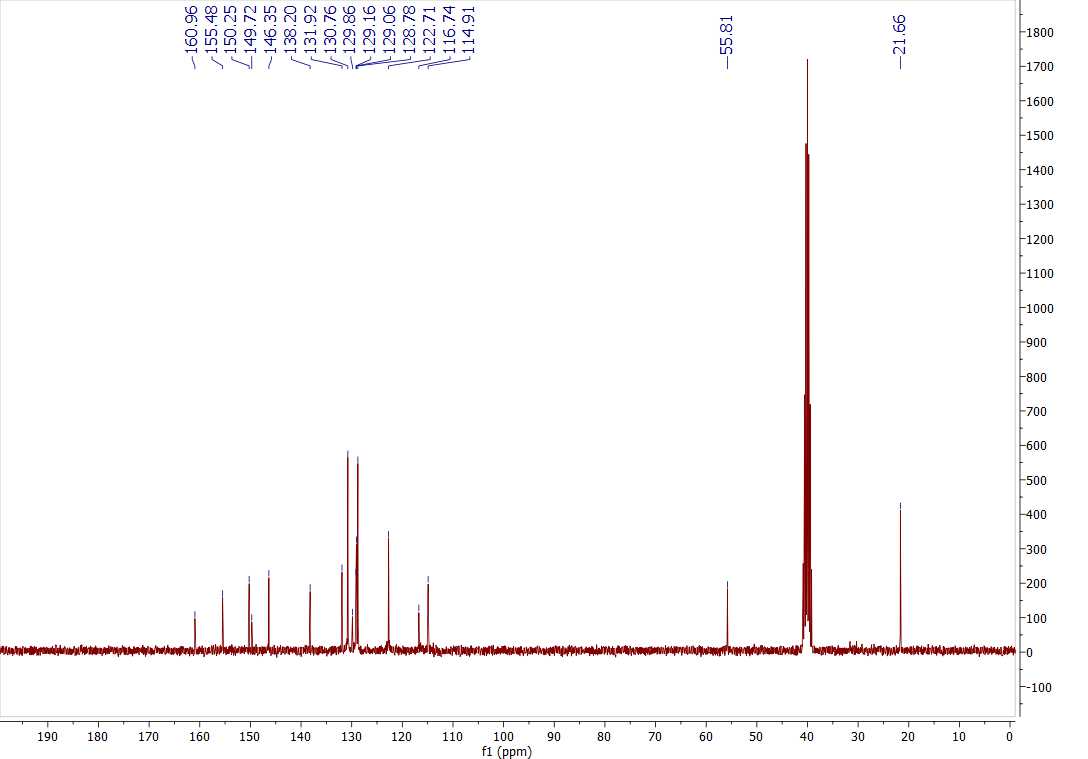


23: FT-IR spectrum of (4-(2-methoxyphenyl)pyridine-2,6-diyl)bis(4,1-phenylene) bis(4-methylbenzenesulfonate) (1d)


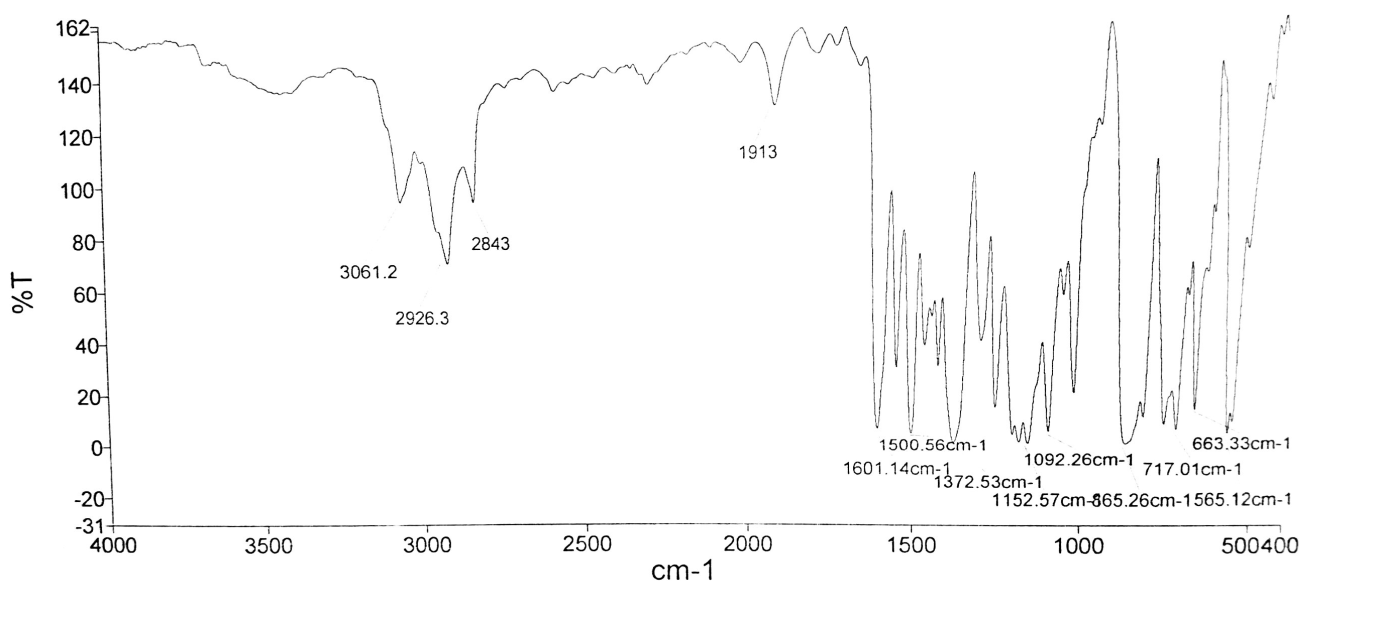


24: ^1^H NMR spectrum of (4-(2-methoxyphenyl)pyridine-2,6-diyl)bis(4,1-phenylene) bis(4-methylbenzenesulfonate) (1d)


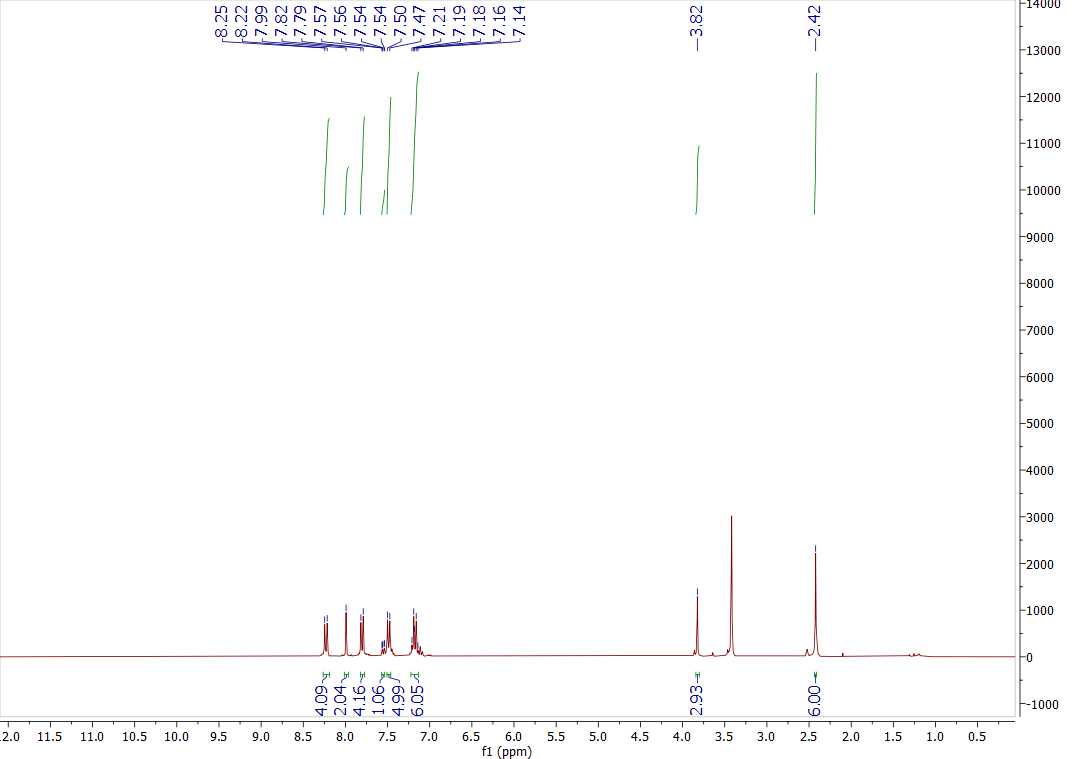


25: ^13^C NMR spectrum of (4-(2-methoxyphenyl)pyridine-2,6-diyl)bis(4,1-phenylene) bis(4-methylbenzenesulfonate) (1d)


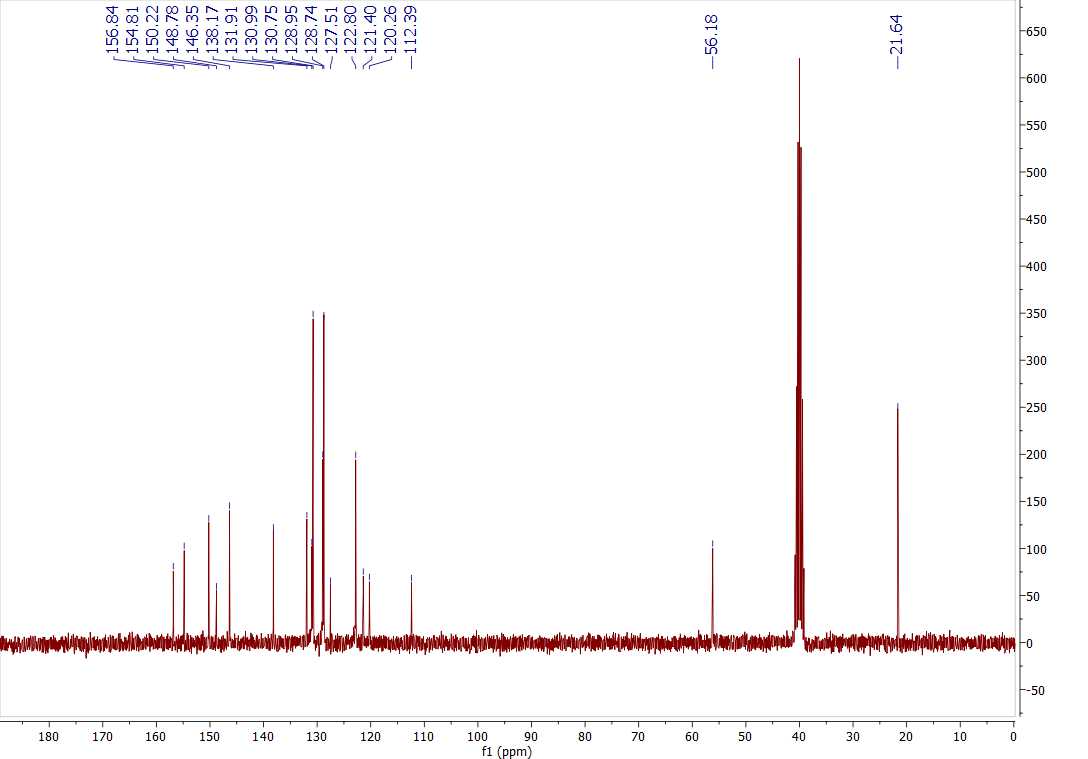


26: Mass spectrum of (4-(2-methoxyphenyl)pyridine-2,6-diyl)bis(4,1-phenylene) bis(4-methylbenzenesulfonate) (1d)


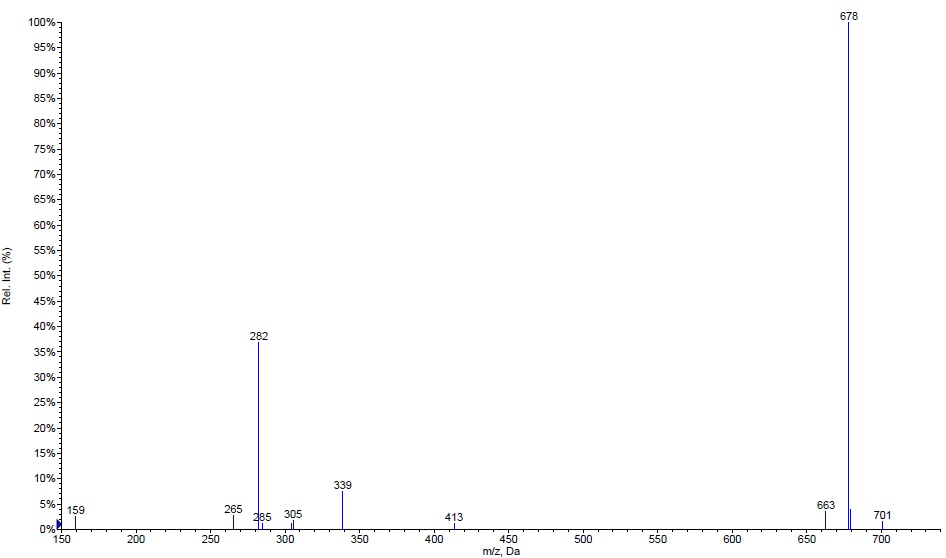


27: FT-IR spectrum of (4-(3,4-dimethoxyphenyl)pyridine-2,6-diyl)bis(4,1-phenylene) bis(4-methylbenzenesulfonate) (1e)


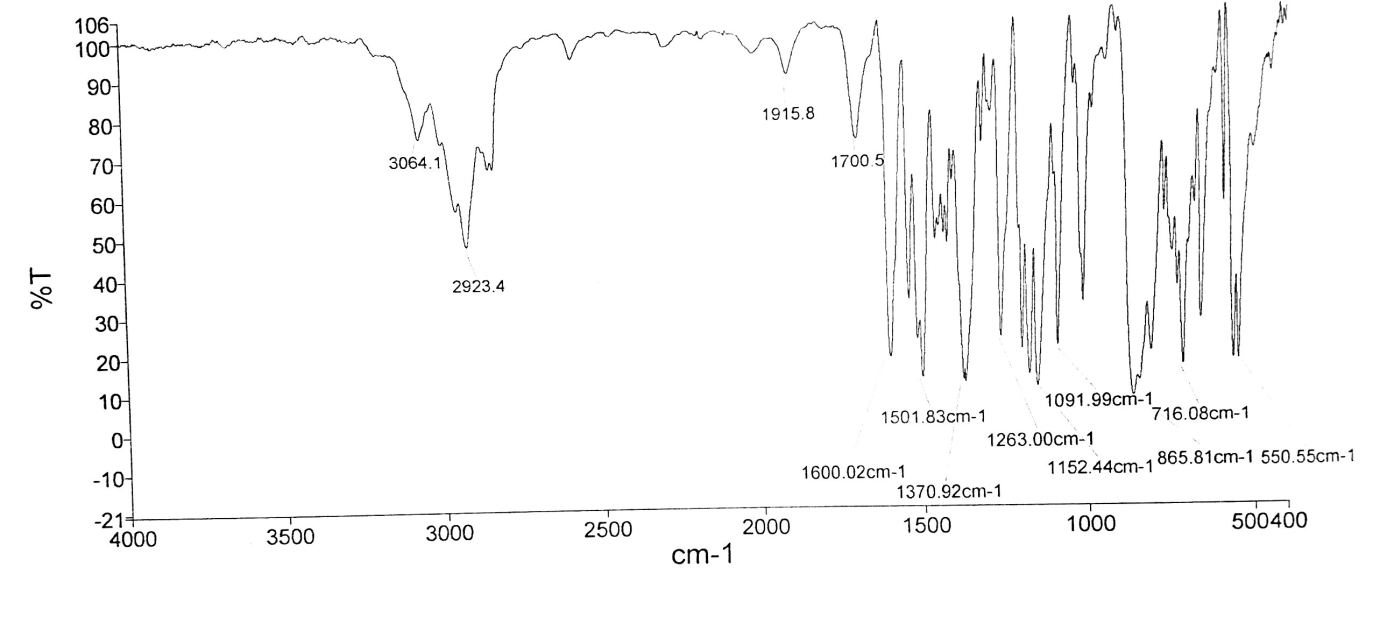


28: ^1^H NMR spectrum of (4-(3,4-dimethoxyphenyl)pyridine-2,6-diyl)bis(4,1-phenylene) bis(4-methylbenzenesulfonate) (1e)


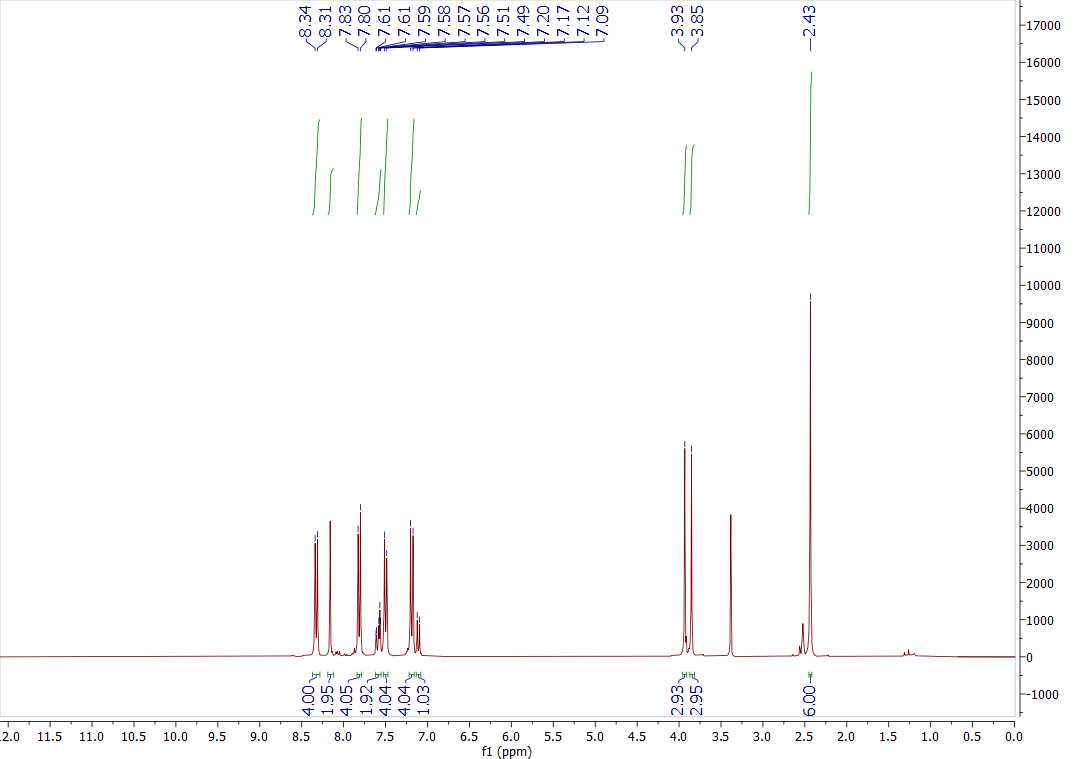


29: ^13^C NMR spectrum of (4-(3,4-dimethoxyphenyl)pyridine-2,6-diyl)bis(4,1-phenylene) bis(4-methylbenzenesulfonate) (1e)


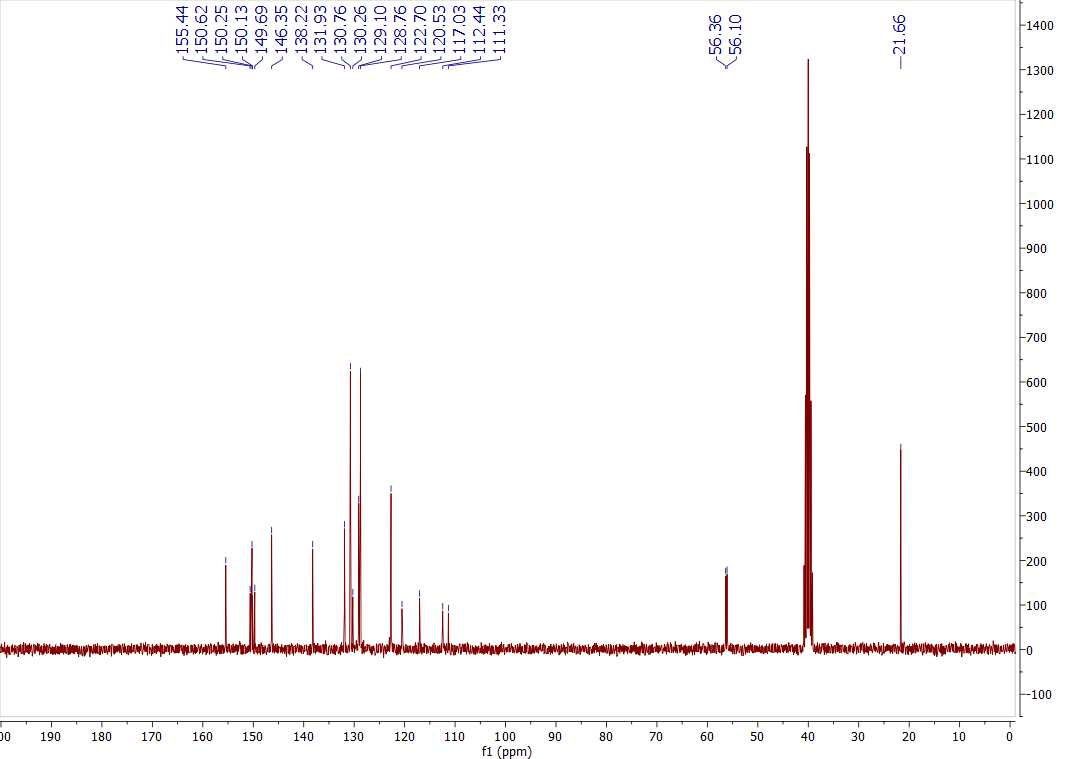


30: Mass spectrum of (4-(3,4-dimethoxyphenyl)pyridine-2,6-diyl)bis(4,1-phenylene) bis(4-methylbenzenesulfonate) (1e)


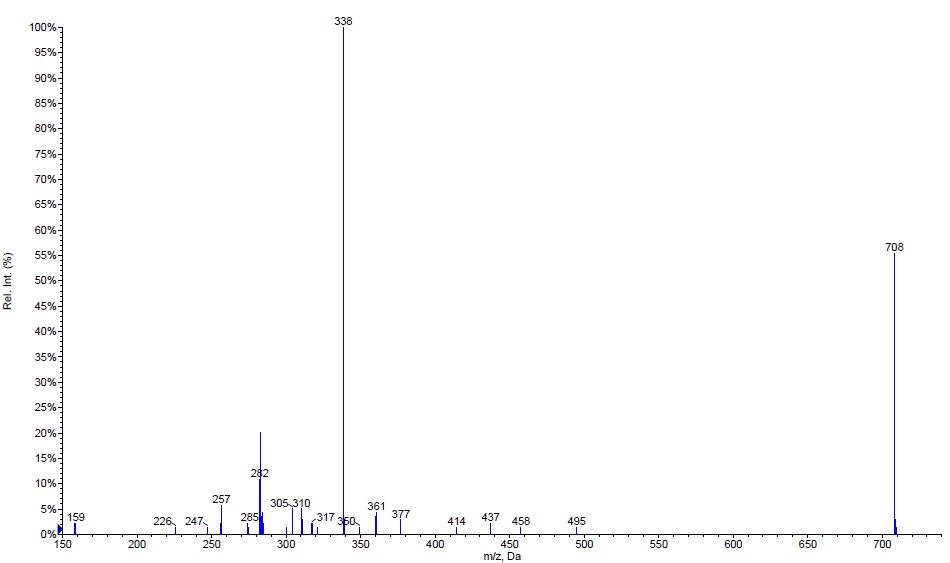


31: FT-IR spectrum of (4-(4-chlorophenyl)pyridine-2,6-diyl)bis(4,1-phenylene) bis(4-methylbenzenesulfonate) (1f)


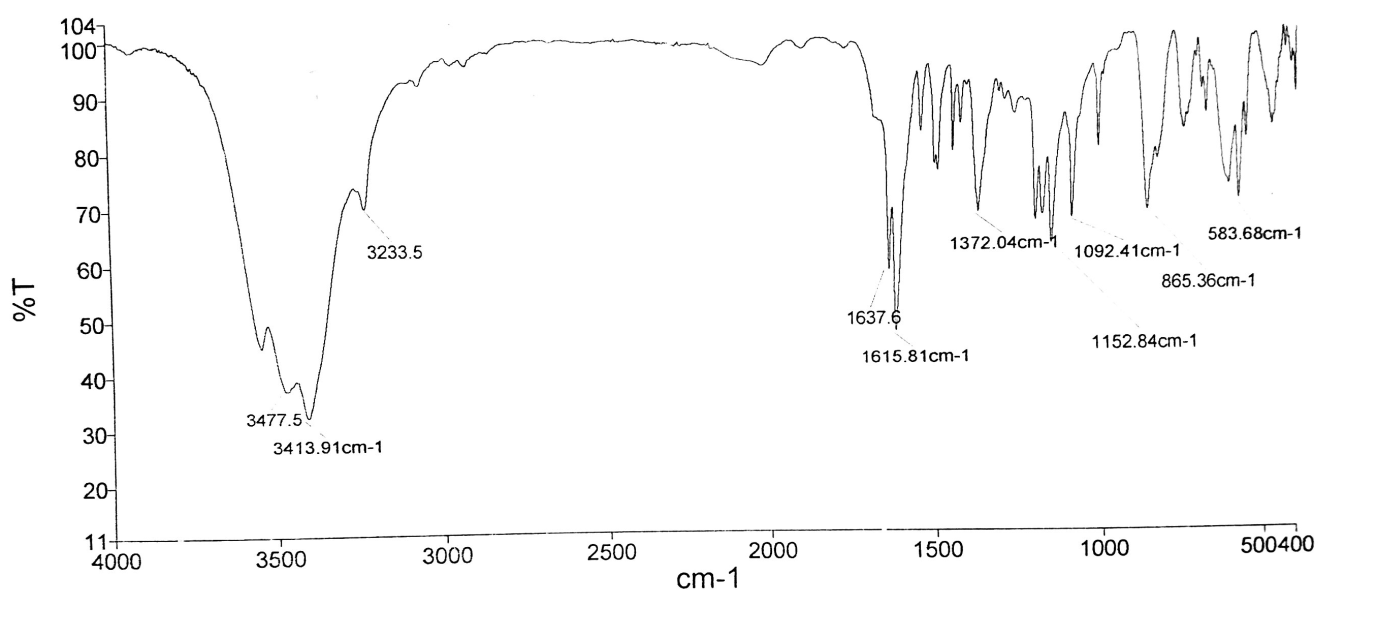


32: ^1^H NMR spectrum of (4-(4-chlorophenyl)pyridine-2,6-diyl)bis(4,1-phenylene) bis(4-methylbenzenesulfonate) (1f)


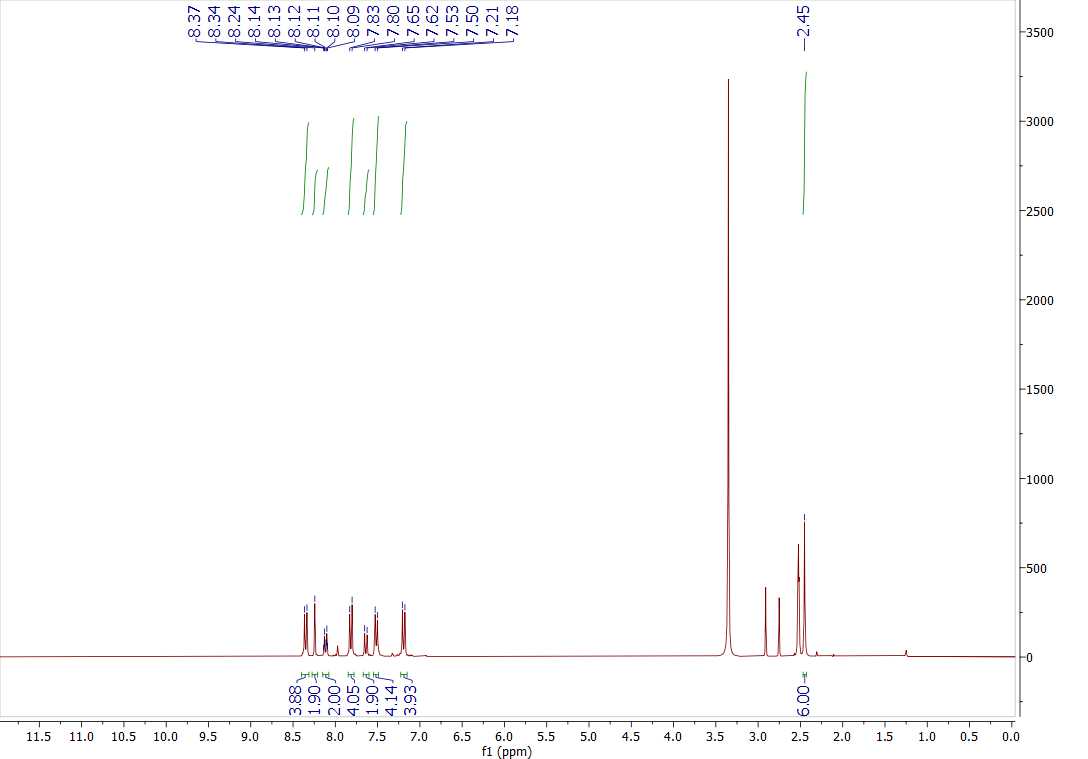


33: ^13^C NMR spectrum of (4-(4-chlorophenyl)pyridine-2,6-diyl)bis(4,1-phenylene) bis(4-methylbenzenesulfonate) (1f)


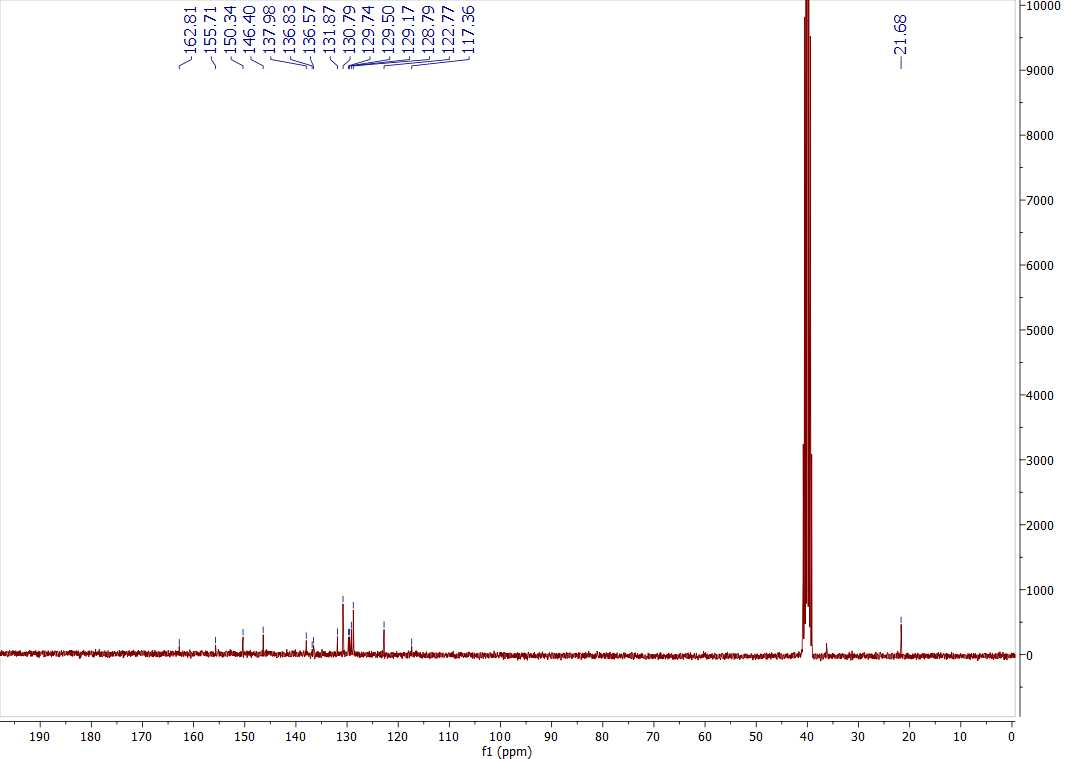


34: FT-IR spectrum of (4-(4-bromophenyl)pyridine-2,6-diyl)bis(4,1-phenylene) bis(4-methylbenzenesulfonate) (1g)


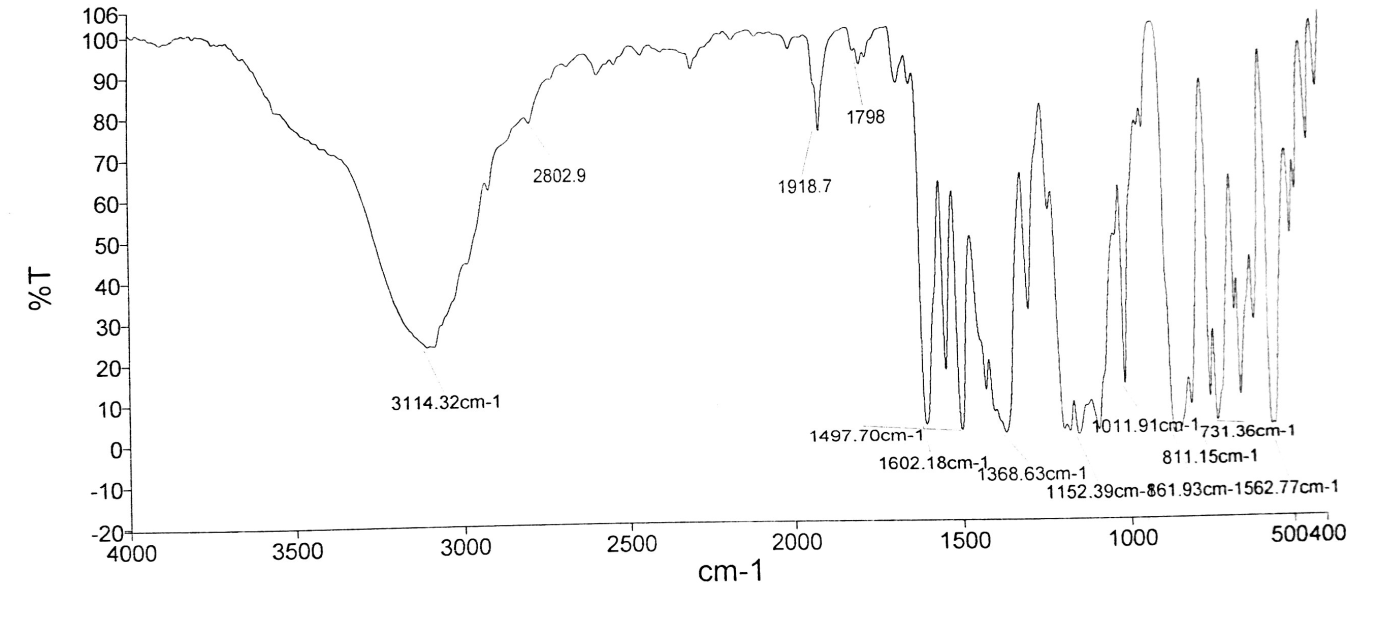


35: ^1^H NMR spectrum of (4-(4-bromophenyl)pyridine-2,6-diyl)bis(4,1-phenylene) bis(4-methylbenzenesulfonate) (1g)


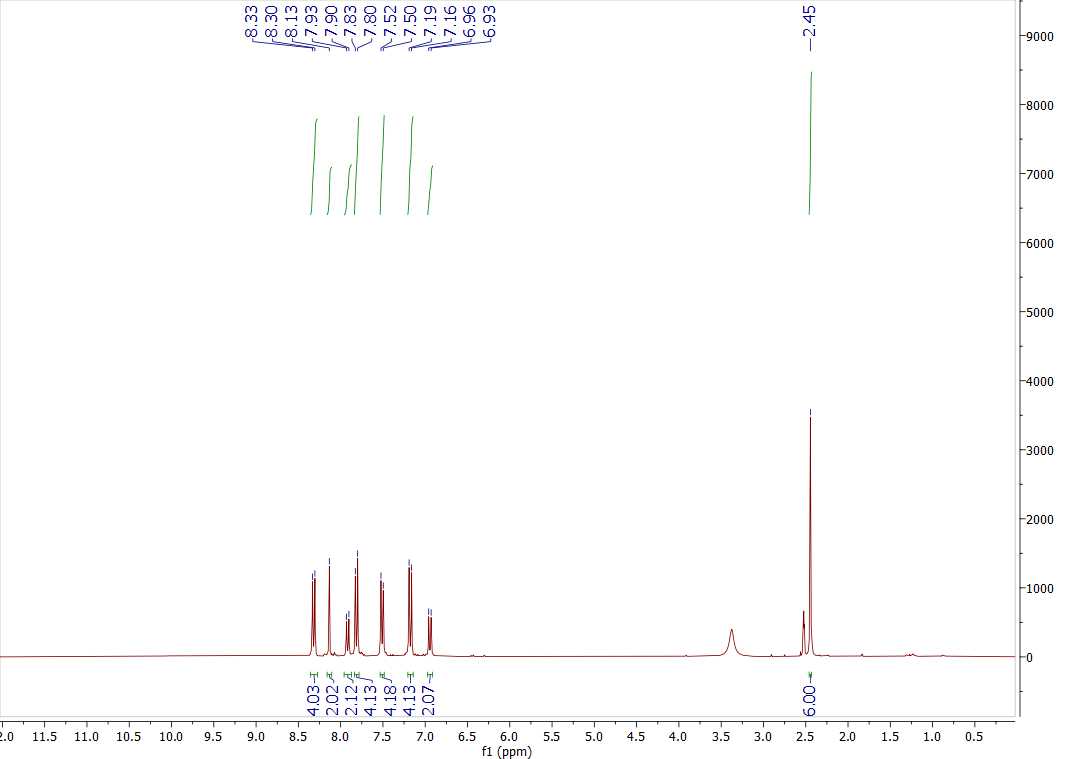


36: ^13^C NMR spectrum of (4-(4-bromophenyl)pyridine-2,6-diyl)bis(4,1-phenylene) bis(4-methylbenzenesulfonate) (1g)


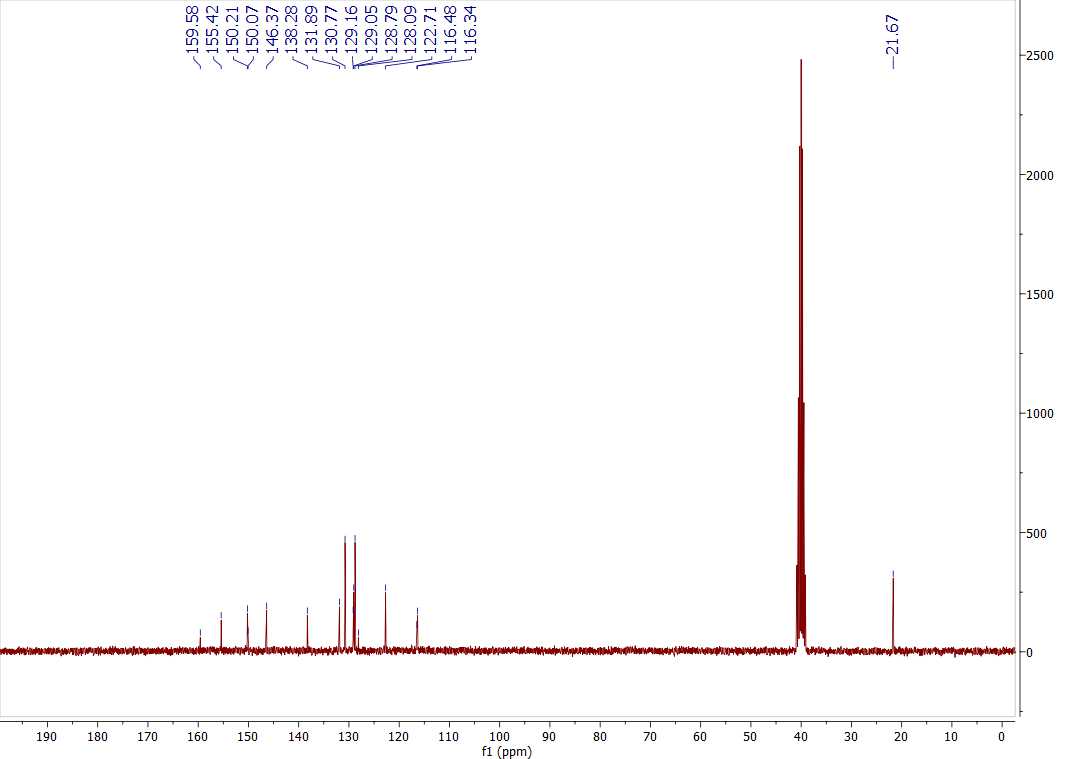


37: FT-IR spectrum of (4-(4-isopropylphenyl)pyridine-2,6-diyl)bis(4,1-phenylene) dibenzenesulfonate (2a)


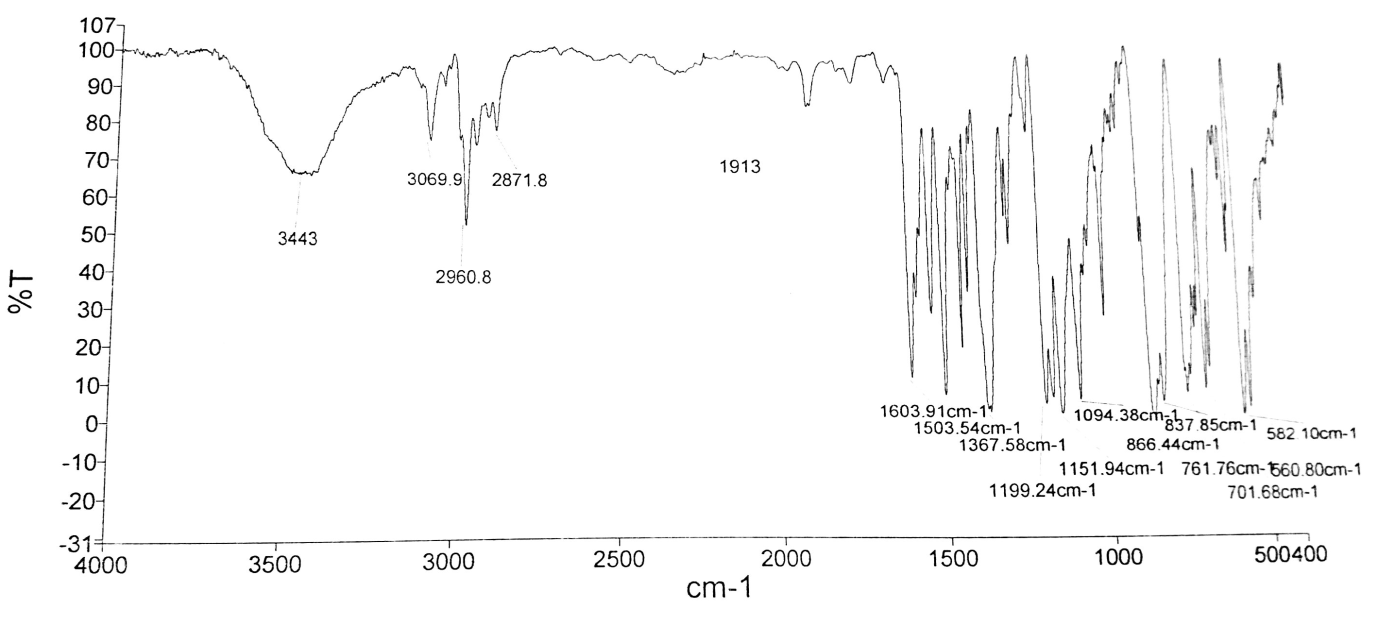


38: ^1^H NMR spectrum of (4-(4-isopropylphenyl)pyridine-2,6-diyl)bis(4,1-phenylene) dibenzenesulfonate (2a)


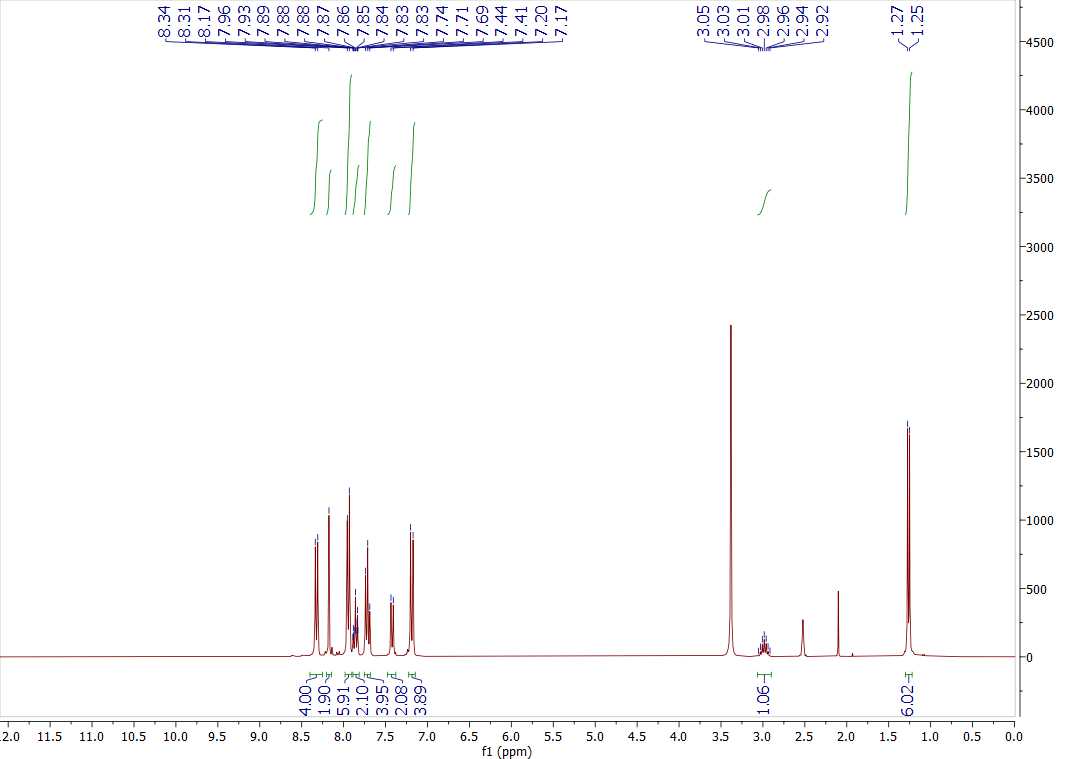


39: ^13^C NMR spectrum of (4-(4-isopropylphenyl)pyridine-2,6-diyl)bis(4,1-phenylene) dibenzenesulfonate (2a)


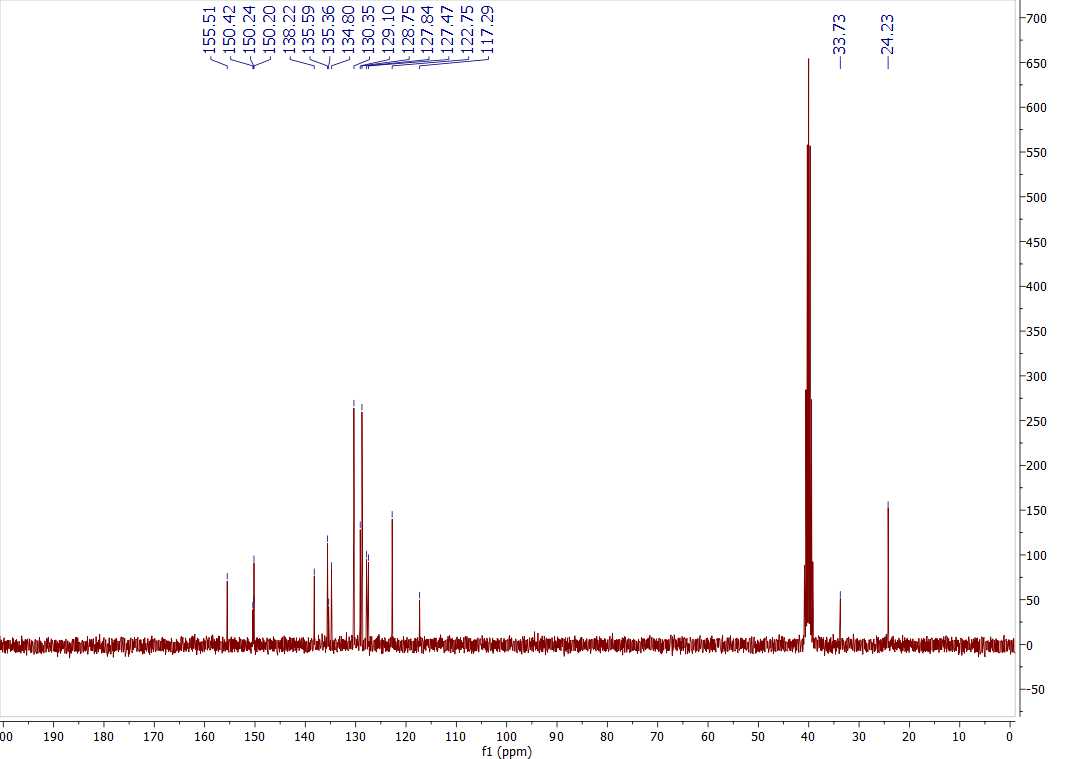


40: Mass spectrum of (4-(4-isopropylphenyl)pyridine-2,6-diyl)bis(4,1-phenylene) dibenzenesulfonate (2a)


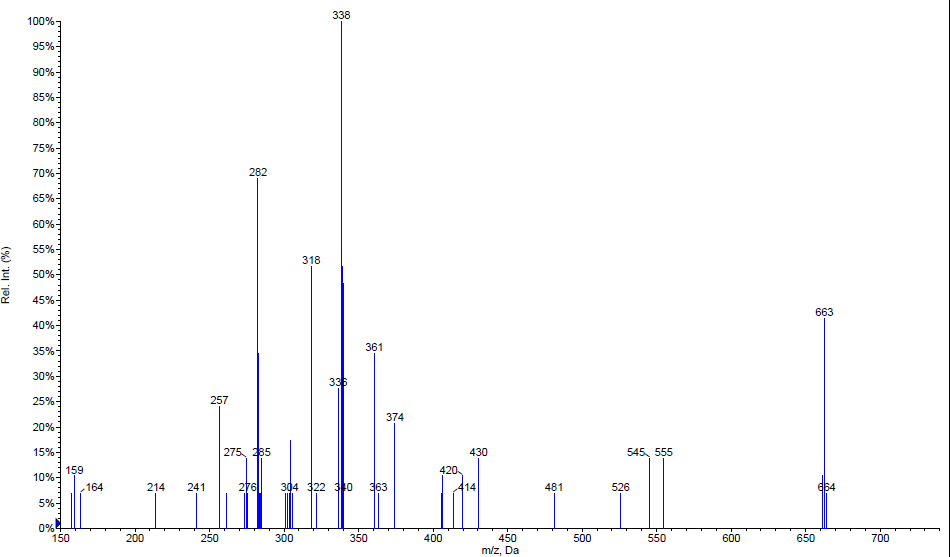


41: FT-IR spectrum of (4-(4-methoxyphenyl)pyridine-2,6-diyl)bis(4,1-phenylene) dibenzenesulfonate (2b)


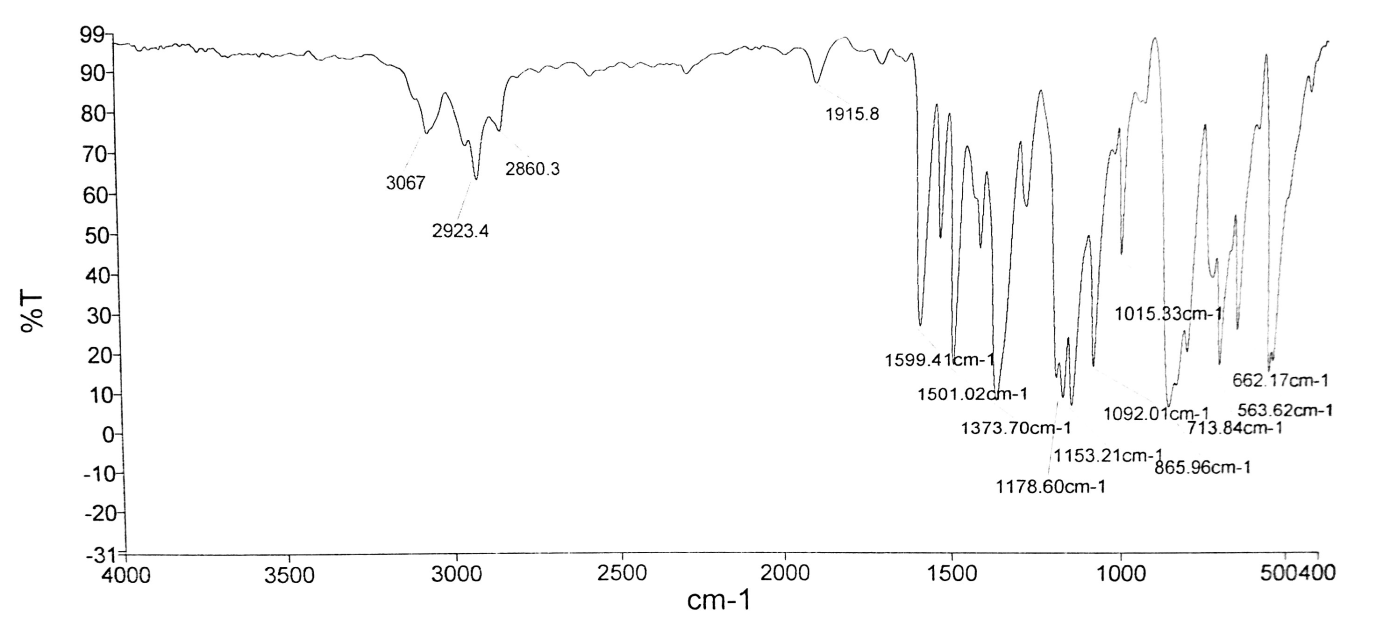


42: ^1^H NMR spectrum of (4-(4-methoxyphenyl)pyridine-2,6-diyl)bis(4,1-phenylene) dibenzenesulfonate (2b)


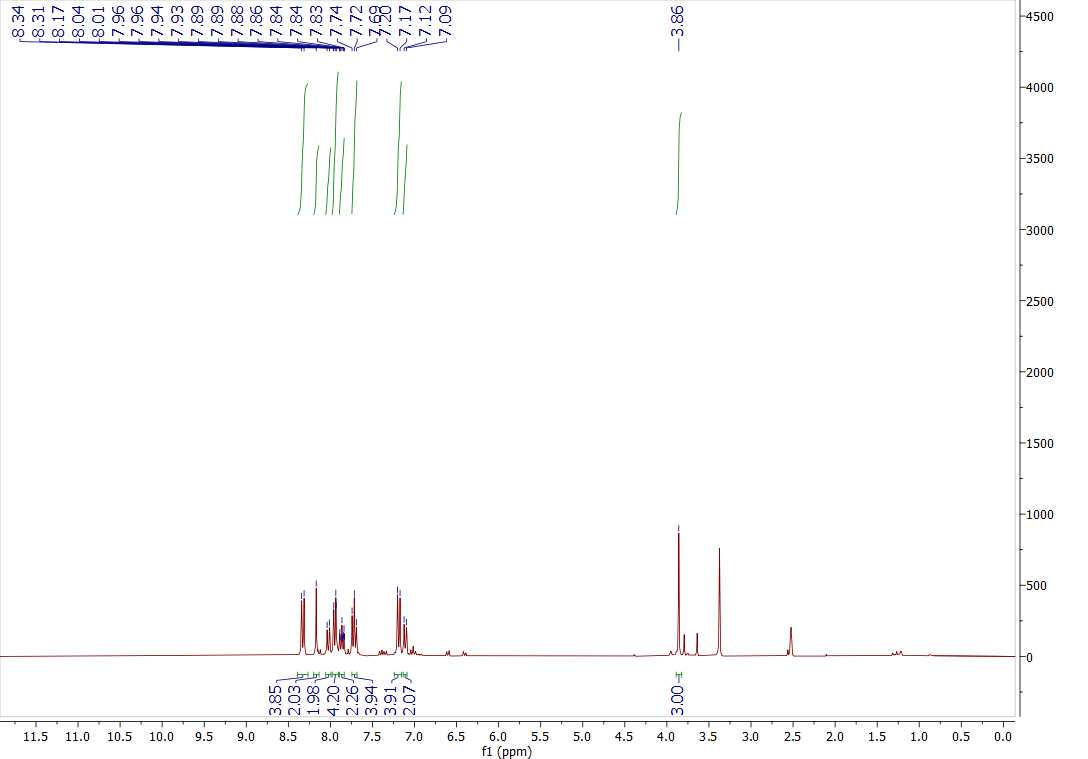


43: ^13^C NMR spectrum of (4-(4-methoxyphenyl)pyridine-2,6-diyl)bis(4,1-phenylene) dibenzenesulfonate (2b)


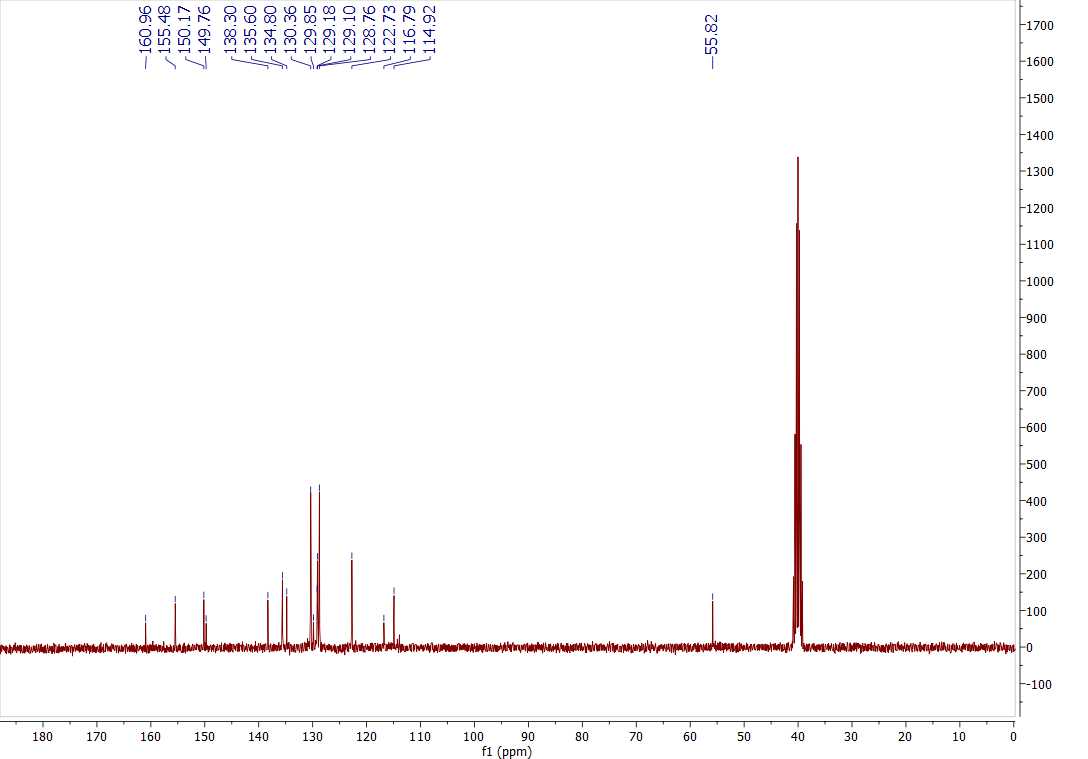


44: FT-IR spectrum of (4-(4-chlorophenyl)pyridine-2,6-diyl)bis(4,1-phenylene) dibenzenesulfonate (2c)


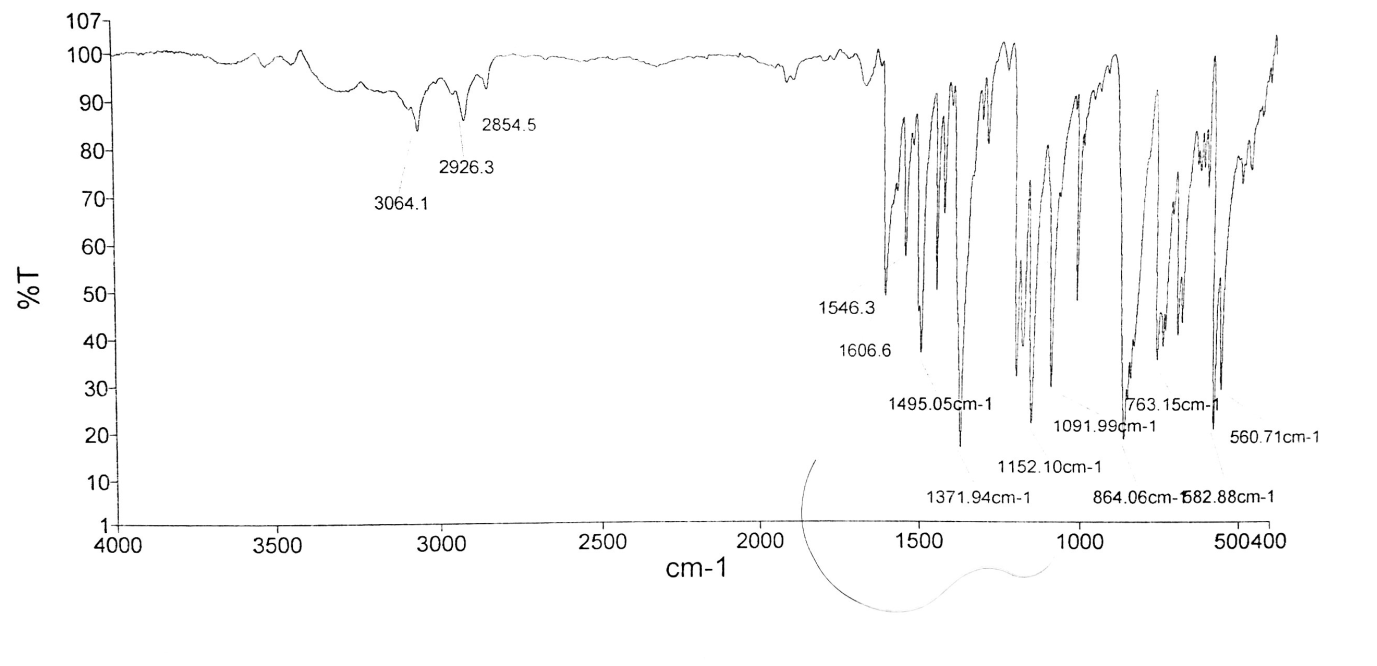


45: ^1^H NMR spectrum of (4-(4-chlorophenyl)pyridine-2,6-diyl)bis(4,1-phenylene) dibenzenesulfonate (2c)


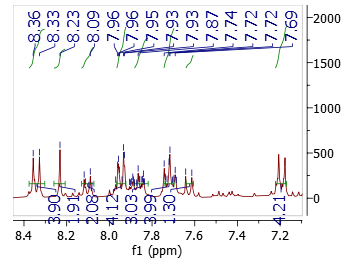

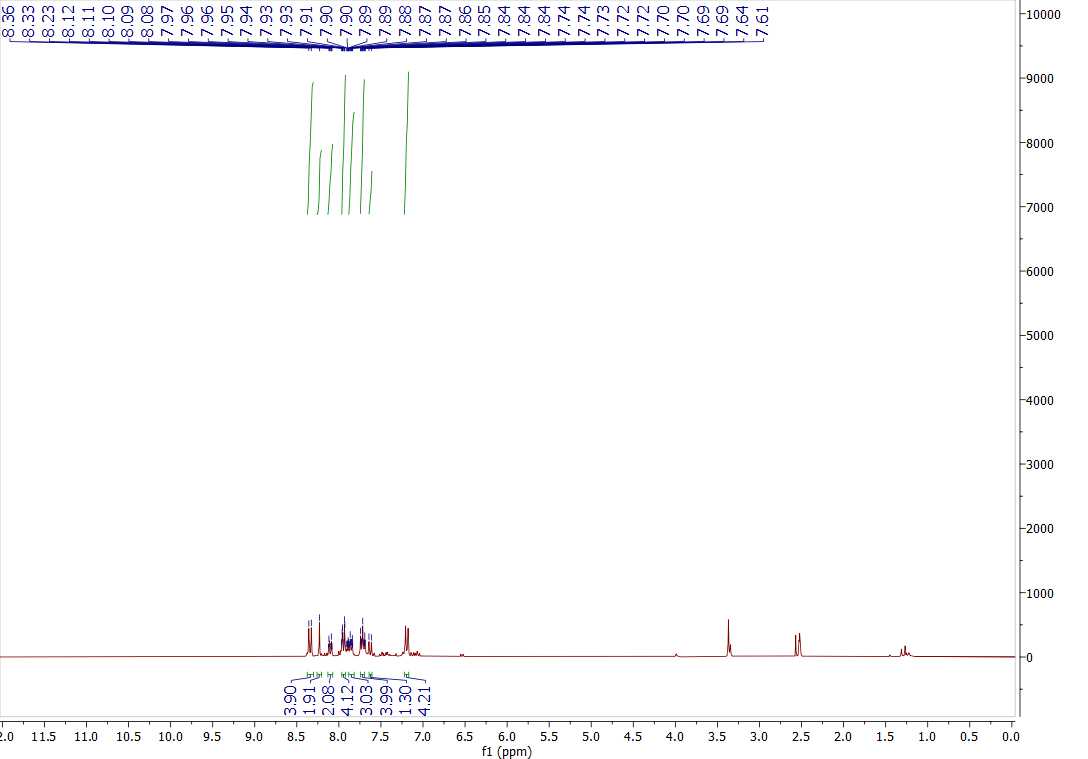


46: ^13^C NMR spectrum of (4-(4-chlorophenyl)pyridine-2,6-diyl)bis(4,1-phenylene) dibenzenesulfonate (2c)


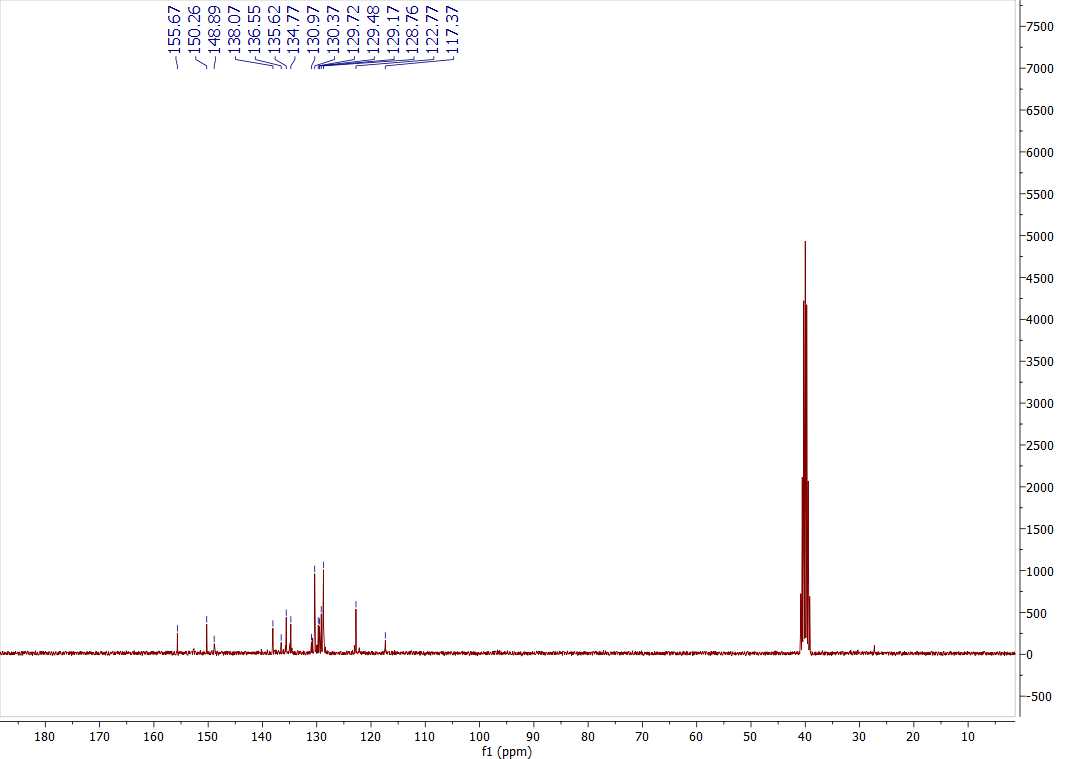


47: FT-IR spectrum of (4-(2-chlorophenyl)pyridine-2,6-diyl)bis(4,1-phenylene) dibenzenesulfonate (2d)


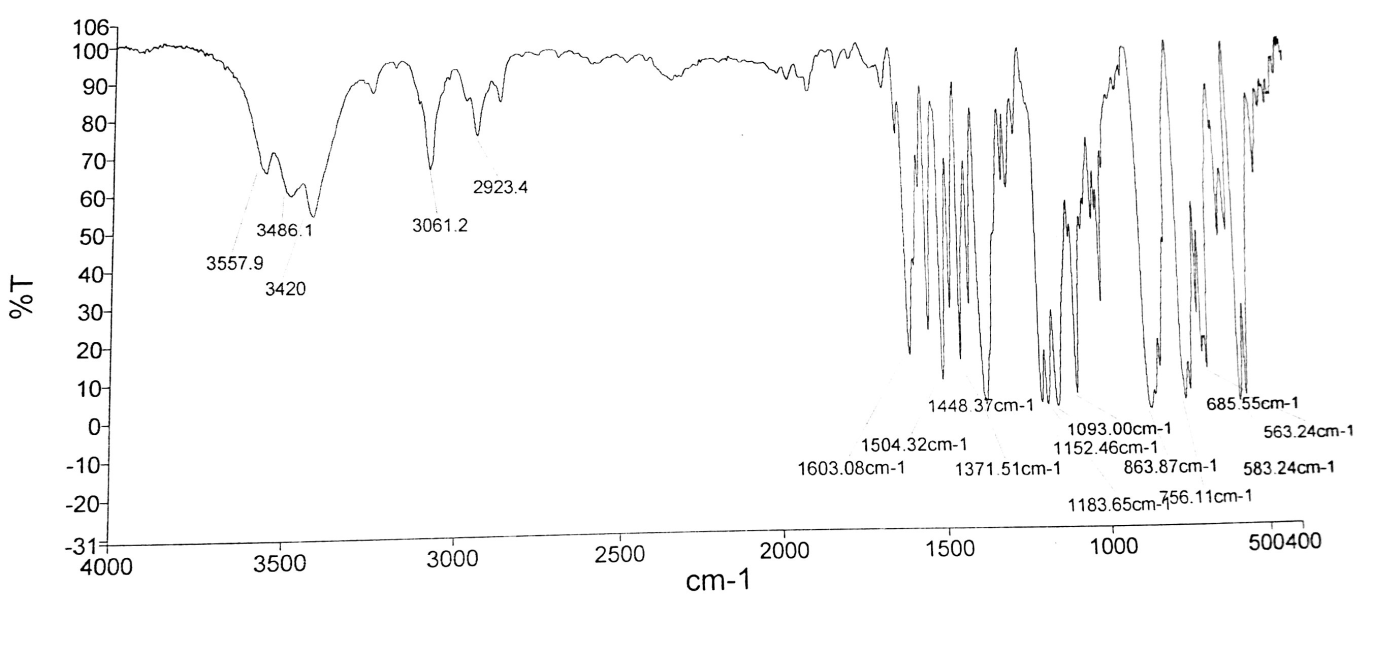


48: ^1^H NMR spectrum of (4-(2-chlorophenyl)pyridine-2,6-diyl)bis(4,1-phenylene) dibenzenesulfonate (2d)


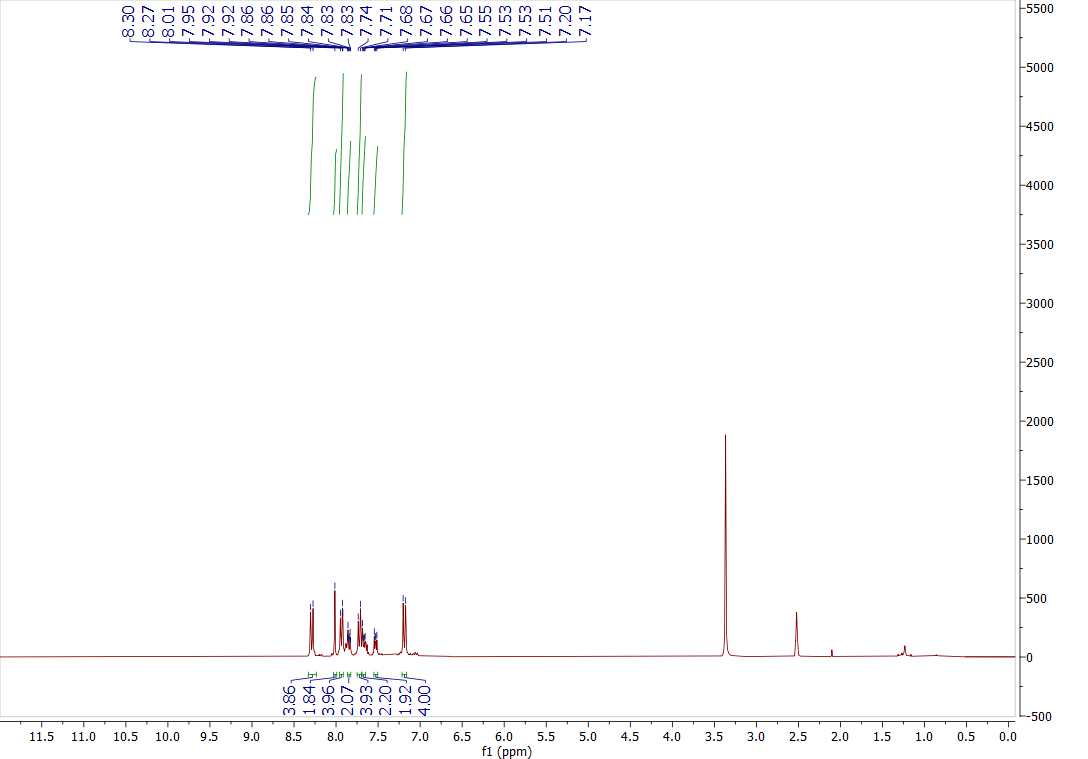


49: ^13^C NMR spectrum of (4-(2-chlorophenyl)pyridine-2,6-diyl)bis(4,1-phenylene) dibenzenesulfonate (2d)
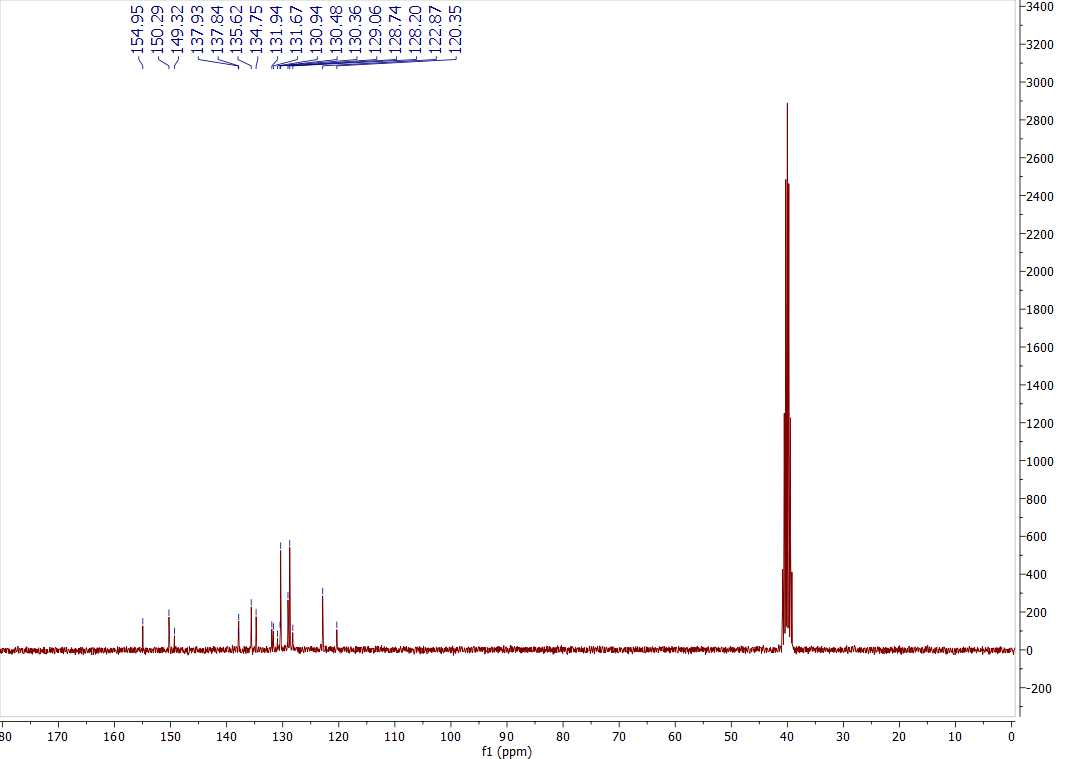

50: FT-IR spectrum of *N*,*N'*-((4-phenylpyridine-2,6-diyl)bis(4,1-phenylene))bis(4-methylbenzenesulfonamide) (3a)


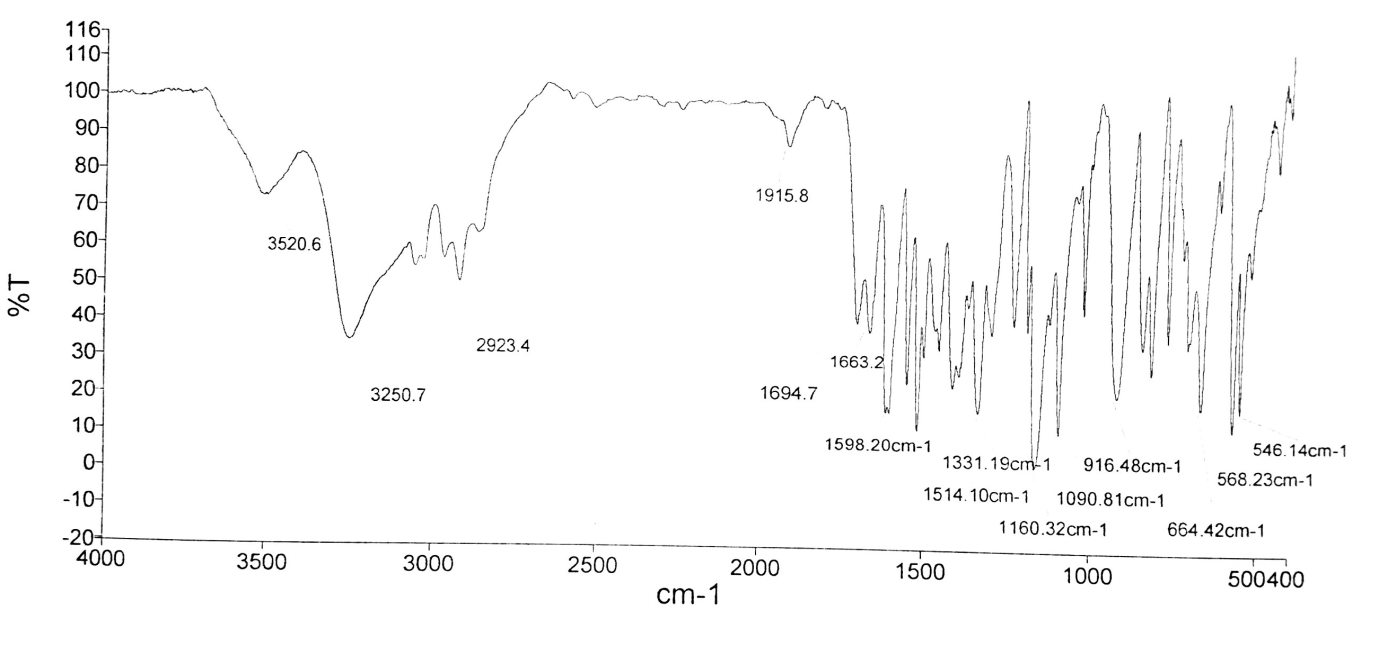


51: ^1^H NMR spectrum of *N*,*N'*-((4-phenylpyridine-2,6-diyl)bis(4,1-phenylene))bis(4-methylbenzenesulfonamide) (3a)


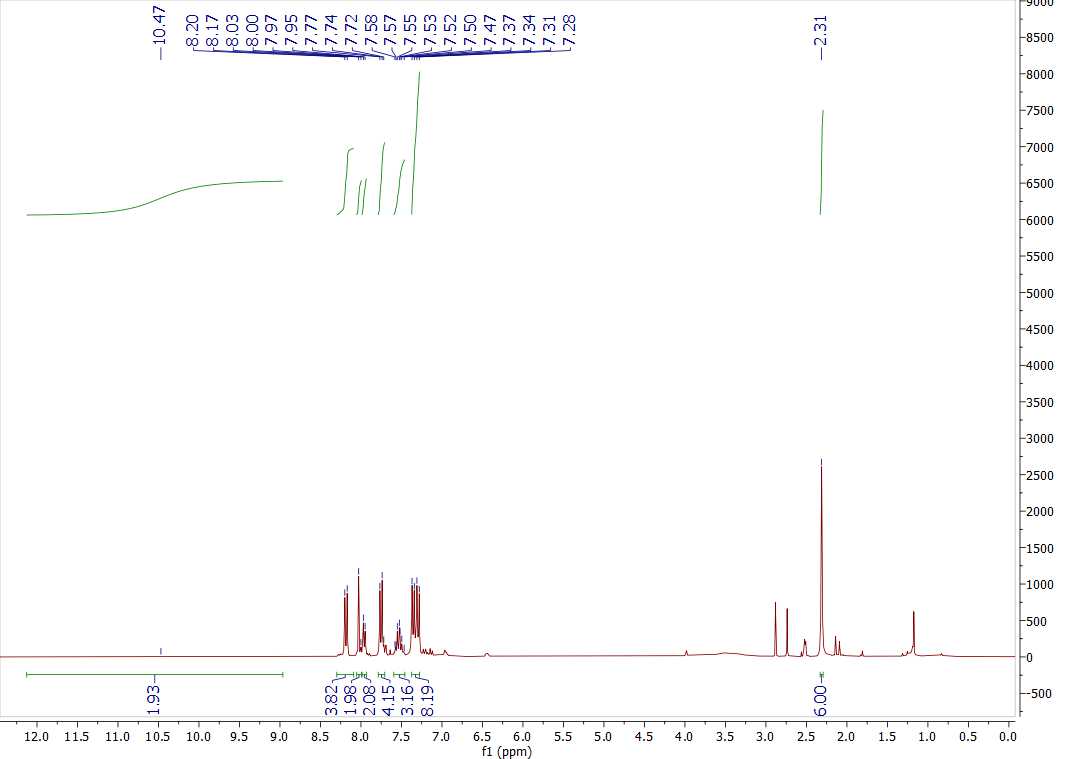


52: ^13^C NMR spectrum of *N*,*N'*-((4-phenylpyridine-2,6-diyl)bis(4,1-phenylene))bis(4-methylbenzenesulfonamide) (3a)


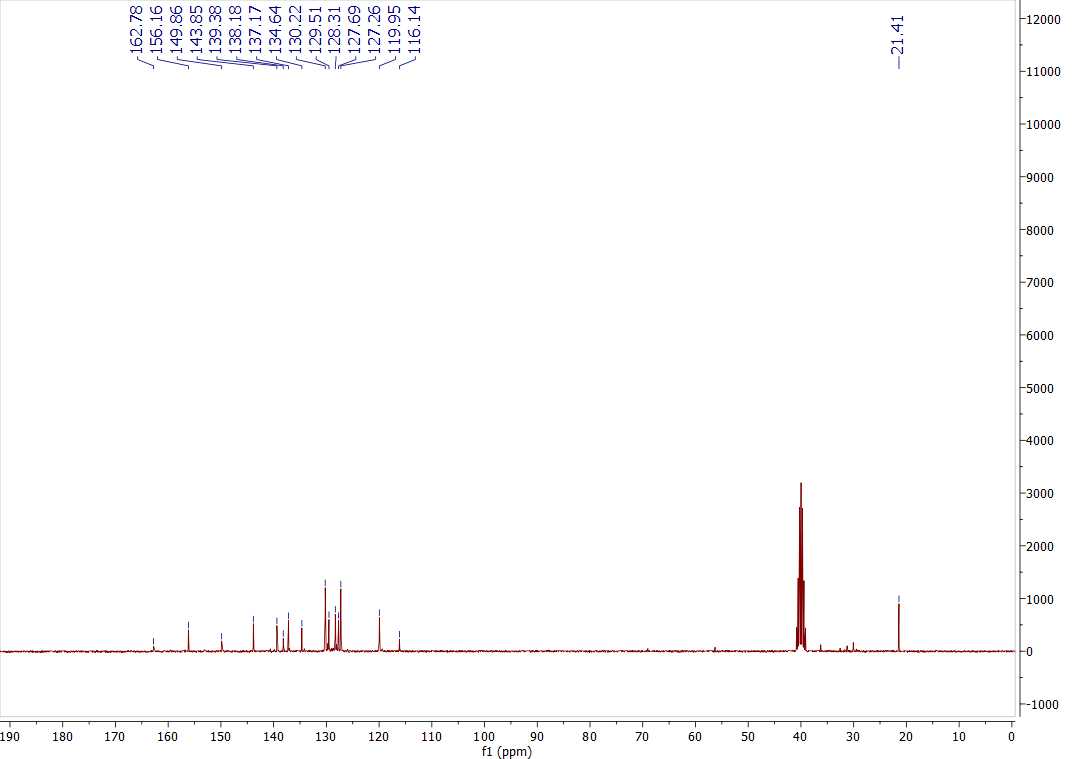


53: FT-IR spectrum of *N*,*N'*-((4-(4-chlorophenyl)pyridine-2,6-diyl)bis(4,1-phenylene))bis(4-methylbenzenesulfonamide) (3b)


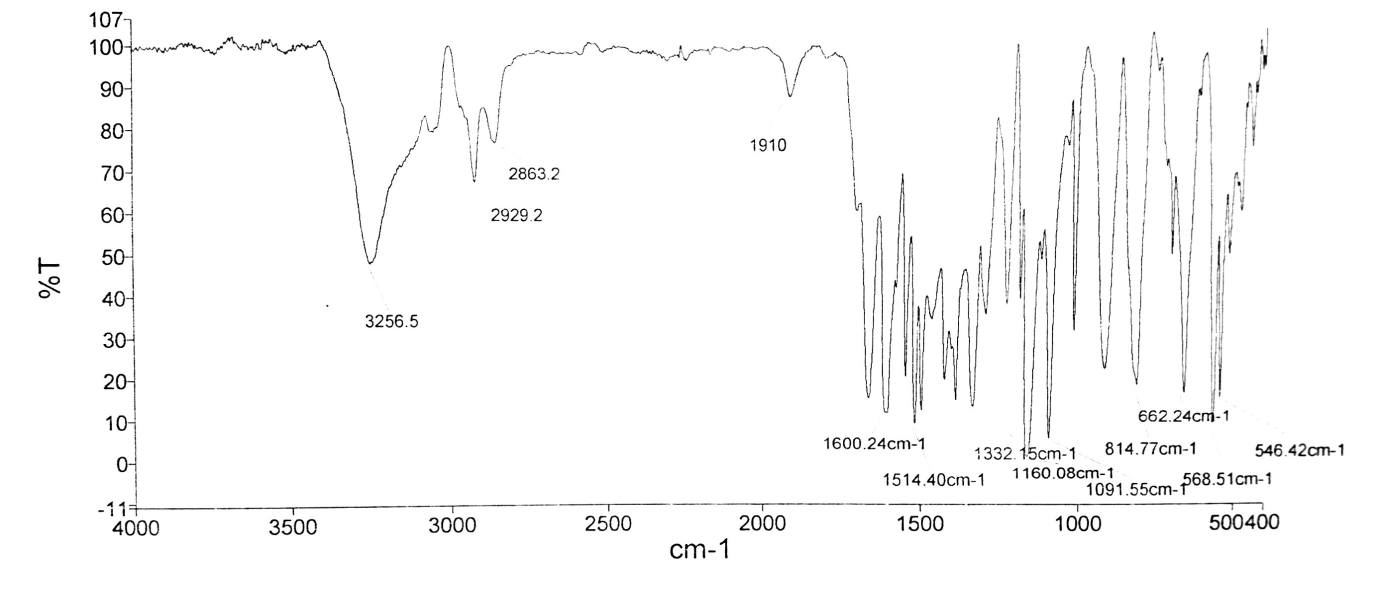


54: ^1^H NMR spectrum of *N*,*N'*-((4-(4-chlorophenyl)pyridine-2,6-diyl)bis(4,1-phenylene))bis(4-methylbenzenesulfonamide) (3b)


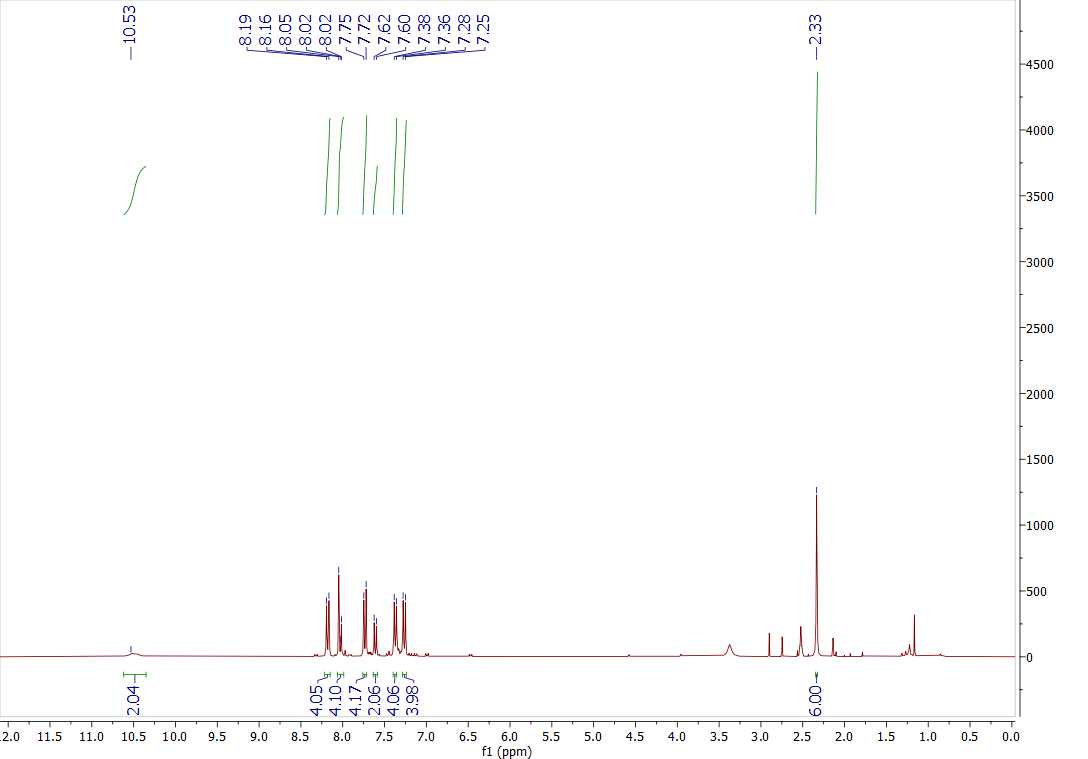


55: ^13^C NMR spectrum of *N*,*N'*-((4-(4-chlorophenyl)pyridine-2,6-diyl)bis(4,1-phenylene))bis(4-methylbenzenesulfonamide) (3b)


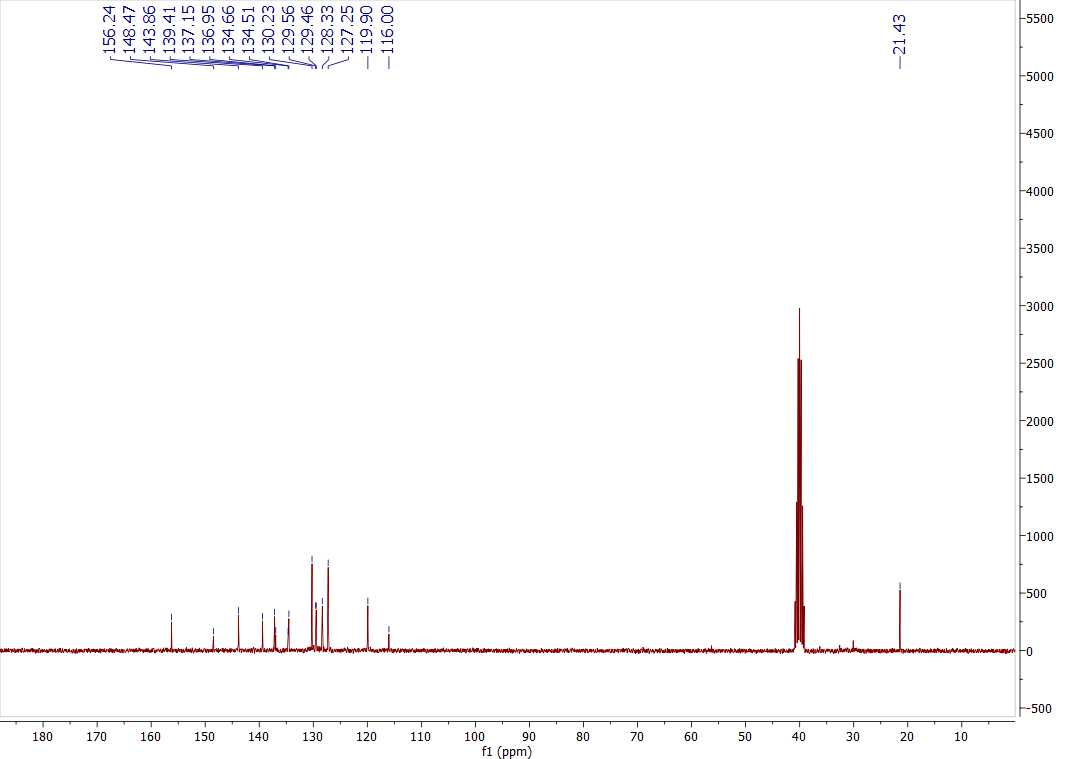


56: Mass spectrum of *N*,*N'*-((4-(4-chlorophenyl)pyridine-2,6-diyl)bis(4,1-phenylene))bis(4-methylbenzenesulfonamide) (3b)


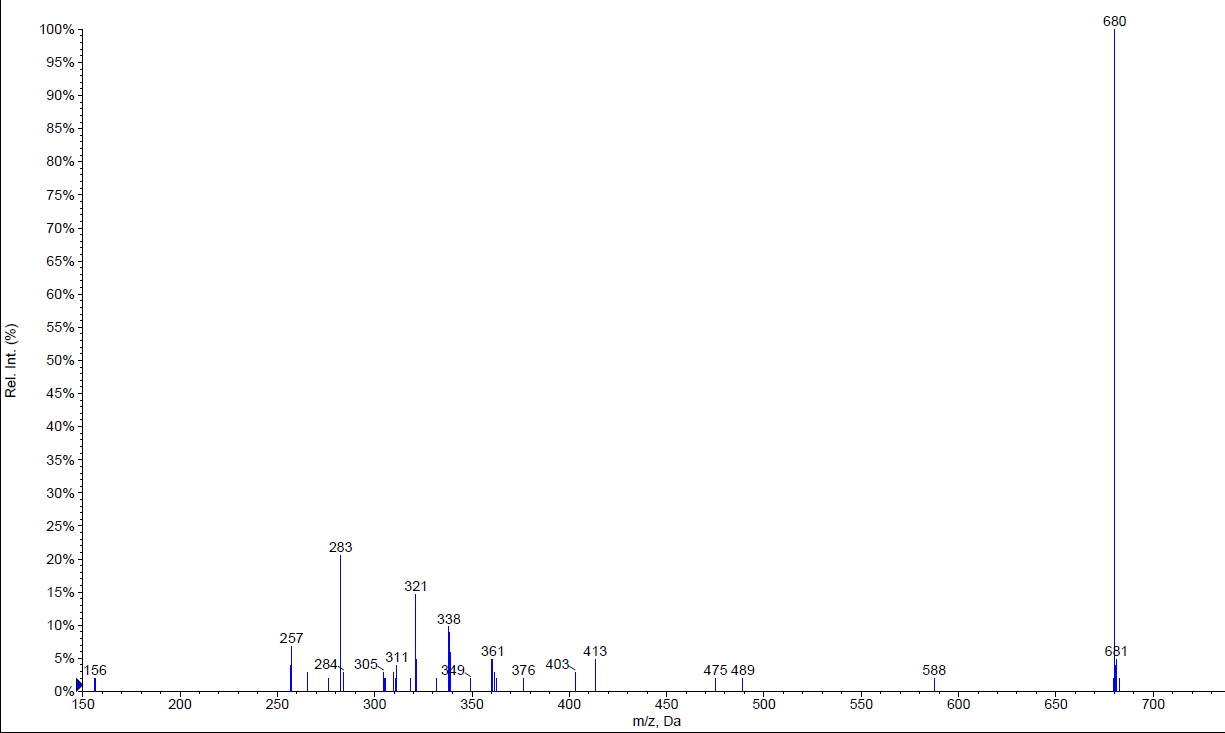


57: FT-IR spectrum of *N*,*N'*-((4-(4-bromophenyl)pyridine-2,6-diyl)bis(4,1-phenylene))bis(4-methylbenzenesulfonamide) (3c)


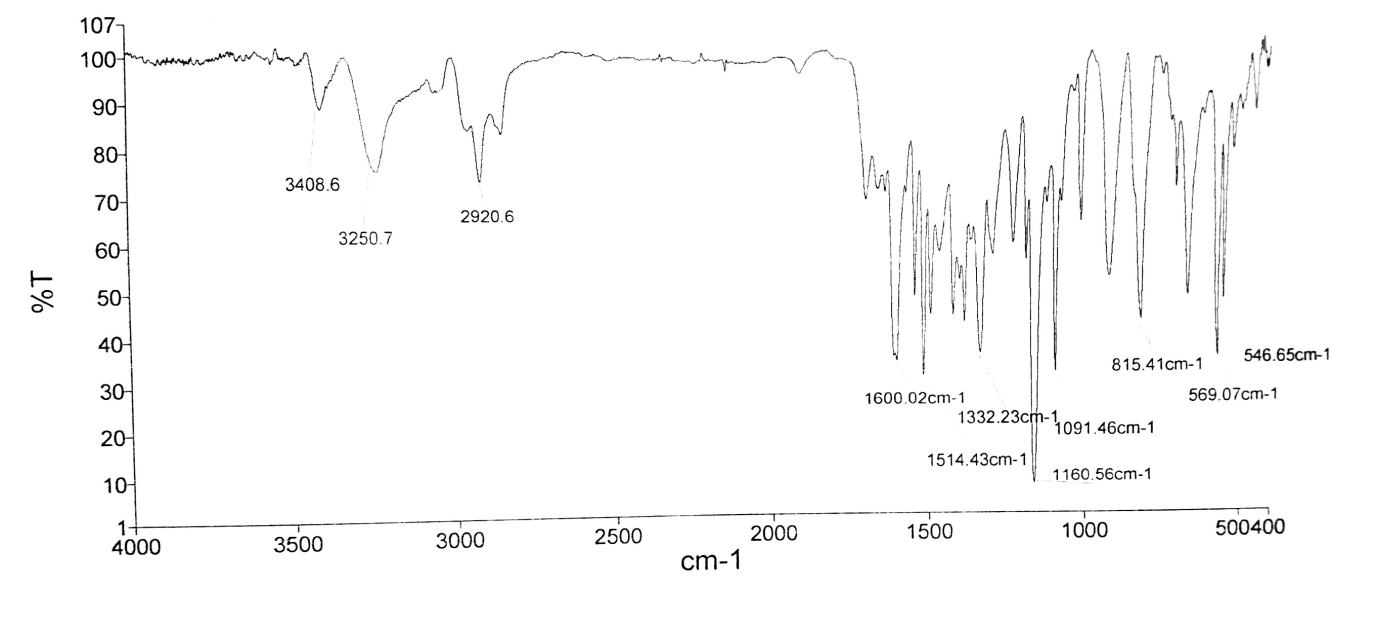

58: ^1^H NMR spectrum of *N*,*N'*-((4-(4-bromophenyl)pyridine-2,6-diyl)bis(4,1-phenylene))bis(4-methylbenzenesulfonamide) (3c)
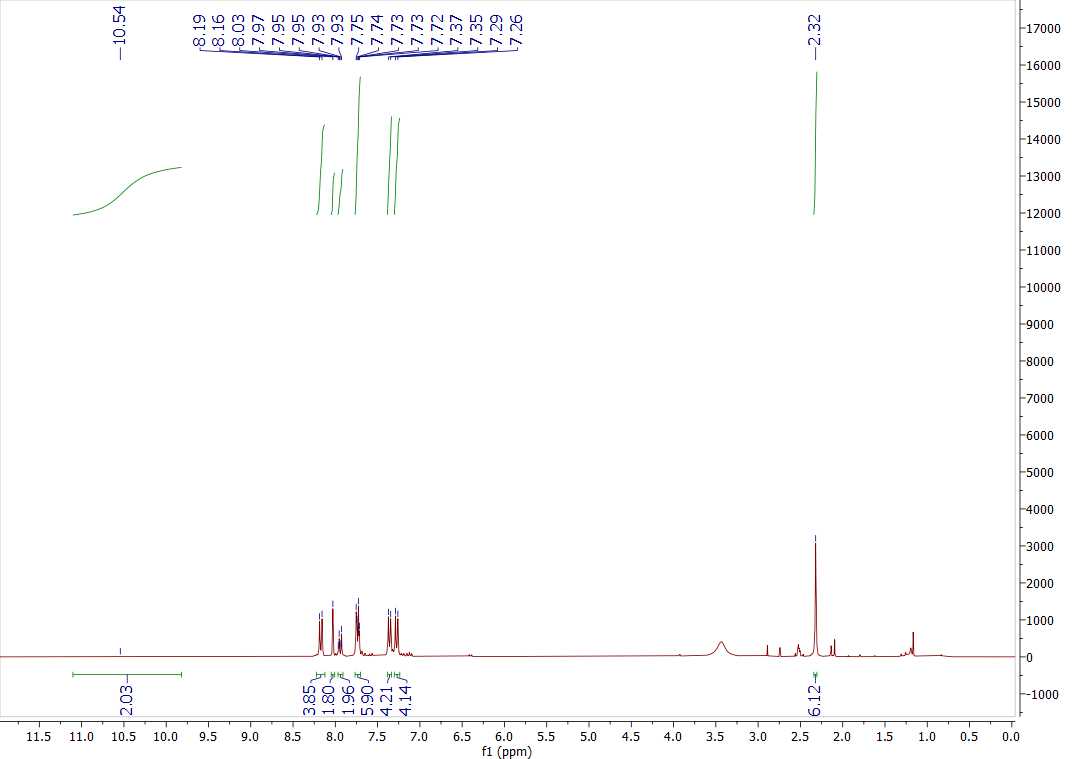


59: ^13^C NMR spectrum of *N*,*N'*-((4-(4-bromophenyl)pyridine-2,6-diyl)bis(4,1-phenylene))bis(4-methylbenzenesulfonamide) (3c)


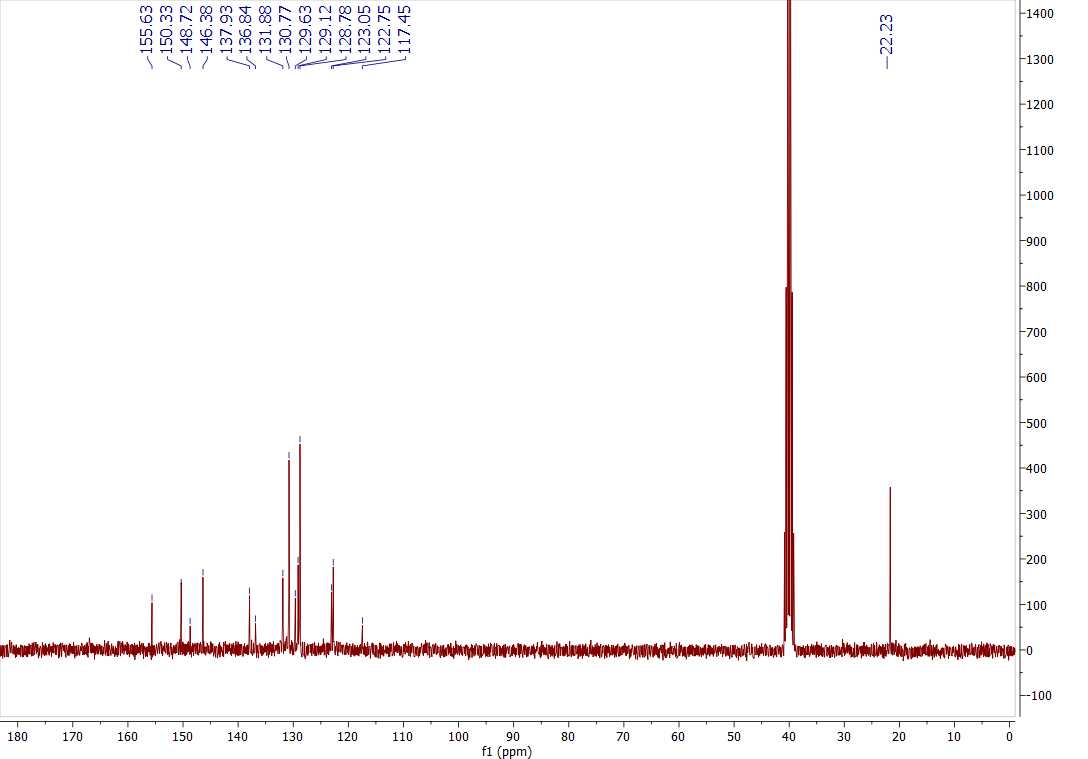


60: FT-IR spectrum of (1,4-phenylenebis(pyridine-4,2,6-triyl))tetrakis(benzene-4,1-diyl) tetrakis(4-methylbenzenesulfonate) (1h)


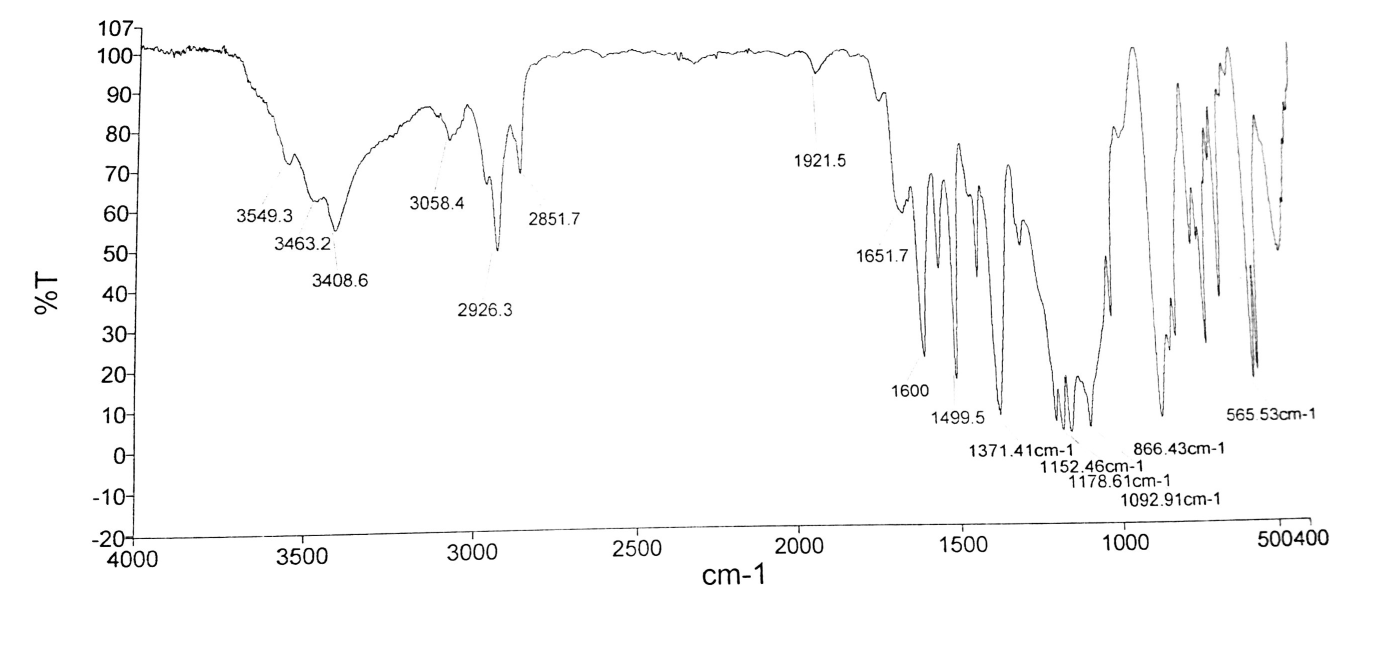


61: ^1^H NMR spectrum of (1,4-phenylenebis(pyridine-4,2,6-triyl))tetrakis(benzene-4,1-diyl) tetrakis(4-methylbenzenesulfonate) (1h)


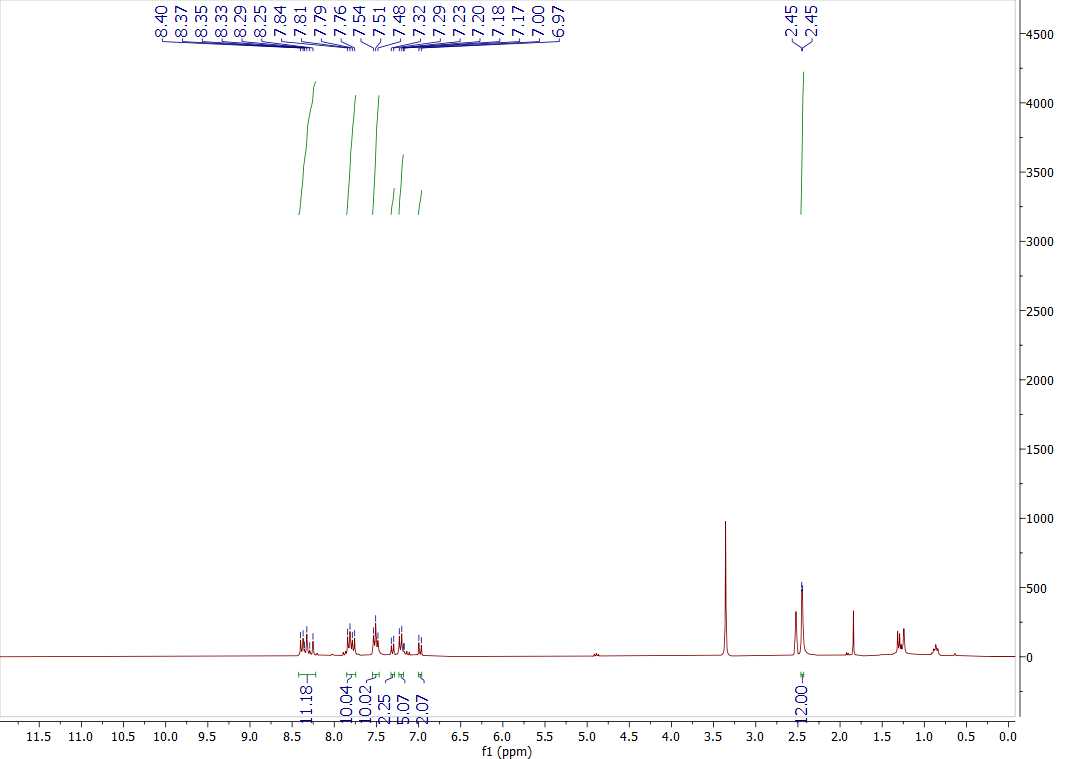
62: ^13^C NMR spectrum of (1,4-phenylenebis(pyridine-4,2,6-triyl))tetrakis(benzene-4,1-diyl) tetrakis(4-methylbenzenesulfonate) (1h)
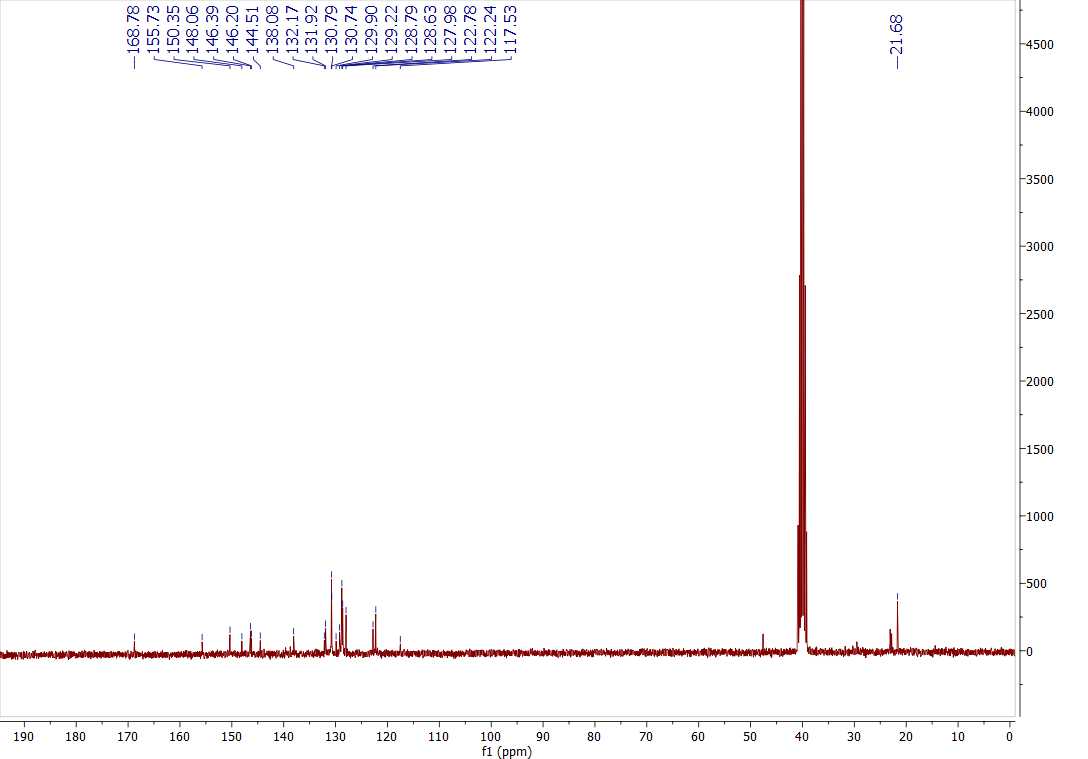

63: Mass spectrum of (1,4-phenylenebis(pyridine-4,2,6-triyl))tetrakis(benzene-4,1-diyl) tetrakis(4-methylbenzenesulfonate) (1h)
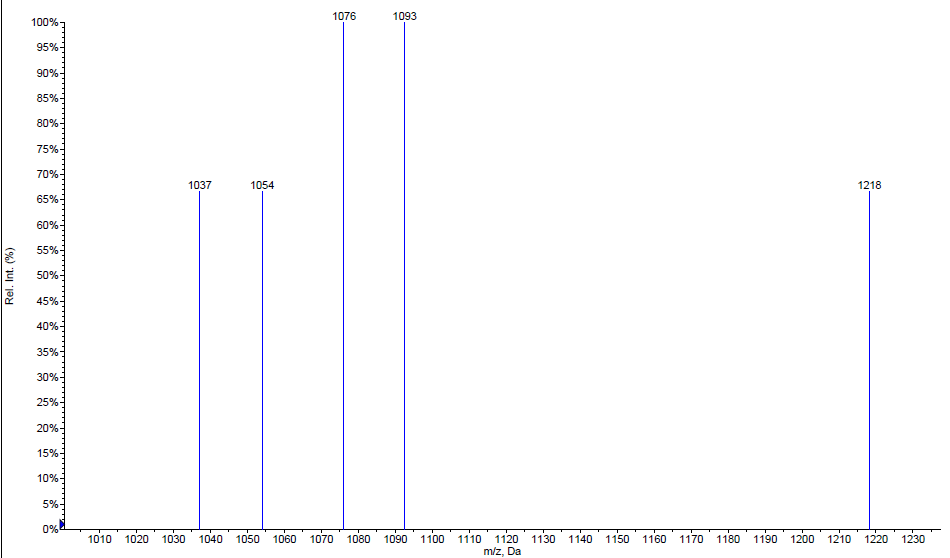


64: FT-IR spectrum of *N,N',N'',N'''*-((1,4-phenylenebis(pyridine-4,2,6-triyl))tetrakis(benzene-4,1-diyl))tetrakis(4-methylbenzenesulfonamide) (3d)


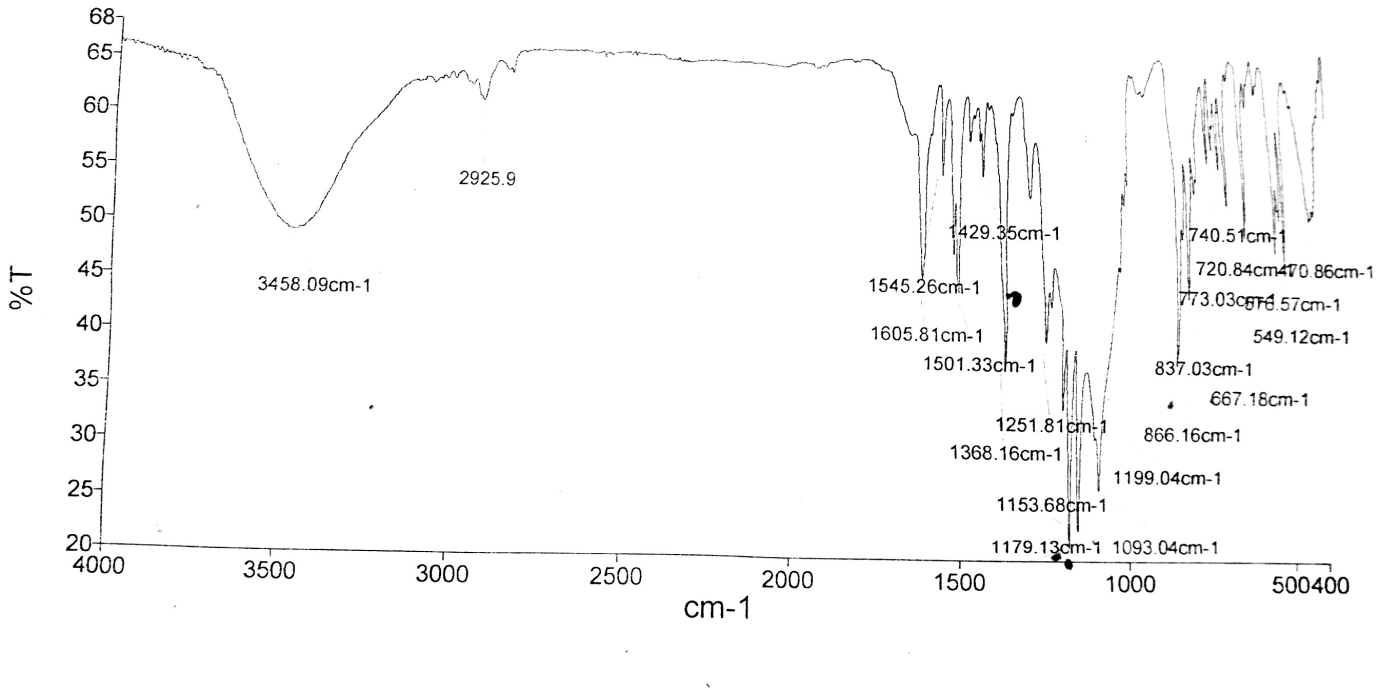


65: ^1^H NMR spectrum of *N,N',N'',N'''*-((1,4-phenylenebis(pyridine-4,2,6-triyl))tetrakis(benzene-4,1-diyl))tetrakis(4-methylbenzenesulfonamide) (3d)


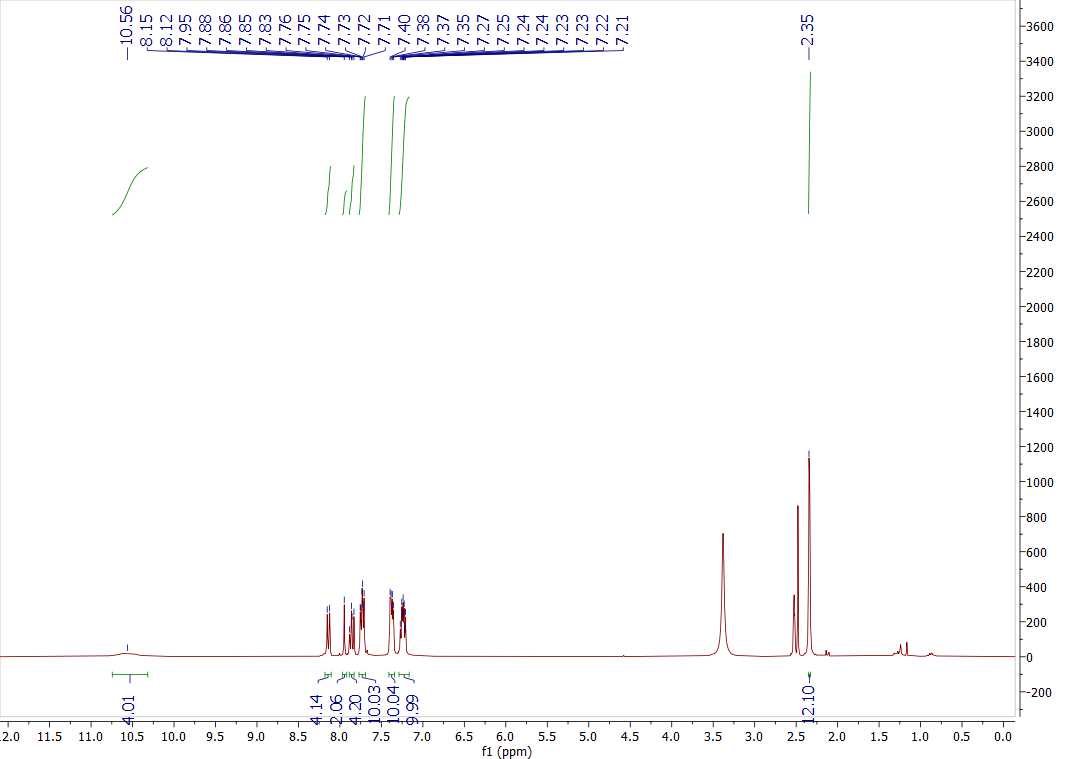


66: ^13^C NMR spectrum of *N,N',N'',N'''*-((1,4-phenylenebis(pyridine-4,2,6-triyl))tetrakis(benzene-4,1-diyl))tetrakis(4-methylbenzenesulfonamide) (3d)


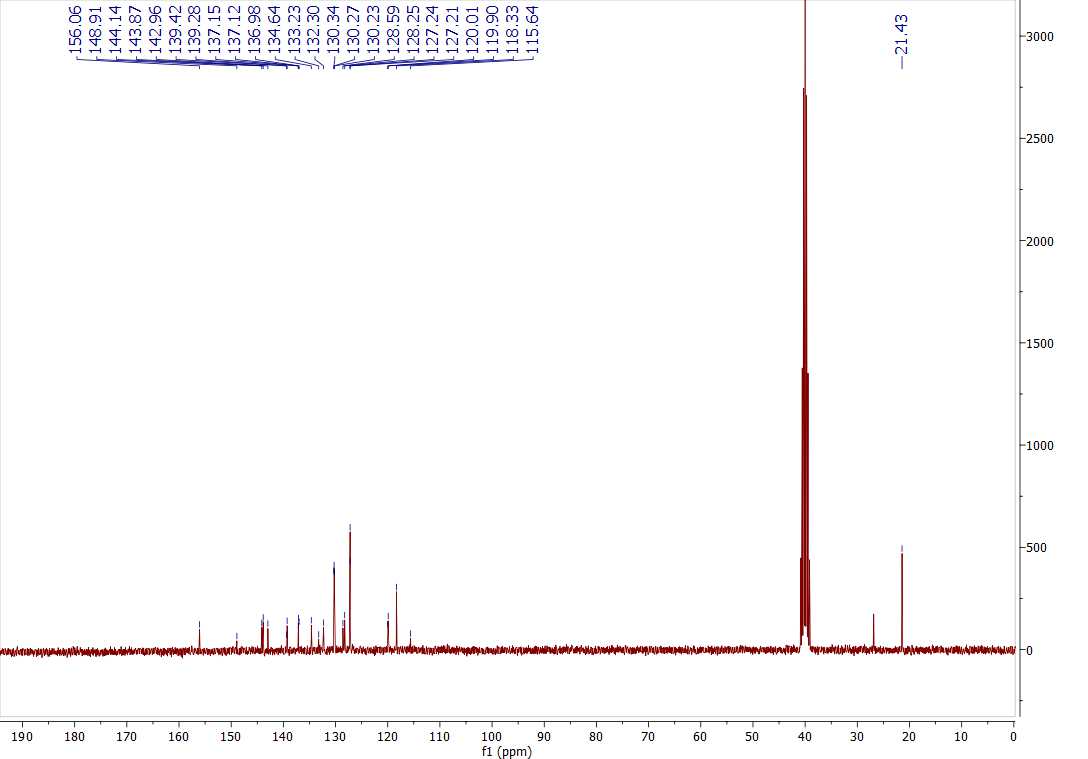


67: ^1^H NMR spectrum of 4,4'-methylenebis(5-methyl-1H-pyrazol-3-ol


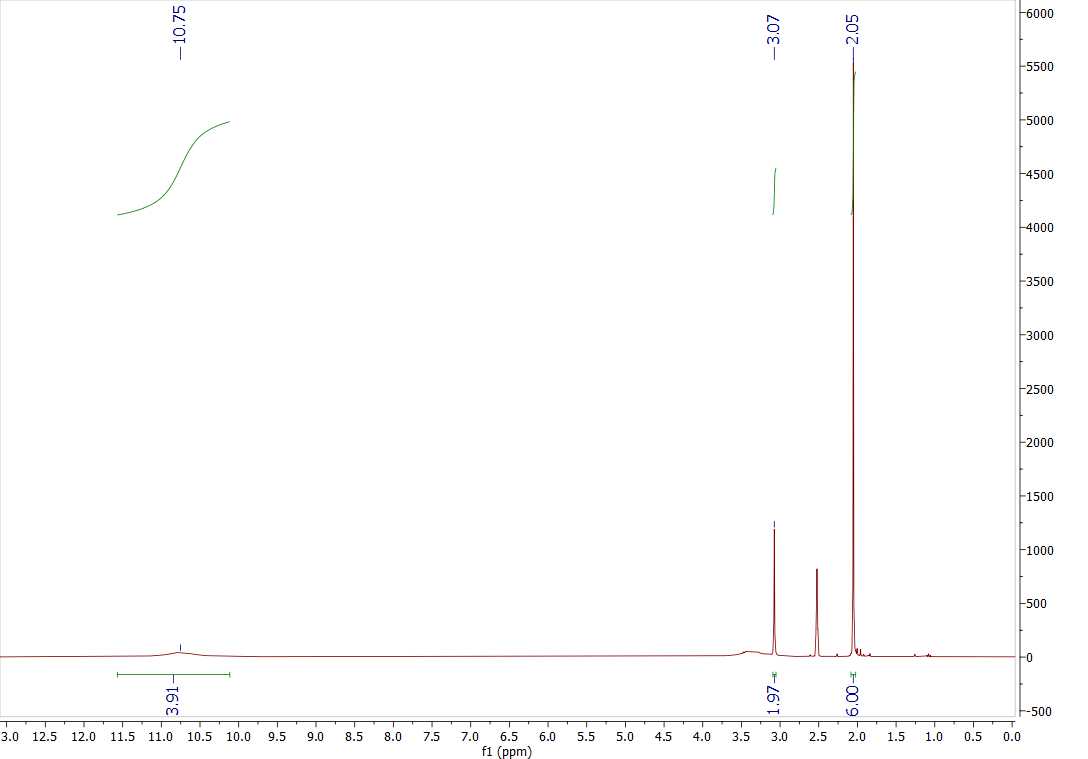


68: ^13^C NMR spectrum of 4,4'-methylenebis(5-methyl-1H-pyrazol-3-ol


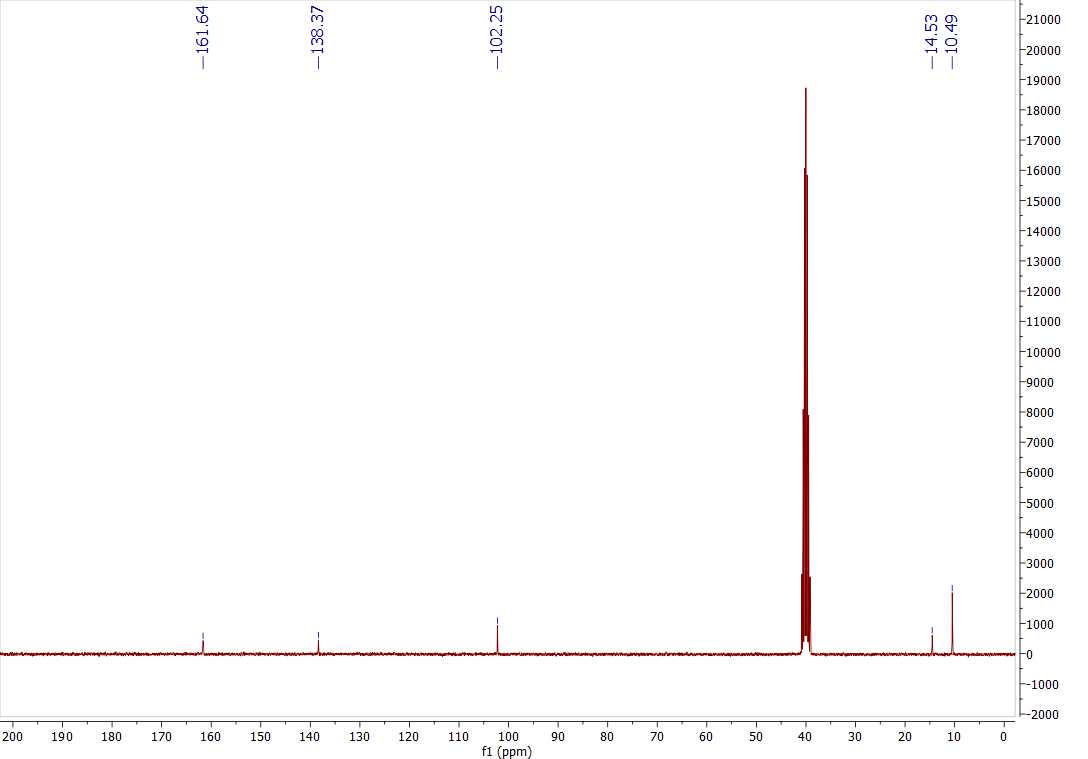


69: XRD pattern of Fe_3_O_4_@SiO_2_@PCLH-TFA


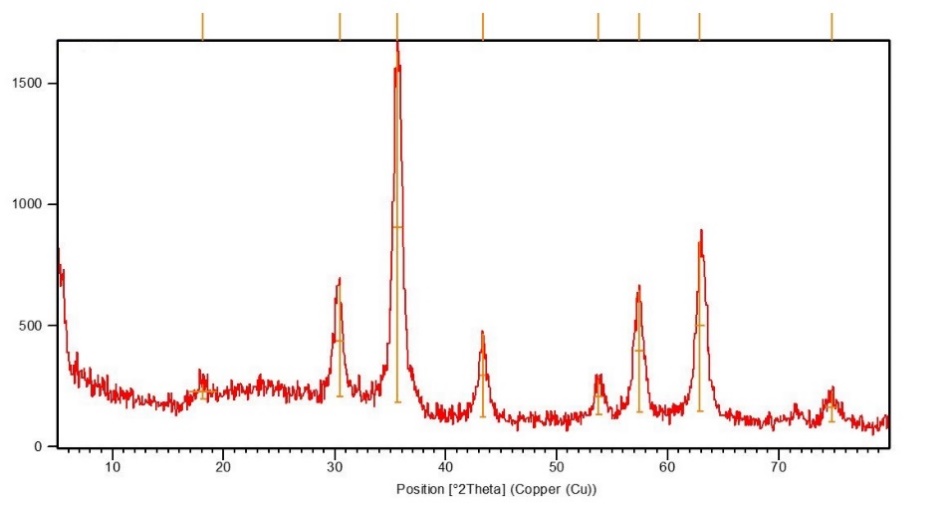

Supplement: Supplementary file 1 — Supplementary Information. [file 41598_2021_95830_MOESM1_ESM.docx]
